# A theoretical, dynamical evaluation method of the steric hindrance in nitroxide radicals using transition states of model reactions

**DOI:** 10.1038/s41598-019-56342-w

**Published:** 2019-12-30

**Authors:** Yudai Yamazaki, Jun Naganuma, Hiroaki Gotoh

**Affiliations:** Department of Chemistry and Life Science, Yokohama National University, 79-5 Tokiwadai, Hodogaya-ku, Yokohama Japan

**Keywords:** Organic chemistry, Theoretical chemistry

## Abstract

Steric hindrance is known to affect the stability, reactivity, and radical trapping ability of stable nitroxide radicals. Therefore, a quantitative evaluation and prediction model of steric hindrance is needed to select and design the optimum nitroxide radicals for specific applications. In this study, a dynamic parameter of steric hindrance (DPSH) is proposed and its characteristics are investigated. Unlike using only the equilibrium structure to evaluate the steric hindrance, DPSH is a dynamic value calculated from the theoretical activation enthalpies for two model reactions of radical addition to olefins. Using DPSH, the steric hindrance was evaluated for a total of 43 alkyl radicals, nitroxide radicals, and radicals derived from phenols, and the results were compared with those of other methods. The DPSH values for radicals can vary when the energy barrier for structural change varies, even if the equilibrium structures of the radicals have the same steric shielding. Finally, for radicals other than nitroxide radicals, the DPSH values were consistent with the predictions from their structures, which suggests that the DPSH has a wide range of applications. We expect DPSH to be used and developed in the analysis of steric factors in various reactions.

## Introduction

Steric factors affect chemical properties as much as electronic factors do. For example, the tertiary butyl (*t*-Bu) group suppresses the inversion of the cyclohexane ring due to its bulkiness, and S_N_2 reactions are less likely to occur in trisubstituted carbons because of steric hindrance. Furthermore, the impact of steric factors has been confirmed in a wide range of areas, including optical activity without chiral center^1–3^, strong non-nucleophilic bases^4,5^, stereoselective reactions^6^, and the antioxidant activities of substituted phenols such as butylated hydroxytoluene^7^.

Nitroxide radicals, which are known to be stable radical species, have been studied for various applications, including enhancing stability against reduction^8–10^, radical trapping^11,12^, catalysts in organic synthesis^13–15^, and nitroxide-mediated radical polymerization^16,17^. Notably, the effect of steric hindrance in the reaction center (which in this paper refers to the atom that does not satisfy the octet rule in the structural formula) is considered to be important in these applications. Therefore, methods of quantifying steric hindrance are not only useful for the theoretical elucidation and design of new nitroxide radicals, but are also expected to lead to the development of methods that could describe steric factors in general molecules.

Hitherto, the quantification of steric factors has been carried out both experimentally and theoretically. In Taft’s pioneer study, a parameter E_s_, which was based on the rates of esterification and hydrolysis, was proposed as an indicator of substituent bulkiness^18,19^. Later, E_s_ was developed into E_s_’ (including more varied substituents using the unified reaction) and E_s_^c^ (taking into consideration the hyperconjugation effect) by Duboi *et al*.^20^ and Hancock *et al*.^21^, respectively. Furthermore, Fujita *et al*.^22^ introduced a method in which the E_s_^c^ for the substituent CR^1^R^2^R^3^ was expressed by a linear combination of the E_s_^c^ values of R^1^, R^2^, and R^3^. Based on this study, Bagryanskaya *et al*.^23^ and Kirilyuk *et al*.^10^ evaluated the steric hindrance of the nitroxide radical using the E_s_^c^ values of the substituents located around the reaction center and used these values to analyze the reaction rate. This evaluation, based only on E_s_^c^ values, is easy to apply to various nitroxide radicals but does not include factors arising from other parts of the molecules, such as the skeleton, bond lengths, and angles. Notably, in Tolman’s work^24^, the steric hindrance related to the phosphorus ligand of a metal complex was quantified by the apex angle in the imaginary cone circumscribing the ligand, and the angle was defined as a parameter corresponding to the congestion around the binding site. This is called the Tolman cone angle and can be used to analyze the ability of various phosphorus ligands to bind to Ni^24^.

Furthermore, evaluation methods that use imaginary objects to study molecules have also been investigated. Hirota *et al*. developed a method to calculate the area of the shadow produced by an atom blocking the light emitted from a virtual light source^25–27^. The area Ω_S_ calculated by this method was used to evaluate the steric hindrance of the substituent, and the excellent correlation between Ω_S_ and both the E_s_ value and logarithm of the reaction rate constant were recognized^25^. Tomilin *et al*. used a phantom sphere to assess the steric hindrance of radical species^28^. In their work, the populations of atoms contained in a sphere centered on the reaction centers of carbon, nitrogen, and oxygen radicals were used to calculate a new steric hindrance parameter γ in an effort to analyze radical dimerization reactions. Although their method required multiple calculations with different radiuses of spheres, it was, in fact, easy to handle in terms of using the populations of atoms. Meanwhile, Cavallo *et al*. used the volume occupied by atoms rather than the number of atoms to evaluate the steric hindrance^29^. In order to evaluate the steric hindrance of *N*-heterocyclic ligands, the percentage of atoms in the ligand occupying an imaginary sphere centered on the metal atom to which the *N*-heterocyclic ligand was bonded was calculated and defined as %V_Bur_, which was used to analyze the bond dissociation enthalpy between the ligand and metal. Similarly, in our recent study, the steric shielding of 70 nitroxide radicals was quantified using the indicator V_SS_, which was defined from the atomic volume within a hypothetical sphere centered on the reaction center of the nitroxides^30^. Although this method does not consider the positions of the atoms within the sphere, it is easily applicable to various molecules as it uses a calculated chemical structure.

Differently from the methods introduced so far, the method reported by Lee *et al*. proposed the concept of an accessible surface area (ASA) to study protein structures and folding^31^. The ASA was defined as the locus that is drawn by the center of a sphere representing a solvent molecule as it moves around while touching the surface of the target molecule, which is expressed as a combination of spheres with radii equal to the van der Waals radii of its constituent atoms. The ASA has since been used by Yamasaki *et al*. to evaluate steric factors around the reaction centers of nitroxide radicals^8^.

Notably, experimental and theoretical evaluations have different advantages. The former are expected to be easy to apply to real systems because the factors involved in actual reactions, such as structural changes in molecules and various interactions, are inherently included in experimental approaches. In contrast, the latter are expected to be useful for the design of new molecules and broad research that targets many molecules, because they can handle a large number of molecules and even not-yet-synthesized or unstable molecules, such as reaction intermediates, more easily than experimental methods. However, most of the conventional theoretical methods have scope for improvement as they are static and focus on the equilibrium structures of molecules.

In view of the above, in this study, we computationally evaluated the steric hindrance of predominantly nitroxide radicals via a method focusing on transition states and the enthalpy of activation. We then compared these results with the equilibrium structures and the results obtained from other evaluation methods and reactivity experiments, and further investigated the factors influencing our steric hindrance evaluation.

**Scheme 1:**
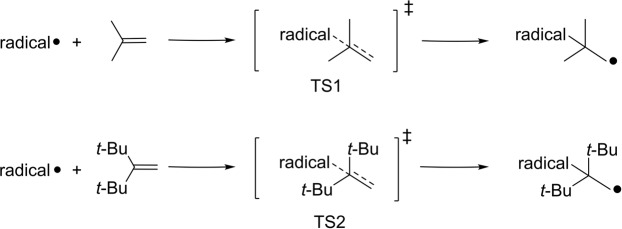
Model addition reactions and their transition states used in the quantitative evaluation of steric hindrance.

In our method, we first used the addition reaction of a radical to two olefins (Scheme 1), which is a common reaction, to evaluate the steric hindrance of the radical. In fact, addition reactions of nitroxide radicals to olefins have already been reported^32,33^. Second, the activation enthalpies Δ*H*_1_^‡^ and Δ*H*_2_^‡^ were calculated for the transition states TS1 and TS2 of the addition reactions, respectively. Third, the difference between the calculated activation enthalpies, Δ*H*_2_^‡^-Δ*H*_1_^‡^, was defined as a dynamic parameter of steric hindrance (DPSH). Molecular force field and semi-empirical molecular orbital methods, as well as density functional theory, were used as calculation methods. (U)B3LYP and (U)M06-2X were used as the functionals and vacuum conditions were assumed in all calculations.

DPSH is defined as the increase in activation enthalpy that is caused by an increase in the bulkiness of olefin substituents. We used DPSH as an indicator of the steric hindrance in the reaction centers of radicals because the increase in activation enthalpy is expected to grow with the steric hindrance at the reaction center.

DPSH has two features: (1) it focuses on the transition states, which makes it a dynamic assessment of the steric hindrance involved in the reaction, and (2) it uses differences in the activation enthalpy of two different transition states to exclude the electronic factors. In steric hindrance evaluations that use the activation enthalpy of a single reaction, both steric and electronic factors are included. In this study, the contribution of the electronic factor is expected to be the same between the two addition reactions because they both generate the same kind of single bond. Therefore, we aimed to eliminate the electronic factor by considering the difference in activation enthalpy for the two reactions.

## Materials and Methods

**Figure 1:**
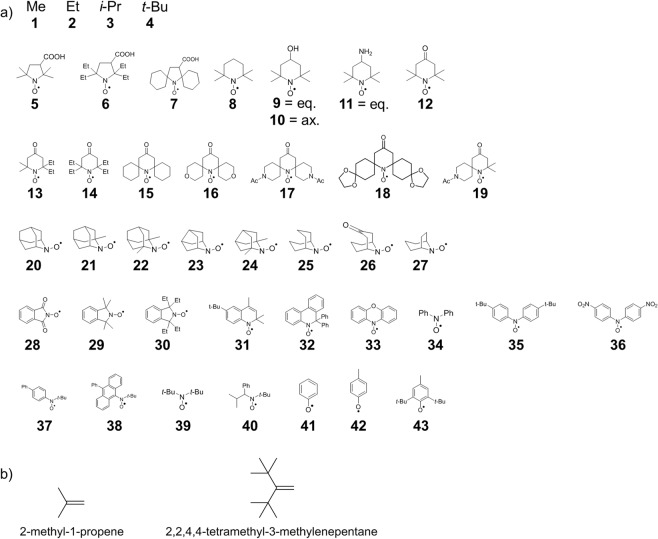
(**a**) Radicals investigated in this study: 4 alkyl radicals (**1–4**), 36 nitroxide radicals (**5–40**), and 3 phenoxy radicals (**41–43**). Equatorial and axial conformations are indicated by eq. and ax., respectively. (**b**) Olefins used in this study: 2-methyl-1-propene and 2,2,4,4-tetramethyl-3-methylenepentane.

In this study, the steric hindrance of the reaction center was quantified for 4 types of alkyl radicals, 36 types of nitroxide radicals, and 3 types of phenoxy radicals derived from phenol, *p*-cresol, and butylated hydroxytoluene (Fig. 1). Figure 1 also shows the olefins used for the evaluation of the steric hindrance.

The equilibrium structures of these 43 radicals and the two olefins were obtained by molecular force field calculations and density functional theory. First, based on the MM3 molecular force field^34^, a stochastic conformation search^35^ in which 2000 conformations were generated and optimized, and the one with the lowest energy was chosen, was performed using the Medit program^36^. Second, the conformation with the lowest energy obtained thereby was optimized at the (U)B3LYP/6-31 G* level using the Gaussian 09 program^37^, and additional optimization of the obtained structures was performed at the (U)M06-2X/6-31 G* level. The optimized structures at each calculation level were taken as the equilibrium structures at each level. The S^2^ values were also calculated by structure optimizations. The orbital energy and the total energy of the nuclei and electrons in the nitroxide radicals and olefins were calculated for the equilibrium structures by single-point calculations at the ROB3LYP/6-311 + G** and (U)B3LYP/6-311 + G** level, respectively. Furthermore, V_SS_ values of the equilibrium structure for the resulting nitroxide radicals were calculated by the same method as in the previous study^30^. A virtual sphere was installed at the reaction center of nitroxide radicals represented by the close-packed model, and the atomic volume enclosed by this sphere was defined as V_SS_.

Transition states were determined by a semi-empirical molecular orbital method and density functional method. First, using the Winmostar program^38^ and MOPAC program^39^, maximal energy structures that were considered as candidate transition states were obtained through minimum energy path calculations using PM3^40^ or AM1^41^ as the Hamiltonian. The transition states were then obtained by optimizing the maximum energy structures or structures prepared by considering previously calculated transition states of other molecules at the UB3LYP/6-31G* level using the Gaussian 09 program^37^; additional optimization of the obtained structures was also carried out at the UM06-2X/6-31G* level. Finally, vibrational calculations were performed at the same level on the obtained structures to confirm that they had only one imaginary frequency in the direction related to the addition reaction. The paths from the transition state to the original and generative systems were also confirmed using intrinsic reaction coordinate calculations. Furthermore, the total energy of the nuclei and electrons was obtained by a single-point calculation at the (U)B3LYP/6-311 + G** level for the following structures: the transition state, containing the nitroxide radical and olefin; the transition state structures of the nitroxide radical; and the transition state structures of the olefins. In addition, the V_SS_ values^30^ of the transition state structures of the nitroxide radicals were also calculated.

The activation enthalpy was obtained from that of the transition state minus the sum of those of the equilibrium-state nitroxide radical and olefin. Each enthalpy was calculated at 25 °C by performing the same level of vibration calculation as that used in the structural optimization for the equilibrium structure or transition state with the Gaussian 09 program^37^.

The calculation results, including structures, S^2^ values, total energy of nuclei and electrons, orbital energy, enthalpy, and wavenumbers of the imaginary frequencies, are described in the supplementary information (SI).

## Results and Discussion

Table 1 shows the DPSH value, its value relative to a methyl radical, and the V_SS_ value. The relative DPSH was calculated by dividing the absolute DPSH value for each radical by that of methyl radical; both DPSH values were obtained using the same functional. In this paper, the relative value according to (U)B3LYP/6-31 G* level calculation is treated as the DPSH unless otherwise stated. Among the nitroxide radicals, the minimum DPSH value, 0.990, and the maximum value, 4.15, were obtained for **28** and **40**, respectively. Table 1 also includes the calculated steric hindrance value based on E_s_^c^ (hereafter referred to as E_s_^c^) obtained from our study^30^.

**Table 1:** Calculated DPSH, relative DPSH, V_SS_, and E_s_^c^ values of the studied radicals.

Entry	DPSH/kJ mol^−1^	Relative value/−	${{\bf{E}}}_{{\bf{s}}}^{{\bf{c}}}$/−	V_SS_/Å^3^	Entry	DPSH/kJ mol^−1^	Relative value/−	${{\bf{E}}}_{{\bf{s}}}^{{\bf{c}}}$/−	V_SS_/Å^3^
**1**	23.6 (11.0)	1.00 (1.00)	—	—	**21**	49.6 (28.9)	2.10 (2.61)	—	12.5
**2**	37.8	1.60	—	—	**22**	71.7 (52.2)	3.04 (4.73)	—	14.6
**3**	59.9	2.54	—	—	**23**	31.9	1.35	—	10.2
**4**	99.2	4.21	—	—	**24**	72.4	3.07	—	14.2
**5**	57.8	2.45	−4.20	14.2	**25**	33.4	1.41	−2.94	10.8
**6**	71.1	3.01	−6.20	18.2	**26**	33.3	1.41	—	10.8
**7**	61.5	2.61	−5.00	15.4	**27**	29.8	1.26	—	10.2
**8**	71.3	3.02	−4.20	14.4	**28**	23.4	0.990	—	6.83
**9**	71.3	3.02	−4.20^a^	14.4	**29**	57.3	2.43	−4.20	14.4
**10**	71.2	3.02	−4.20^a^	14.4	**30**	68.5	2.90	−6.20	17.4
**11**	71.2	3.02	−4.20	14.4	**31**	44.3	1.88	—	11.6
**12**	70.1	2.97	−4.20	14.3	**32**	69.5	2.95	—	14.0
**13**	80.8	3.43	−5.21	15.8	**33**	45.8	1.94	—	8.68
**14**	92.1	3.90	−6.20	17.2	**34**	46.0	1.95	—	8.72
**15**	78.4	3.33	−5.00	15.5	**35**	45.9	1.94	—	8.69
**16**	77.5	3.29	—	15.3	**36**	45.5	1.93	—	8.72
**17**	63.2	2.68	—	15.4	**37**	39.0	1.65	—	11.1
**18**	62.3	2.64	—	15.5	**38**	58.3	2.47	—	13.8
**19**	62.5	2.65	—	14.9	**39**	75.4	3.19	−4.20	14.1
**20**	32.5 (12.6)	1.38 (1.14)	—	10.5	**40**	97.9	4.15	—	16.5
					**41**	23.4	0.992	—	—
					**42**	23.9	1.01	—	—
					**43**	90.9	3.85	—	—

^a^As equatorial and axial conformations were not distinguished in ref. ^30^, they were given the same value in this paper.

^b^The values in parentheses are according to (U)M06-2X/6-31 G* level calculations. The others are according to (U)B3LYP/6-31 G* level calculations.

As can be seen from Table 1, an increase in the number of substituents around the reaction center caused an increase in the DPSH. For example, the DPSH increased continuously from alkyl radical **1** to radical **4**, and an increase in the number of methyl (Me) groups caused an increase in the DPSH of **20**–**22** and **23**–**24**, respectively, for nitroxide radicals. This result is reasonable as it corresponds to an increase in the steric shielding in the reaction center by the introduction of a Me group. Moreover, although the DPSH values for **20**–**22** based on the (U)M06-2X calculations are different from those based on the (U)B3LYP calculations, they also increased as the number of Me groups increased, which suggests that other methods can be used to calculate the DPSH. For example, M06 might be used for various systems, including metal ions. It should be useful to investigate the effect of using different calculation methods in future studies and applications. The substituents far from the reaction center had almost no effect on the DPSH, as confirmed from the results for **8**–**12**, **25**–**26**, and **34**–**36**. Each group had the same skeleton and different substituents at positions far from the reaction center, but the DPSH was almost equal for these radicals. This indicates that the substituents far from the reaction center barely affected the steric hindrance of the reaction center.

These results also showed that the DPSH is independent of electronic factors. Introducing a substituent changes the electronic factor of the molecule, which is reflected in the change in the single occupied molecular orbital (SOMO) energy in **34**–**36**, shown in Table 2. Introducing an electron donating group (*t*-Bu) increased the SOMO energy, while an electron withdrawing group (nitro group) decreased it. However, there was little change in the DPSH, which suggests the successful exclusion of electronic factors.

**Table 2:** DPSH, activation enthalpies, and SOMO energy for entry **34–36**.

Entry	DPSH/−	${\boldsymbol{\Delta }}{{\boldsymbol{H}}}_{{\bf{2}}}^{{\boldsymbol{\ddagger }}}{\boldsymbol{-}}{\boldsymbol{\Delta }}{{\boldsymbol{H}}}_{{\bf{1}}}^{\ddagger }{\boldsymbol{/}}{\bf{kJ}}\,{{\bf{mol}}}^{{\boldsymbol{-}}{\bf{1}}}$	Δ*H*_1_^‡^/kJ mol^−1^	Δ*H*_2_^‡^/kJ mol^−1^	SOMO/eV
**34**	1.95	46.0	110	156	−2.75
**35**	1.94	45.9	112	157	−2.62
**36**	1.93	45.4	92.0	137	−3.60

The activation enthalpy of each reaction changed with the SOMO level of the nitroxide radical, but the DPSH values shown in Table 2 did not change at all with the SOMO level. This indicates that defining the DPSH by the difference in activation enthalpies contributed to removing the electronic factor from the DPSH. The activation enthalpy decreased with the introduction of electron withdrawing groups, presumably because the decrease in the SOMO energy increased the orbital interaction of the olefin with the nitroxide radical. As a result, if the evaluation of the steric hindrance was performed using either Δ*H*^‡^_1_ or Δ*H*^‡^_2_, the DPSH value of **36** would be considerably smaller than that of the other two. However, utilizing the difference between the two activation enthalpies suppressed the variation in the DPSH value since the variation in the activation enthalpy due to the change of a substituent was almost the same for both olefins, which supports the prediction that the electronic factors are expected to be the same in both of the addition reactions.

The DPSH of **37**–**40** and their equilibrium structures, which are displayed in the rod model in Table 3, showed that in addition to the substituents near the reaction center, the structure around the reaction center also contributed to the steric hindrance. The DPSH increased in response to the increase in hydrogen atoms sterically close to and encircling the reaction center (marked by red circles in Table 3). If only the substituents around the reaction center were considered, the steric hindrance of **40**, which has fewer substituents around the reaction center, would be smaller than that of **39**. However, the DPSH value of **40** was larger than that of **39**, which could be explained by the fact that more atoms surrounded the reaction center in **40** than in **39**.

**Table 3:** DPSH values and calculated equilibrium structures for entry **37–40**.

				
Entry	**37**	**38**	**39**	**40**
DPSH^/−^	1.65	2.47	3.19	4.15
			

Notably, **5**, **8**–**12**, **22**, **24**, **25**, **27**, **29**, and **39**, which are similar nitroxide radicals that have Me groups or hydrogen atoms around the reaction center, were selected to investigate the influence of the molecular skeleton on the steric hindrance. Table 4 presents the DPSH values, bond angle Θ (∠C_1_NC_2_), and bond angle Φ (∠OC_2_C_3_) of each radical, which were determined from the equilibrium structure. The Θ and Φ angles are shown in the schematic in Table 4. The angle $\Phi \,$ is not described for **25** and **27**.

**Table 4:** DPSH, Θ, and Φ values for some nitroxides.

Entry	DPSH/−	Θ/°	Φ/°	Entry	DPSH/−	Θ/°
**5**	2.45	116	91.3	**25**	1.41	113
**8**	3.02	125	87.6	**27**	1.26	104
**9**	3.02	125	87.8			
**10**	3.02	125	87.3			
**11**	3.02	125	87.9			
**12**	2.97	125	87.1			
**22**	3.04	116	85.6			
**24**	3.07	118	85.6			
**29**	2.43	116	95.1			
**39**	3.19	128	79.1			

First, **5**, **8**–**12**, **29**, and **39**, which had at least four Me groups in the α position, were considered. A positive correlation with a coefficient of +0.998 was confirmed between the DPSH and Θ values for these radicals. This correlation was interpreted as the carbon at the α position and the substituents bonded to it moving closer to the reaction center as Θ increased. The same explanation applies to **25** and **27**. Second, including **22** and **24** in the group discussed above makes the DPSH difficult to explain in terms of angle Θ, since **22** and **24** have smaller Θ angles compared to other molecules with similar DPSH values. In fact, this discrepancy can be explained in terms of Φ. A negative correlation with a coefficient of −0.863 was confirmed between the DPSH and the angle Φ. This result was interpreted as the substituents being closer to the reaction center in a folded manner as Φ decreased, even though angle Θ did not increase.

Furthermore, the obtained DPSH values were compared with the experimental results in previous reports. Palleta *et al*.^9^ obtained the rate constants shown in Table 5 for the reduction of **5**–**7** to hydroxylamine. Based on visual assessment of the steric hindrance using the space-filling plots of **5–7**, this result was explained by an increase in the steric hindrance, which the DPSH values showed quantitatively.

**Table 5:** Corresponding reduction rate constant and DPSH for entries **5–7**.

Entry	5	6	7
DPSH/−	2.45	3.01	2.61
Reduction rate constant/M^−1^ s^−1^	0.063	≤0.001	0.031

According to the study on radical trapping by nitroxide radicals by Bowry *et al*.^11^, the trapping rate decreases in the following order: **25** ≈ **27** ≫ **29** ≳ **8** > **39**. Also, the trapping rate decreases more sharply in **8** than in **25** as the number of substituents increases in the reactive center of the trapped radical. This result was explained by the increased steric hindrance in the nitroxide radical caused by its structure. Here, the larger steric hindrance of the radicals (**8**, **25**, **27**, **29**, **39**) as predicted by Bowry *et al*. correlated to increasing DPSH values. However, the difference between the DPSH values of **29** and **8** is larger than that between **8** and **39**, partly because the rate of radical trapping is influenced not only by steric hindrance but also by electronic factors. Furthermore, a comparison between **8** and newly developed **38** in our past study^12^ showed that the trapping product of the latter was more thermally stable than that of the former. This was explained by the smaller steric hindrance of **38**, which conforms to the DPSH value.

In the study of nitroxide radical catalysts by Iwabuchi *et al*.^13^, when using NaOCl, **20** and **21** exhibited higher catalytic activity than **8** and **22** for the oxidation of a wide range of not only primary but also secondary alcohols. This result did not correspond to the cyclic voltammetry measurement and was explained by the lower steric hindrance around the reaction center in the former. The DPSH values of **8**, **20**, **21**, and **22** support this interpretation. Also, in their study of catalytic oxidation of alcohols, Steves *et al*.^14^ reported that the activity of C–H bond cleavage in **25** was higher than that in **8** without being affected by the electronic and steric factors of the alcohol because of the lower steric hindrance of **25**. The DPSH values calculated herein agree with this result.

Although nitroxide radicals were the main target in this study, the DPSH is expected to be applicable to other types of radicals. In principle, the DPSH does not have a factor limiting its scope to nitroxide radicals since various other radicals can also undergo addition reactions to olefins. Furthermore, in this study, the DPSH values for alkyl and phenoxy radicals were also found to correspond with their structure. For example, as already mentioned, the DPSH of alkyl radicals increased with the increase in the number of substituents attached to the reaction center. In phenoxy radicals (**41**–**43**), introducing a substituent to the *p*-position far from the reaction center hardly changed the DPSH, but introducing substituents to the *o*-position near the reaction center caused the DPSH to increase, which is a reasonable result because substituents near the reaction center often increase the steric hindrance. Therefore, we expect the DPSH to be applicable to other radical species, such as nitrogen-centered radicals like 1,1-diphenyl-2-picrylhydrazyl and growing terminal radicals generated by radical polymerization.

**Figure 2:**
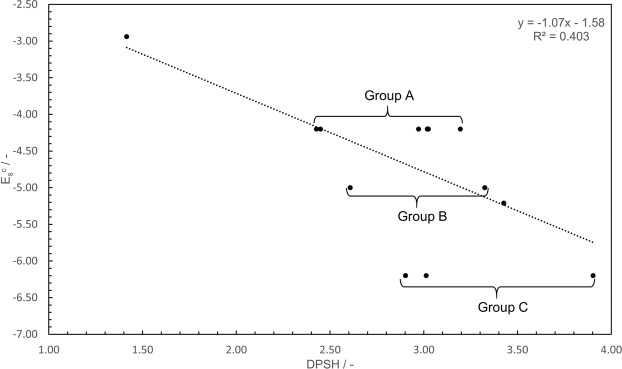
Plot of E_s_^c^ *versus* DPSH and the corresponding line of best fit.

Furthermore, the characteristics of the DPSH were investigated by comparing it to other parameters used to assess steric hindrance, such as E_s_^c^. Figure 2 shows a plot of E_s_^c^ *versus* DPSH for the nitroxide radicals whose E_s_^c^ values are given in Table 1. A weak negative correlation with a coefficient of −0.635 was observed. The negative coefficient is consistent with an increase in steric hindrance corresponding to an increase in DPSH and a decrease in E_s_^c^, respectively.

**Figure 3:**
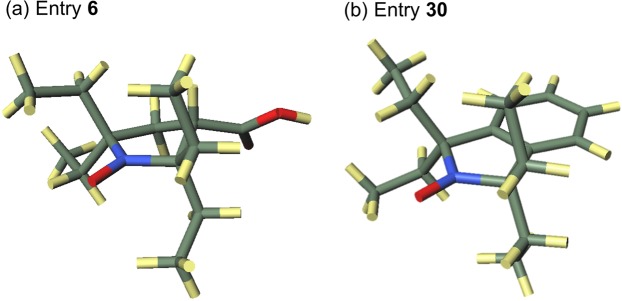
Calculated equilibrium structures for entry **6** and **30**.

The low R^2^ value of 0.403 indicates that the E_s_^c^ and DPSH values cannot be related using a single linear regression. Since the members of Group A (**5**, **8**–**12**, **29**, **39**), Group B (**7**, **15**), and Group C (**6**, **14**, **30**) have different DPSH and equal E_s_^c^ values in each group, they contribute to the loss of correlation and linearity. The reason the E_s_^c^ values were equal for the members of each group is that the parameters used to determine the E_s_^c^ values corresponded to the peripheral substituents, which are the same in each group. For example, the nitroxide radicals belonging to groups A, B, and C have Me, spirocyclohexyl, and ethyl (Et) groups, respectively, around the reaction center in common. However, in the case of molecules that have different skeleton structures, such as a five-membered ring or a six-membered ring and chain, the steric hindrance is expected to increase as the angle Θ increases, even though they have the same substituents. In order to confirm the skeleton effect, we investigated two groups, D and E, that consist of nitroxide radicals with the same skeleton. Group D consists of Entries 5–7, which have an aliphatic five-membered ring skeleton. Group E consists of Entries 8–15, which have a six-membered ring skeleton. For each group, the correlation coefficient and R^2^ value of the linear correlation between E_s_^c^ and DPSH improved compared with those in Fig. 2, with values of −0.991 and 0.982 for Group D and −0.997 and 0.995 for Group E, respectively. The plots for each group are shown in the SI. Both **6** and **30** have four Et groups around the reaction center on the five-membered ring, but their conformations are different (Fig. 3). In the latter, one more Et group is bent in a different direction from the reaction center than in the former, so the steric hindrance around the reaction center is expected to be smaller. While the E_s_^c^ value did not reflect these differences in each group, the DPSH values matched the expected magnitude order of steric hindrance at the reaction center. Therefore, the evaluation of steric hindrance with DPSH and E_s_^c^ are similar when only the substituent type affects the steric hindrance, but DPSH is considered to be more correct when other factors also affect the steric hindrance.

**Figure 4:**
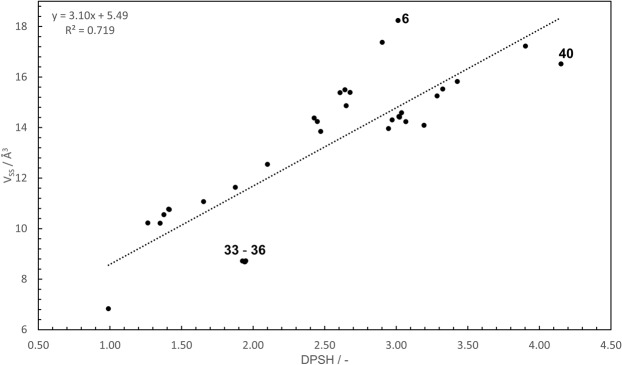
Plots of V_SS_ *versus* DPSH, together with the line of best fit.

The DPSH was also compared with V_SS_, and the results are shown in Fig. 4. The correlation coefficient of 0.848 confirms the presence of a stronger positive correlation between the DPSH and V_SS_ than between the DPSH and E_s_^c^, and the R^2^ value of 0.719 represents moderate linearity. The positive correlation is consistent with an increase in the steric hindrance, which corresponds to an increase in the DPSH and V_SS_. As a result, although the DPSH considers transition state structures and V_SS_ considers equilibrium structures, the evaluations of the steric hindrance with the DPSH and V_SS_ are correlated, so the DPSH is found to be affected by the equilibrium structures of the nitroxide radicals as well as their transition state structures, as V_SS_ is determined by the equilibrium structures. This is in accordance with the fact that the order of magnitude of the DPSH values for some nitroxide radicals can be explained by the equilibrium structural factors of the nitroxide radicals, such as the number of substituents and the bond angle, as mentioned above.

Notably, Fig. 4 shows some discrepancies. More specifically, some data points deviate significantly from the regression line, and the nitroxide radical that had the highest DPSH value (**40**) differed from the radical that had the highest V_SS_ value (**6**). In other words, among the nitroxide radicals evaluated, **40** has the largest steric hindrance as assessed by the DPSH, whereas **6** has the largest steric hindrance as assessed by V_SS_. Three reasons were considered to explain why **40** has a higher steric hindrance than **6** as determined by the DPSH.

First, the calculated structures may have been a local minimum, which would mean that the DPSH value was determined using the incorrect structure. Due to the challenge of performing transition state calculation exhaustively, such a flaw could be possible.

Second, the elements that make up DPSH were considered using the total nuclear and electronic energy instead of the enthalpy from which DPSH is derived from so as to deal with unoptimized structures. The activation energy $\Delta {E}_{{\rm{TSn}}}^{\ddagger }$(n = 1 or 2) in each addition reaction is represented in Eq. 1 by three factors: the energy increase associated with the change from the equilibrium structure to the transition state of the nitroxide radical, $\Delta {E}_{{\rm{N}},{\rm{TSn}}}$; the energy increase associated with the change from the equilibrium structure to the transition state of olefins, $\Delta {E}_{{\rm{O}},{\rm{TSn}}}$; and the interaction energy of the nitroxide radical with the olefin, ${E}_{{\rm{I}},{\rm{TSn}}}$

1 $$\Delta {E}_{{\rm{TSn}}}^{\ddagger }=\Delta {E}_{{\rm{N}},{\rm{TSn}}}+\Delta {E}_{{\rm{O}},{\rm{TSn}}}+{E}_{{\rm{I}},{\rm{TSn}}}$$

**Figure 5:**
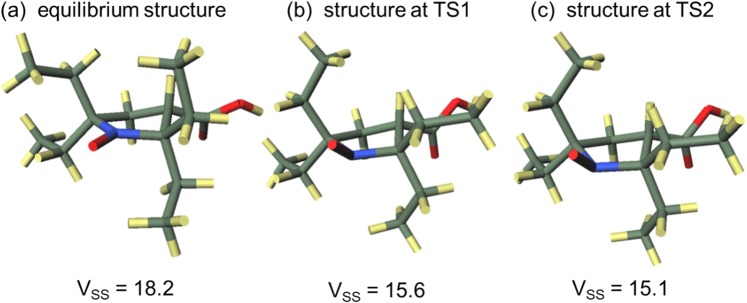
Equilibrium and transition state structures of the nitroxide radical in entry **6**.

**Figure 6:**
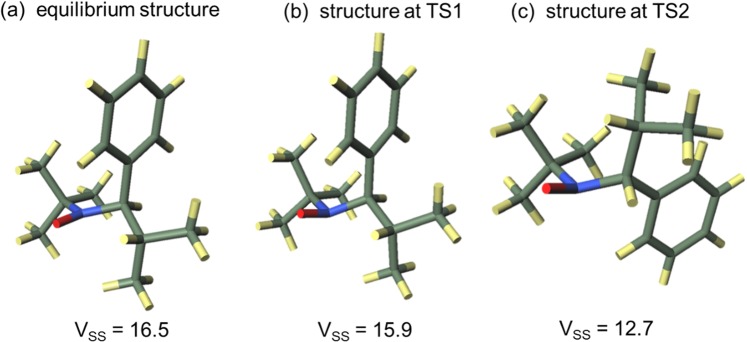
Equilibrium and transition state structures of the nitroxide radical in entry **40**.

Each term was calculated from the energy of the equilibrium structure of the nitroxide radicals and olefins, the energy of the transition state, and the energy for each structure of nitroxide radical and olefin in the transition state, which were obtained by theoretical calculation. Based on Eq. 1, the difference in activation enthalpy ${\Delta }^{2}{H}_{{\rm{TS}}}^{\ddagger }$ in the two addition reactions, i.e., the DPSH value that is not reported relative to Me, is obtained by Eq. 2.

2 $$\begin{array}{ccc}{\Delta }^{2}{H}_{{\rm{TS}}}^{\ddagger } & = & \Delta {H}_{2}^{\ddagger }-\Delta {H}_{1}^{\ddagger }\\  & = & (\Delta {E}_{{\rm{TS}}2}^{\ddagger }-\Delta {E}_{{\rm{TS}}1}^{\ddagger })+{\rm{\delta }}\\  & = & (\Delta {E}_{{\rm{N}},{\rm{TS}}2}+\Delta {E}_{{\rm{O}},{\rm{TS}}2}+{E}_{{\rm{I}},{\rm{TS}}2})-(\Delta {E}_{{\rm{N}},{\rm{TS}}1}+\,\Delta {E}_{{\rm{O}},{\rm{TS}}1}+{E}_{{\rm{I}},{\rm{TS}}1})+{\rm{\delta }}\\  & = & (\Delta {E}_{{\rm{N}},{\rm{TS}}2}-\Delta {E}_{{\rm{N}},{\rm{TS}}1})+(\Delta {E}_{{\rm{O}},{\rm{TS}}2}-\Delta {E}_{{\rm{O}},{\rm{TS}}1})+({E}_{{\rm{I}},{\rm{TS}}2}-{E}_{{\rm{I}},{\rm{TS}}1})+{\rm{\delta }}\\  & = & {\Delta }^{2}{E}_{{\rm{N}},{\rm{TS}}}+{\Delta }^{2}{E}_{{\rm{O}},{\rm{TS}}}+\Delta {E}_{{\rm{I}},{\rm{TS}}}+{\rm{\delta }}\end{array}$$

where ${\rm{\delta }}$ is a term for correcting the total nuclear and electronic energy to enthalpy, which is derived from the zero point energy. In the last line, we define ${\Delta }^{2}{E}_{{\rm{N}},{\rm{TS}}}=\Delta {E}_{{\rm{N}},{\rm{TS}}2}-\Delta {E}_{{\rm{N}},{\rm{TS}}1}$, ${\Delta }^{2}{E}_{{\rm{O}},{\rm{TS}}}=\Delta {E}_{{\rm{O}},{\rm{TS}}2}-\Delta {E}_{{\rm{O}},{\rm{TS}}1}$, and $\Delta {E}_{{\rm{I}},{\rm{TS}}}={E}_{{\rm{I}},{\rm{TS}}2}-{E}_{{\rm{I}},{\rm{TS}}1},$ respectively. The values of the terms in Eq. 2 for **6** and **40** are given in Table 6. Also, Figs. 5 and 6 show the equilibrium and transition state structures of the nitroxide radicals, as well as the V_SS_ value for each structure of **6** and **40**, respectively. The increase in each term in Eq. 2 contributes to the increase of ${\Delta }^{2}{H}_{{\rm{TS}}}^{\ddagger }$. According to $\Delta {E}_{{\rm{I}},{\rm{TS}}}$ in Table 6, the second reason that **40** has a larger DPSH value than **6** is the increase of the repulsive interaction between nitroxide radicals and olefins. Both **6** and **40** have positive $\Delta {E}_{{\rm{I}},{\rm{TS}}}$ values, which indicates that the repulsive interaction is increased in reactions with bulky substituted olefins compared to the repulsive interaction in reactions with non-bulky substituted olefins. The fact that the increase in the repulsive interaction was larger in **40** than in **6**, which contributes to making the DPSH of **40** greater than that of **6**, was shown by the larger $\Delta {E}_{{\rm{I}},{\rm{TS}}}$ value for **40** than that for **6**. In both nitroxide radicals, V_SS_ in the TS2 structure was smaller than that in the TS1 structure, which suggests that the repulsive interaction is also caused by atoms located outside of the virtual sphere for V_SS_. When using V_SS_, it will be necessary to change the radius of the virtual sphere in order to incorporate this interaction.

**Table 6:** The values of the terms of Eq. 2 for entry **6** and **40**.

Entry	6	40
Δ^2^*H*^‡^_TS_/kJ mol^−1^	71.1	97.9
Δ^2^*E*_N,TS_/kJ mol^−1^	27.5	63.6
Δ^2^*E*_O,TS_/kJ mol^−1^	34.0	21.4
Δ*E*_1,TS_/kJ mol^−1^	13.7	15.1
δ/kJ mol^−1^	−4.08	−2.15

The third reason why **40** has a higher steric hindrance than **6** is the structural changes in the nitroxide radicals. As Figs. 5 and 6 show, both **6** and **40** change from their equilibrium structures as the reaction proceeds. More specifically, the bending direction of the ethyl group in **6** changes in both transition states, while the C-N bond rotates in the TS2 of **40**. Moreover, in the transition states, they lose the planar structure around the reaction center observed in the equilibrium structure. The V_SS_ value in each transition state is lowered compared to the equilibrium structures, which indicates that these structural changes occur to reduce the steric repulsion between the nitroxide radicals and olefins. In particular, the bonding rotation in TS2 of **40** occurs to avoid the repulsion between the isopropyl group and olefin. As the positive ${\Delta }^{2}{E}_{{\rm{N}},{\rm{TSn}}}$ values in Table 6 indicate, the energy increase due to these structural changes from equilibrium structures is larger in TS2 than in TS1 for both **6** and **40**. The larger ${\Delta }^{2}{E}_{{\rm{N}},{\rm{TS}}}$ of **40** than that of **6** contributes to making the DPSH of **40** greater than that of **6**. The contribution of ${\Delta }^{2}{E}_{{\rm{N}},{\rm{TS}}}$ to DPSH is predicted to be greatest in terms of the comparison of ${\Delta }^{2}{E}_{{\rm{N}},{\rm{TS}}},{\Delta }^{2}{E}_{{\rm{I}},{\rm{TS}}}$, and ${\rm{\delta }}$. In order for a radical to react with olefins with bulkier substituents, it is necessary to create a space near the reaction center. The DPSH value of radicals that need a large amount of energy to create such a space can be large even if the steric shielding in their equilibrium structures is small. As a result, the DPSH is also influenced by dynamic factors, such as structural changes, associated with the reaction.

When any term of Eq. 2 for one nitroxide radical is especially high or low compared to other nitroxide radicals with similar V_SS_ values, its DPSH value can be significantly different from that predicted by the regression line in Fig. 4. For example, since **33**, whose planar structure breaks down in TS2, is a ring structure with a π-conjugated system linked via one-electron transfer resonance at the N–O bond, the structural change is energetically disadvantageous. Therefore, the contribution of ${\Delta }^{2}{E}_{{\rm{N}},{\rm{TS}}}$ is considered to make the DPSH larger than that predicted from the regression line, but additional calculations like those for **6** and **40**, at least, are necessary to investigate this hypothesis. Additional calculations for other radicals are also necessary to investigate the general trends of factors affecting DPSH values.

## Conclusions

In this work, the steric hindrance in the reaction center of 43 radical species, most of which were nitroxide radicals, was evaluated using the DPSH, a value based on theoretical calculation. While most conventional theoretical methods are static evaluation methods that focus on the equilibrium structures, DPSH represents a dynamic evaluation method that focuses on the activation enthalpy in the model reactions. The obtained values corresponded reasonably well to the parameters of the equilibrium structure of the nitroxide radical, such as bond angles and number of substituents, as well as to previous experimental results for the reactivity parameters of the radicals, such as reduction rate, radical trapping rate, and the ability to oxidize alcohols to carbonyl compounds. Furthermore, despite the use of activation enthalpy rather than geometrical parameters, it was confirmed that the influence of electronic factors was suppressed by using the differences in activation enthalpies for different transition states. The DPSH values calculated for alkyl radicals and phenoxy radicals corresponded to their structure, which suggested that this approach was applicable not only to nitroxide radicals but also to other radical species. Comparison with other methods and transition state calculations showed that the DPSH was affected by the repulsive interaction between molecules and the energy barrier in the change of molecular structure to reduce the repulsive interaction. In the future, DPSH values based on other calculation methods such as CCSD, olefins, and/or model reactions will be investigated. Moreover, the DPSH calculation is expected to be simplified by using methods that can incorporate interaction and structural change factors without undergoing difficult transition state calculations.

## Supplementary information

Supplementary information
