# Supplementary material for: A theoretical, dynamical evaluation method of the steric hindrance in nitroxide radicals using transition states of model reactions
Source: Sci Rep. 2019 Dec 30;9:20339. doi: 10.1038/s41598-019-56342-w (PMC6937270; doi:10.1038/s41598-019-56342-w)
Supplement: Supplementary file 1 — Supplementary information [file 41598_2019_56342_MOESM1_ESM.docx]

Supplementary information on the paper

A theoretical, dynamical evaluation method of the steric hindrance in nitroxide radicals using transition states of model reactions

Yudai Yamazaki, Jun Naganuma, Hiroaki Gotoh*

Department of Chemistry and Life Science, Yokohama National

University, 79-5 Tokiwadai, Hodogaya-ku, Yokohama, Japan

Contents

[1. The information of equilibrium structures 2](#_Toc22835383)

[2. The information of transition state structures 48](#_Toc22835384)

[3. The information of olefins and nitroxide radicals for each transition state 254](#_Toc22835385)

[4. The relationships between E_s_^c^ and DPSH in groups of each skeleton 267](#_Toc22835386)

[5. References 268](#_Toc22835387)

# 1. The information of equilibrium structures

The information for the calculated equilibrium structures of olefins and radicals are described. Coordinates, thermochemical values (zero-point correction, thermal correction to energy, thermal correction to enthalpy, thermal correction to Gibbs free energy, sum of electronic and zero-point energies, sum of electronic and thermal energies, sum of electronic and thermal enthalpies, and sum of electronic and thermal free energies), and S^2^ values were calculated at (U)B3LYP/6-31G* and (U)M06-2X/6-31G* level. The total energy of nuclei and electrons was calculated at (U)B3LYP/6-311+G** level. SOMO energy for nitroxide radicals was calculated at ROB3LYP/6-311+G** level.

2-methylpropene (B3LYP)

C 0.000044 1.459967 0.000000

C -0.000023 0.123198 0.000000

C -0.000023 -0.679023 1.277604

C -0.000023 -0.679023 -1.277604

H 0.000069 2.030909 -0.925178

H 0.000069 2.030909 0.925178

H 0.880070 -1.334708 1.332340

H -0.879823 -1.335100 1.332110

H -0.000245 -0.036457 2.163506

H 0.880070 -1.334708 -1.332340

H -0.000245 -0.036457 -2.163506

H -0.879823 -1.335100 -1.332110

Zero-point correction= 0.108507 Hartree/Particle

Thermal correction to Energy= 0.113812

Thermal correction to Enthalpy= 0.114756

Thermal correction to Gibbs Free Energy= 0.081195

Sum of electronic and zero-point Energies= -157.118780

Sum of electronic and thermal Energies= -157.113475

Sum of electronic and thermal Enthalpies= -157.112531

Sum of electronic and thermal Free Energies= -157.146092

The total energy of nuclei and electrons= -157.274970544

2-methylpropene (M06-2X)

C 0.000022 1.456457 0.000000

C -0.000010 0.124462 0.000000

C -0.000010 -0.678222 1.272981

C -0.000010 -0.678222 -1.272981

H 0.000032 2.024871 -0.925780

H 0.000032 2.024871 0.925780

H 0.879986 -1.331120 1.319886

H -0.879936 -1.331214 1.319839

H -0.000062 -0.035962 2.156903

H 0.879986 -1.331120 -1.319886

H -0.000062 -0.035962 -2.156903

H -0.879936 -1.331214 -1.319839

Zero-point correction= 0.109591 (Hartree/Particle)

Thermal correction to Energy= 0.114853

Thermal correction to Enthalpy= 0.115798

Thermal correction to Gibbs Free Energy= 0.082343

Sum of electronic and zero-point Energies= -157.024001

Sum of electronic and thermal Energies= -157.018739

Sum of electronic and thermal Enthalpies= -157.017795

Sum of electronic and thermal Free Energies= -157.051249

2,​2,​4,​4-​tetramethyl-​3-​methylenepentane (B3LYP)

C -0.000035 1.993133 -0.000016

C -0.000005 0.651432 -0.000018

C 1.369482 -0.095795 0.003094

C 2.524963 0.839767 -0.431019

C 1.713014 -0.572445 1.439685

C 1.427404 -1.304845 -0.961346

C -1.369478 -0.095813 -0.003092

C -1.427433 -1.304771 0.961464

C -2.524960 0.839809 0.430890

C -1.712955 -0.572599 -1.439651

H -0.910195 2.578066 0.039695

H 0.910092 2.578116 -0.039712

H 3.452859 0.260722 -0.500007

H 2.703363 1.644121 0.289613

H 2.335025 1.291115 -1.410821

H 2.724281 -0.998213 1.457870

H 1.028860 -1.337377 1.811344

H 1.689275 0.270418 2.139287

H 2.424786 -1.757945 -0.911673

H 1.258166 -0.994672 -1.998426

H 0.707801 -2.089829 -0.719874

H -2.424788 -1.757923 0.911741

H -1.258311 -0.994490 1.998530

H -0.707770 -2.089746 0.720137

H -3.452872 0.260791 0.499895

H -2.703312 1.644102 -0.289822

H -2.335048 1.291245 1.410656

H -2.724205 -0.998408 -1.457824

H -1.028760 -1.337537 -1.811225

H -1.689229 0.270204 -2.139326

Zero-point correction= 0.280706 Hartree/Particle

Thermal correction to Energy= 0.292969

Thermal correction to Enthalpy= 0.293913

Thermal correction to Gibbs Free Energy= 0.244564

Sum of electronic and zero-point Energies= -392.804896

Sum of electronic and thermal Energies= -392.792633

Sum of electronic and thermal Enthalpies= -392.791689

Sum of electronic and thermal Free Energies= -392.841038

The total energy of nuclei and electrons= -393.195762908

2,​2,​4,​4-​tetramethyl-​3-​methylenepentane (M06-2X)

C -0.000003 1.990896 0.000002

C -0.000001 0.653046 0.000005

C -1.354652 -0.095843 -0.001345

C -2.507149 0.835470 0.417774

C -1.693512 -0.585725 -1.424848

C -1.401275 -1.287148 0.971338

C 1.354652 -0.095842 0.001348

C 1.401254 -1.287170 -0.971307

C 2.507141 0.835460 -0.417818

C 1.693543 -0.585690 1.424856

H 0.911531 2.573157 -0.041720

H -0.911539 2.573154 0.041723

H -3.429905 0.251638 0.497402

H -2.686260 1.623709 -0.319278

H -2.313173 1.303421 1.388111

H -2.713951 -0.986502 -1.439638

H -1.025760 -1.372804 -1.777347

H -1.644375 0.247373 -2.133643

H -2.398155 -1.740177 0.932551

H -1.221929 -0.960814 2.001318

H -0.681253 -2.071373 0.730031

H 2.398144 -1.740180 -0.932553

H 1.221855 -0.960864 -2.001287

H 0.681258 -2.071403 -0.729949

H 3.429897 0.251628 -0.497435

H 2.686258 1.623727 0.319204

H 2.313153 1.303375 -1.388170

H 2.713992 -0.986444 1.439636

H 1.025816 -1.372778 1.777379

H 1.644400 0.247420 2.133636

Zero-point correction= 0.283244 (Hartree/Particle)

Thermal correction to Energy= 0.295294

Thermal correction to Enthalpy= 0.296238

Thermal correction to Gibbs Free Energy= 0.247234

Sum of electronic and zero-point Energies= -392.598318

Sum of electronic and thermal Energies= -392.586268

Sum of electronic and thermal Enthalpies= -392.585324

Sum of electronic and thermal Free Energies= -392.634329

Entry **1** (UB3LYP)

C 0.000000 0.000000 0.000000

H 0.000000 1.082752 0.000000

H 0.937691 -0.541376 0.000000

H -0.937691 -0.541376 0.000000

Zero-point correction= 0.029834 Hartree/Particle

Thermal correction to Energy= 0.032941

Thermal correction to Enthalpy= 0.033885

Thermal correction to Gibbs Free Energy= 0.011699

Sum of electronic and zero-point Energies= -39.808458

Sum of electronic and thermal Energies= -39.805351

Sum of electronic and thermal Enthalpies= -39.804407

Sum of electronic and thermal Free Energies= -39.826593

Entry **1** (UM06-2X)

C 0.000000 0.000000 0.000000

H 0.000000 1.079719 0.000000

H 0.935064 -0.539860 0.000000

H -0.935064 -0.539860 0.000000

Zero-point correction= 0.029985 (Hartree/Particle)

Thermal correction to Energy= 0.033147

Thermal correction to Enthalpy= 0.034091

Thermal correction to Gibbs Free Energy= 0.011817

Sum of electronic and zero-point Energies= -39.774245

Sum of electronic and thermal Energies= -39.771083

Sum of electronic and thermal Enthalpies= -39.770139

Sum of electronic and thermal Free Energies= -39.792413

Entry **2**

C -0.694843 0.000004 -0.000471

H -1.109003 0.887444 -0.494199

H -1.108995 -0.887396 -0.494285

H -1.093591 -0.000062 1.029571

C 0.795508 0.000001 -0.023744

H 1.353786 -0.927675 0.052094

H 1.353810 0.927659 0.052107

Zero-point correction= 0.059657 Hartree/Particle

Thermal correction to Energy= 0.063622

Thermal correction to Enthalpy= 0.064566

Thermal correction to Gibbs Free Energy= 0.035505

Sum of electronic and zero-point Energies= -79.098210

Sum of electronic and thermal Energies= -79.094245

Sum of electronic and thermal Enthalpies= -79.093301

Sum of electronic and thermal Free Energies= -79.122362

Entry **3**

C -1.301222 -0.197649 0.003723

H -1.298238 -1.080248 -0.651755

H -1.527254 -0.572268 1.019574

H -2.141650 0.440840 -0.291549

C 0.000000 0.531615 -0.052977

H 0.000000 1.605454 0.120643

C 1.301222 -0.197650 0.003723

H 2.141647 0.440830 -0.291579

H 1.527269 -0.572239 1.019581

H 1.298226 -1.080266 -0.651731

Zero-point correction= 0.088604 Hartree/Particle

Thermal correction to Energy= 0.093743

Thermal correction to Enthalpy= 0.094688

Thermal correction to Gibbs Free Energy= 0.061404

Sum of electronic and zero-point Energies= -118.389548

Sum of electronic and thermal Energies= -118.384409

Sum of electronic and thermal Enthalpies= -118.383465

Sum of electronic and thermal Free Energies= -118.416749

Entry **4**

C 0.000128 -0.000108 -0.143216

C 1.425177 -0.432954 0.014592

H 2.120387 0.281805 -0.444233

H 1.721671 -0.516869 1.077791

H 1.602951 -1.418328 -0.434807

C -0.337586 1.450424 0.014574

H -1.304515 1.694941 -0.443629

H -0.412210 1.748918 1.077866

H 0.426450 2.097319 -0.435246

C -1.087598 -1.017466 0.014597

H -2.028540 -0.680518 -0.439031

H -0.814748 -1.978555 -0.439848

H -1.312177 -1.228089 1.077852

Zero-point correction= 0.117280 Hartree/Particle

Thermal correction to Energy= 0.123661

Thermal correction to Enthalpy= 0.124605

Thermal correction to Gibbs Free Energy= 0.087768

Sum of electronic and zero-point Energies= -157.681042

Sum of electronic and thermal Energies= -157.674661

Sum of electronic and thermal Enthalpies= -157.673717

Sum of electronic and thermal Free Energies= -157.710554

Entry **5**

The coordinates and the thermochemical values were quoted from the reference. (1)

Entry **6**

C -0.895578 -0.577938 0.252319

N 0.581574 -0.748158 0.342388

C 1.405982 0.409119 -0.117473

C 0.311952 1.454496 -0.425435

C -0.964319 0.644392 -0.720146

C 2.341525 0.831810 1.035452

C 3.257002 2.020229 0.721233

C 2.173817 0.029685 -1.414075

C 3.246660 -1.057165 -1.283438

C -1.482007 -0.209122 1.648129

C -1.204128 -1.195048 2.789051

C -1.550030 -1.867255 -0.287455

C -1.029743 -2.392096 -1.629098

C -2.224898 1.473231 -0.593355

O -2.320421 2.554570 -0.056124

O -3.288981 0.853243 -1.167551

O 1.101245 -1.801931 0.847864

H 0.137270 2.107141 0.434938

H 0.579676 2.098052 -1.268492

H -0.937071 0.262304 -1.744939

H 1.711747 1.077819 1.900279

H 2.936034 -0.038897 1.330145

H 2.686441 2.905550 0.415754

H 3.969598 1.789308 -0.077868

H 3.837875 2.294767 1.608635

H 1.434488 -0.282198 -2.163426

H 2.629188 0.948410 -1.806151

H 2.831705 -1.978289 -0.867601

H 3.675366 -1.279144 -2.267563

H 4.067993 -0.740033 -0.631399

H -1.106048 0.780043 1.935017

H -2.568109 -0.096700 1.526013

H -0.132660 -1.360670 2.924879

H -1.615207 -0.796260 3.723578

H -1.670413 -2.169892 2.615388

H -1.399407 -2.647687 0.463949

H -2.629172 -1.684068 -0.351497

H -1.221819 -1.702471 -2.458929

H 0.045086 -2.591664 -1.583039

H -1.531540 -3.334370 -1.875497

H -4.054194 1.442975 -1.026657

Zero-point correction= 0.363489 Hartree/Particle

Thermal correction to Energy= 0.383116

Thermal correction to Enthalpy= 0.384060

Thermal correction to Gibbs Free Energy= 0.315445

Sum of electronic and zero-point Energies= -789.857679

Sum of electronic and thermal Energies= -789.838052

Sum of electronic and thermal Enthalpies= -789.837108

Sum of electronic and thermal Free Energies= -789.905722

The total energy of nuclei and electrons= -790.452609258

Entry **7**

C -1.068218 -0.418199 0.128476

N 0.241769 -0.736158 0.758274

C 1.451053 -0.264247 0.031559

C 0.834579 0.518750 -1.151218

C -0.678016 0.737449 -0.850228

C 2.292530 0.605272 0.996274

C 3.656580 0.997267 0.408361

C 4.456677 -0.231274 -0.045649

C 3.638300 -1.085102 -1.024020

C 2.283130 -1.485533 -0.421622

C -2.079644 -0.082694 1.244914

C -3.535548 0.010712 0.760040

C -3.965573 -1.259404 0.014548

C -3.006976 -1.556968 -1.145786

C -1.554593 -1.665798 -0.658129

C -0.933721 2.124834 -0.290999

O -0.249623 2.717606 0.516071

O -2.055455 2.679776 -0.818731

O 0.325136 -1.589633 1.703489

H 1.341804 1.472257 -1.315131

H 0.928523 -0.060968 -2.075132

H -1.267119 0.652817 -1.765772

H 1.717198 1.494538 1.268492

H 2.439031 0.014339 1.909411

H 3.513965 1.672484 -0.448586

H 4.221537 1.569586 1.154774

H 5.403290 0.076505 -0.507832

H 4.717532 -0.839650 0.832626

H 3.482247 -0.521561 -1.956218

H 4.194298 -1.989395 -1.302189

H 2.438601 -2.117362 0.460751

H 1.703411 -2.079959 -1.140049

H -1.986069 -0.887017 1.983663

H -1.773104 0.838305 1.753474

H -4.187632 0.182572 1.625650

H -3.658935 0.879110 0.100204

H -3.965706 -2.110230 0.711152

H -4.992564 -1.154804 -0.357169

H -3.288148 -2.489703 -1.650854

H -3.093880 -0.761328 -1.900536

H -1.456346 -2.519242 0.024070

H -0.880135 -1.855642 -1.502952

H -2.147993 3.555085 -0.395516

Zero-point correction= 0.382673 Hartree/Particle

Thermal correction to Energy= 0.399919

Thermal correction to Enthalpy= 0.400864

Thermal correction to Gibbs Free Energy= 0.337718

Sum of electronic and zero-point Energies= -866.061869

Sum of electronic and thermal Energies= -866.044623

Sum of electronic and thermal Enthalpies= -866.043678

Sum of electronic and thermal Free Energies= -866.106824

Entry **8 - 9**

The coordinates and the thermochemical values were quoted from the reference. (1)

Entry **10**

The hydroxyl group is in the axial position.

C -1.321657 -0.334227 -0.062301

N 0.022812 -0.999568 -0.072596

C 1.338767 -0.275994 -0.057064

C 1.233954 1.041075 -0.856839

C -0.036249 1.858664 -0.636988

C -1.267372 0.977360 -0.873430

O 0.046387 -2.208067 0.364765

C 2.361446 -1.195842 -0.747925

C 1.777654 -0.040447 1.404205

C -2.307757 -1.300624 -0.742112

C -1.766298 -0.102936 1.398135

O 0.012897 2.440627 0.669779

H 1.280118 0.807213 -1.928321

H 2.108740 1.660075 -0.627958

H -0.049254 2.667437 -1.386149

H -1.286380 0.733224 -1.944207

H -2.186327 1.546948 -0.675287

H 3.331038 -0.688689 -0.804618

H 2.039049 -1.442631 -1.765373

H 2.479306 -2.127029 -0.191122

H 2.811724 0.323131 1.424414

H 1.730902 -0.986503 1.951308

H 1.151680 0.701852 1.900957

H -3.298308 -0.835457 -0.800215

H -2.384512 -2.231551 -0.177885

H -1.977733 -1.541774 -1.758306

H -2.809914 0.233946 1.421365

H -1.150386 0.650298 1.892056

H -1.694531 -1.044444 1.949974

H -0.804082 2.948285 0.797094

Zero-point correction= 0.267931 Hartree/Particle

Thermal correction to Energy= 0.280937

Thermal correction to Enthalpy= 0.281881

Thermal correction to Gibbs Free Energy= 0.230188

Sum of electronic and zero-point Energies= -558.659234

Sum of electronic and thermal Energies= -558.646228

Sum of electronic and thermal Enthalpies= -558.645284

Sum of electronic and thermal Free Energies= -558.696977

Entry **11 – 18**

The coordinates and the thermochemical values were quoted from the reference. (1)

Entry **19**

C -0.274996 -0.045219 -0.388290

C -0.812347 1.236297 -1.074670

C -2.089587 1.731071 -0.425000

C -3.206927 0.711673 -0.487820

C -2.815400 -0.595307 0.245171

N -1.413064 -0.999098 -0.128948

C 0.729643 -0.776714 -1.308031

C 2.059653 -0.027836 -1.445574

C 0.427061 0.279836 0.957435

C 1.793233 0.965748 0.780529

N 2.650558 0.224131 -0.133614

O -2.189861 2.814502 0.117677

O -1.070563 -2.174914 0.258215

C 3.941559 -0.188821 0.118264

O 4.607153 -0.776474 -0.727704

C 4.520319 0.106720 1.497401

C -2.904260 -0.405230 1.773188

C -3.748553 -1.733827 -0.193766

H -1.027157 1.009026 -2.127484

H -0.064475 2.031271 -1.046046

H -3.410157 0.483883 -1.542889

H -4.114891 1.127218 -0.042587

H 0.279630 -0.921727 -2.297998

H 0.914492 -1.765916 -0.883100

H 1.921366 0.929278 -1.972141

H 2.777420 -0.618463 -2.014959

H 0.565288 -0.669265 1.485923

H -0.205973 0.924054 1.578011

H 2.273841 1.053296 1.755151

H 1.656794 1.991987 0.403758

H 4.561665 1.182370 1.703230

H 5.533995 -0.294662 1.512924

H 3.939950 -0.366994 2.297365

H -2.483583 -1.280318 2.275973

H -2.371752 0.489527 2.109926

H -3.952297 -0.299546 2.074153

H -3.513971 -2.653197 0.345372

H -4.787525 -1.454411 0.011287

H -3.645337 -1.927902 -1.266454

Zero-point correction= 0.337107 Hartree/Particle

Thermal correction to Energy= 0.354899

Thermal correction to Enthalpy= 0.355843

Thermal correction to Gibbs Free Energy= 0.291046

Sum of electronic and zero-point Energies= -842.816047

Sum of electronic and thermal Energies= -842.798254

Sum of electronic and thermal Enthalpies= -842.797310

Sum of electronic and thermal Free Energies= -842.862107

Entry **20** (UB3LYP)

The coordinates and the thermochemical values were quoted from the reference. (1)

Entry **20** (UM06-2X)

C -0.600389 1.239611 -0.277988

N -1.369630 0.000002 -0.502684

C -0.600388 -1.239608 -0.277999

C 0.619200 -1.245942 -1.202467

C 1.474118 0.000004 -0.922153

C 0.619200 1.245953 -1.202456

H -1.287990 2.058990 -0.499717

O -2.589296 0.000000 -0.132011

H -1.287990 -2.058986 -0.499736

H 0.293594 -1.258778 -2.248842

H 1.199344 -2.159002 -1.021819

H 2.353290 0.000007 -1.576083

H 0.293593 1.258798 -2.248830

H 1.199343 2.159011 -1.021800

C -0.163092 1.251739 1.196046

H 0.409903 2.163418 1.405577

H -1.057419 1.259137 1.829163

C 0.687810 -0.000007 1.472894

C -0.163091 -1.251750 1.196035

H 0.409903 -2.163431 1.405558

H -1.057419 -1.259154 1.829152

C 1.918277 -0.000002 0.550346

H 2.536999 0.883553 0.754129

H 2.536999 -0.883559 0.754121

H 1.009748 -0.000012 2.520451

Zero-point correction= 0.227110 (Hartree/Particle)

Thermal correction to Energy= 0.234807

Thermal correction to Enthalpy= 0.235751

Thermal correction to Gibbs Free Energy= 0.194514

Sum of electronic and zero-point Energies= -480.874273

Sum of electronic and thermal Energies= -480.866575

Sum of electronic and thermal Enthalpies= -480.865631

Sum of electronic and thermal Free Energies= -480.906869

Entry **21** (UB3LYP)

The coordinates and the thermochemical values were quoted from the reference. (1)

Entry **21** (UM06-2X)

N -0.918903 0.829590 -0.473514

C 0.450021 1.380434 -0.539535

C 1.305441 0.503049 -1.455162

C 1.313441 -0.930930 -0.908631

C -0.131405 -1.445654 -0.894083

C -1.008471 -0.574229 0.017389

C 1.014673 1.390109 0.889660

C -0.429505 -0.577608 1.445509

C 1.878285 -0.938103 0.520841

C 1.014004 -0.048948 1.430403

O -1.834762 1.641493 -0.116846

C -2.459946 -1.019645 -0.007984

H 0.335954 2.392985 -0.933050

H 2.323532 0.909084 -1.492721

H 0.899871 0.522321 -2.473214

H 1.925864 -1.570504 -1.554174

H -0.168014 -2.478496 -0.524749

H -0.550457 -1.441576 -1.908190

H 0.387939 2.038655 1.512020

H 2.031155 1.802610 0.883658

H -0.462078 -1.598865 1.847185

H -1.064499 0.054152 2.078673

H 2.913605 -0.573426 0.514985

H 1.897564 -1.964661 0.909145

H 1.417588 -0.058404 2.449327

H -3.068937 -0.389438 0.641850

H -2.525374 -2.058204 0.331698

H -2.862524 -0.956170 -1.023522

Zero-point correction= 0.255114 (Hartree/Particle)

Thermal correction to Energy= 0.264277

Thermal correction to Enthalpy= 0.265221

Thermal correction to Gibbs Free Energy= 0.221107

Sum of electronic and zero-point Energies= -520.146152

Sum of electronic and thermal Energies= -520.136989

Sum of electronic and thermal Enthalpies= -520.136044

Sum of electronic and thermal Free Energies= -520.180159

Entry **22** (UB3LYP)

The coordinates and the thermochemical values were quoted from the reference. (1)

Entry **22** (UM06-2X)

C 0.000010 1.198130 1.395383

C 1.251089 0.317362 1.259725

C 1.262654 -0.371987 -0.117911

N -0.000011 -1.161344 -0.208999

C -1.262660 -0.371966 -0.117912

C -1.239506 0.691353 -1.224027

C 0.000014 1.580975 -1.087339

C 0.000018 2.264178 0.288305

C -1.251083 0.317382 1.259725

C 1.239519 0.691332 -1.224026

O -0.000024 -2.311922 0.340180

C 2.445075 -1.312548 -0.276005

C -2.445095 -1.312510 -0.276006

H 0.000014 1.684170 2.377896

H 1.263417 -0.458247 2.035741

H 2.162342 0.919740 1.370756

H -2.155846 1.290724 -1.148276

H -1.249419 0.194301 -2.202325

H 0.000021 2.338172 -1.879960

H 0.884090 2.907490 0.385927

H -0.884044 2.907504 0.385927

H -2.162327 0.919774 1.370757

H -1.263423 -0.458227 2.035740

H 1.249426 0.194280 -2.202323

H 2.155869 1.290688 -1.148273

H 2.386511 -1.839907 -1.233056

H 2.460824 -2.054740 0.523239

H 3.373345 -0.733305 -0.249936

H -3.373356 -0.733254 -0.249933

H -2.460851 -2.054703 0.523237

H -2.386540 -1.839868 -1.233058

Zero-point correction= 0.282292 (Hartree/Particle)

Thermal correction to Energy= 0.293058

Thermal correction to Enthalpy= 0.294002

Thermal correction to Gibbs Free Energy= 0.246769

Sum of electronic and zero-point Energies= -559.418299

Sum of electronic and thermal Energies= -559.407533

Sum of electronic and thermal Enthalpies= -559.406589

Sum of electronic and thermal Free Energies= -559.453822

Entry **23** - **29**

The coordinates and the thermochemical values were quoted from the reference. (1)

Entry **30**

C 2.505971 0.972979 -0.163556

C 1.269755 0.338028 -0.054052

C 0.079449 1.070791 -0.162208

C 0.126926 2.444418 -0.405944

C 1.366104 3.081637 -0.511683

C 2.550613 2.351144 -0.387274

C 1.011466 -1.135646 0.163773

C -1.147534 0.185588 -0.020126

N -0.480272 -1.147294 0.162775

O -1.126394 -2.205190 0.469048

C 1.554207 -2.047220 -0.971059

C 1.558799 -1.683692 1.509033

C -1.996876 0.466762 1.249441

C -1.994566 0.201757 -1.322424

C 1.120466 -0.951857 2.778234

C 1.127388 -1.695821 -2.397265

C -2.875351 1.722136 1.227747

C -3.196908 -0.748664 -1.362179

H 3.428874 0.404216 -0.079780

H -0.786216 3.020211 -0.522331

H 1.406513 4.151723 -0.696444

H 3.509987 2.854393 -0.471819

H 2.649914 -2.038573 -0.902718

H 1.231971 -3.068783 -0.736610

H 1.252917 -2.733917 1.573567

H 2.654287 -1.675540 1.437870

H -2.617809 -0.420661 1.412939

H -1.312570 0.526958 2.102334

H -1.322637 -0.022490 -2.157973

H -2.333759 1.233540 -1.481385

H 1.624564 -1.381307 3.651368

H 1.367102 0.114905 2.741919

H 0.043049 -1.053551 2.938180

H 0.044751 -1.795313 -2.523436

H 1.413045 -0.674170 -2.670432

H 1.604843 -2.377426 -3.110211

H -2.283555 2.637418 1.121774

H -3.615629 1.700810 0.421029

H -3.425401 1.801103 2.172354

H -2.889322 -1.782591 -1.189132

H -3.945718 -0.494790 -0.604083

H -3.685591 -0.687635 -2.341533

Zero-point correction= 0.371966 Hartree/Particle

Thermal correction to Energy= 0.390939

Thermal correction to Enthalpy= 0.391883

Thermal correction to Gibbs Free Energy= 0.325747

Sum of electronic and zero-point Energies= -753.712852

Sum of electronic and thermal Energies= -753.693879

Sum of electronic and thermal Enthalpies= -753.692935

Sum of electronic and thermal Free Energies= -753.759070

Entry **31**

The coordinates and the thermochemical values were quoted from the reference. (1)

Entry **32**

C 3.724663 -0.659595 -0.156092

C 2.324414 -0.643908 -0.242429

C 1.741648 0.140407 -1.262783

C 2.532568 0.825419 -2.197108

C 3.916196 0.771067 -2.095188

C 4.516084 0.038023 -1.064411

C 1.445800 -1.446696 0.624430

N 0.341464 0.219229 -1.357669

C -0.506556 0.024040 -0.121344

O -0.193429 0.765021 -2.384001

C -0.408387 1.338706 0.696011

C -0.204418 1.338650 2.080559

C -0.142266 2.540009 2.792135

C -0.277824 3.758412 2.130006

C -0.480004 3.768011 0.747688

C -0.547150 2.571250 0.037349

C -1.942263 -0.275612 -0.588395

C -2.156475 -1.284238 -1.540777

C -3.444964 -1.624239 -1.940424

C -4.549417 -0.969349 -1.387024

C -4.347490 0.026392 -0.435147

C -3.050984 0.372942 -0.039445

C 0.062048 -1.171437 0.659420

C 1.939195 -2.525678 1.379098

C -0.775357 -1.956966 1.459158

C 1.098738 -3.293587 2.177298

C -0.265981 -3.006618 2.221866

H 4.204082 -1.221318 0.639083

H 2.035749 1.393928 -2.973282

H 4.529404 1.313539 -2.809285

H 5.597752 0.010063 -0.969894

H -0.092022 0.400961 2.613036

H 0.017889 2.516289 3.866912

H -0.225194 4.692505 2.683008

H -0.585654 4.711166 0.217957

H -0.702237 2.585822 -1.036572

H -1.305930 -1.799568 -1.976018

H -3.588453 -2.401708 -2.686132

H -5.556091 -1.235622 -1.698162

H -5.196096 0.543604 0.004797

H -2.911358 1.153413 0.700064

H 2.991917 -2.782185 1.321769

H -1.838650 -1.744913 1.483537

H 1.506252 -4.121057 2.751616

H -0.935087 -3.602111 2.836556

Zero-point correction= 0.359429 Hartree/Particle

Thermal correction to Energy= 0.379528

Thermal correction to Enthalpy= 0.380472

Thermal correction to Gibbs Free Energy= 0.309339

Sum of electronic and zero-point Energies= -1093.057629

Sum of electronic and thermal Energies= -1093.037530

Sum of electronic and thermal Enthalpies= -1093.036586

Sum of electronic and thermal Free Energies= -1093.107719

Entry **33**

The coordinates and the thermochemical values were quoted from the reference. (1)

Entry **34**

The coordinates and the thermochemical values were quoted from the reference. (1)

Annihilation of the first spin contaminant:

S^2^ value before annihilation 0.7617, after 0.7501

SOMO energy= -2.7538 eV

Entry **35**

The coordinates and the thermochemical values were quoted from the reference. (1)

Annihilation of the first spin contaminant:

S^2^ value before annihilation 0.7620, after 0.7501

SOMO energy= -2.6150 eV

Entry **36**

C 2.686215 -1.021175 0.617116

C 1.427652 -0.432319 0.598185

C 1.258916 0.830269 0.005671

C 2.358158 1.512219 -0.540942

C 3.613476 0.922106 -0.525066

C 3.762381 -0.340963 0.049503

N 0.000175 1.494982 0.000347

O 0.000046 2.777728 -0.000439

C -1.258917 0.829901 -0.005393

C -1.427497 -0.432798 -0.597525

C -2.686206 -1.021426 -0.616676

C -3.762383 -0.340938 -0.049493

C -3.613554 0.922176 0.524888

C -2.358114 1.512158 0.540853

N 5.090859 -0.967682 0.066286

O 5.187500 -2.084722 0.574532

O 6.024146 -0.337367 -0.429839

N -5.090999 -0.967607 -0.066514

O -6.024171 -0.337321 0.429785

O -5.187659 -2.084450 -0.575127

H 2.847561 -1.988912 1.075376

H 0.591613 -0.938301 1.066244

H 2.206168 2.495810 -0.967466

H 4.476000 1.419897 -0.950441

H -0.591461 -0.939238 -1.065046

H -2.847598 -1.989270 -1.074699

H -4.476101 1.420152 0.949984

H -2.206080 2.495801 0.967183

Zero-point correction= 0.195654 Hartree/Particle

Thermal correction to Energy= 0.211510

Thermal correction to Enthalpy= 0.212455

Thermal correction to Gibbs Free Energy= 0.149300

Sum of electronic and zero-point Energies= -1002.007885

Sum of electronic and thermal Energies= -1001.992028

Sum of electronic and thermal Enthalpies= -1001.991084

Sum of electronic and thermal Free Energies= -1002.054239

Annihilation of the first spin contaminant:

S^2^ value before annihilation 0.7620, after 0.7501

SOMO energy= -3.6005 eV

Entry **37** – **39**

The coordinates and the thermochemical values were quoted from the reference. (1)

Entry **40**

C 1.957900 -0.212985 1.225203

C 1.161637 0.059197 0.105450

C 3.203762 -0.828949 1.086975

C 3.668317 -1.185573 -0.178907

C 1.637448 -0.303916 -1.163628

C 2.879753 -0.922194 -1.302077

C -0.187850 0.753182 0.272814

N -1.275307 -0.026935 -0.374585

C -0.204377 2.213224 -0.257920

C -1.581428 2.863513 -0.059564

C 0.889458 3.052105 0.419497

C -1.965168 -1.210479 0.247919

O -1.398533 0.091183 -1.648969

C -1.821511 -1.203904 1.775315

C -1.353158 -2.495715 -0.343048

C -3.451690 -1.104592 -0.137046

H 1.603685 0.065856 2.215643

H 3.807395 -1.030221 1.968228

H 4.636195 -1.666988 -0.290883

H 1.017696 -0.115707 -2.034796

H 3.233441 -1.200507 -2.291538

H -0.419847 0.793051 1.340709

H 0.001559 2.167690 -1.332856

H -1.856742 2.890881 1.003689

H -2.360342 2.326350 -0.605891

H -1.566892 3.898229 -0.420982

H 1.889636 2.643649 0.248435

H 0.730702 3.111610 1.504821

H 0.872315 4.076088 0.029553

H -2.228359 -0.288778 2.220025

H -0.781374 -1.322548 2.094703

H -2.387945 -2.047869 2.183111

H -0.302591 -2.601490 -0.054944

H -1.412552 -2.466226 -1.434163

H -1.900673 -3.374312 0.016599

H -3.556663 -1.042142 -1.222072

H -3.905651 -0.211204 0.306534

H -3.996137 -1.983819 0.224019

Zero-point correction= 0.336454 Hartree/Particle

Thermal correction to Energy= 0.353720

Thermal correction to Enthalpy= 0.354664

Thermal correction to Gibbs Free Energy= 0.291160

Sum of electronic and zero-point Energies= -676.322372

Sum of electronic and thermal Energies= -676.305107

Sum of electronic and thermal Enthalpies= -676.304162

Sum of electronic and thermal Free Energies= -676.367667

The total energy of nuclei and electrons= -676.842687099

Entry **41**

C -0.290185 -1.239649 0.000000

C -1.047973 0.000000 0.000000

C -0.290185 1.239648 0.000000

C 1.088014 1.225724 0.000000

C 1.785391 0.000000 0.000000

C 1.088014 -1.225723 0.000000

O -2.305751 0.000000 0.000000

H -0.858118 -2.165076 0.000000

H -0.858118 2.165075 0.000000

H 1.645992 2.158261 0.000000

H 2.871809 0.000000 0.000000

H 1.645992 -2.158260 0.000000

Zero-point correction= 0.091785 Hartree/Particle

Thermal correction to Energy= 0.097052

Thermal correction to Enthalpy= 0.097996

Thermal correction to Gibbs Free Energy= 0.062256

Sum of electronic and zero-point Energies= -306.735856

Sum of electronic and thermal Energies= -306.730590

Sum of electronic and thermal Enthalpies= -306.729646

Sum of electronic and thermal Free Energies= -306.765386

Entry **42**

C 1.313330 0.000591 -0.013521

C 0.591582 -1.218991 -0.011957

C -0.783900 -1.235386 -0.002096

C -1.548021 0.000003 0.003708

C -0.784338 1.235186 -0.002140

C 0.591680 1.219208 -0.011972

H 1.146518 -2.154712 -0.019715

H -1.346352 -2.164248 -0.002445

O -2.804824 -0.000225 0.011559

H -1.346452 2.164244 -0.002510

H 1.145451 2.155663 -0.019686

C 2.817975 -0.000239 0.013853

H 3.230300 0.894998 -0.463233

H 3.229775 -0.881461 -0.488816

H 3.189505 -0.014917 1.048682

Zero-point correction= 0.119372 Hartree/Particle

Thermal correction to Energy= 0.126524

Thermal correction to Enthalpy= 0.127468

Thermal correction to Gibbs Free Energy= 0.086325

Sum of electronic and zero-point Energies= -346.028412

Sum of electronic and thermal Energies= -346.021260

Sum of electronic and thermal Enthalpies= -346.020316

Sum of electronic and thermal Free Energies= -346.061459

Entry **43**

C 0.000008 2.259492 -0.014282

C -1.218394 1.548434 -0.012835

C -1.273637 0.168399 -0.001844

C 0.000001 -0.578378 0.007069

C 1.273635 0.168394 -0.001846

C 1.218398 1.548436 -0.012826

C -0.000002 3.764931 0.015139

C 2.606173 -0.596365 -0.001501

C 3.817406 0.358380 -0.014624

C 2.700371 -1.493982 -1.261989

C 2.711385 -1.473036 1.272755

C -2.606175 -0.596363 -0.001500

C -3.817409 0.358379 -0.014644

C -2.711398 -1.473020 1.272764

C -2.700360 -1.493993 -1.261979

O -0.000001 -1.835747 0.019539

H -2.135025 2.128482 -0.021159

H 2.135037 2.128471 -0.021139

H 0.888338 4.177899 -0.474304

H 0.000145 4.135657 1.050471

H -0.888487 4.177881 -0.474052

H 4.740291 -0.231796 -0.013972

H 3.842423 1.006877 0.869015

H 3.834472 0.992535 -0.908808

H 3.660540 -2.024398 -1.265978

H 2.647805 -0.889654 -2.175816

H 1.895063 -2.229096 -1.282214

H 3.671320 -2.003874 1.276733

H 1.905938 -2.207195 1.312449

H 2.667452 -0.853741 2.176968

H -4.740294 -0.231798 -0.014060

H -3.834432 0.992570 -0.908803

H -3.842470 1.006841 0.869020

H -3.671333 -2.003861 1.276737

H -2.667478 -0.853715 2.176970

H -1.905950 -2.207177 1.312475

H -3.660534 -2.024402 -1.265977

H -1.895058 -2.229115 -1.282182

H -2.647774 -0.889676 -2.175812

Zero-point correction= 0.346382 Hartree/Particle

Thermal correction to Energy= 0.364290

Thermal correction to Enthalpy= 0.365234

Thermal correction to Gibbs Free Energy= 0.301226

Sum of electronic and zero-point Energies= -660.314753

Sum of electronic and thermal Energies= -660.296845

Sum of electronic and thermal Enthalpies= -660.295900

Sum of electronic and thermal Free Energies= -660.359908

# 2. The information of transition state structures

The information for the calculated transition state structures are described. Coordinates, thermochemical values, and the wave number of imaginary frequency were calculated at UB3LYP/6-31G* and UM06-2X/6-31G* level. The total energy of nuclei and electrons was calculated at UB3LYP/6-311+G** level.

Entry **1** (TS1) (UB3LYP)

C 0.382330 0.000001 0.120381

C 0.616368 -1.282579 -0.657906

C 0.514479 0.000002 1.483435

C 0.616354 1.282586 -0.657905

H 0.520745 -0.925748 2.052790

H 0.520734 0.925751 2.052791

H 0.348443 -2.165388 -0.068803

H 0.046812 -1.307455 -1.592432

H 1.679703 -1.368894 -0.923884

H 1.679687 1.368914 -0.923881

H 0.046798 1.307454 -1.592432

H 0.348416 2.165390 -0.068802

C -1.924223 -0.000009 -0.067040

H -2.191239 -0.920322 0.443193

H -2.191237 0.920323 0.443161

H -2.040714 -0.000027 -1.147502

Zero-point correction= 0.141845 Hartree/Particle

Thermal correction to Energy= 0.149486

Thermal correction to Enthalpy= 0.150431

Thermal correction to Gibbs Free Energy= 0.110910

Sum of electronic and zero-point Energies= -196.908174

Sum of electronic and thermal Energies= -196.900532

Sum of electronic and thermal Enthalpies= -196.899588

Sum of electronic and thermal Free Energies= -196.939109

The wave number of the imaginary frequency= -483.9726 cm^-1^

Entry **1** (TS1) (UM06-2X)

C 0.375013 0.000000 0.114120

C 0.580111 -1.278457 -0.670534

C 0.549160 0.000000 1.465350

C 0.580110 1.278457 -0.670534

H 0.576910 -0.926899 2.030497

H 0.576909 0.926899 2.030497

H 0.314411 -2.156591 -0.075529

H -0.011941 -1.291245 -1.590063

H 1.635328 -1.370027 -0.957161

H 1.635327 1.370028 -0.957161

H -0.011942 1.291245 -1.590063

H 0.314409 2.156591 -0.075528

C -1.874408 -0.000001 -0.022907

H -2.129413 -0.918652 0.494631

H -2.129414 0.918651 0.494631

H -2.030493 0.000000 -1.097722

Zero-point correction= 0.143247 (Hartree/Particle)

Thermal correction to Energy= 0.150727

Thermal correction to Enthalpy= 0.151671

Thermal correction to Gibbs Free Energy= 0.112505

Sum of electronic and zero-point Energies= -196.781976

Sum of electronic and thermal Energies= -196.774496

Sum of electronic and thermal Enthalpies= -196.773551

Sum of electronic and thermal Free Energies= -196.812717

The wave number of the imaginary frequency= -683.0450 cm^-1^

Entry **1** (TS2) (UB3LYP)

C 0.050878 2.385886 0.562973

C 0.000823 0.295694 -0.460481

H -0.664563 2.937101 -0.037380

H -0.237607 2.212879 1.596749

H 1.086607 2.670937 0.409654

C -1.386617 -0.264574 0.059102

C -0.029543 0.775092 -1.754281

C 1.382012 -0.274462 0.048629

C -1.416694 -0.722761 1.532913

C -2.520293 0.783642 -0.077861

C -1.802191 -1.478996 -0.817847

H -1.906332 -1.190738 -1.868488

H -1.080934 -2.297492 -0.767537

H -2.769540 -1.869025 -0.476933

H -2.403784 1.603756 0.634446

H -2.587145 1.213759 -1.080937

H -3.482235 0.300419 0.130904

H -0.754534 -1.569417 1.732475

H -1.159949 0.088450 2.220655

H -2.434026 -1.049170 1.780719

H -0.964506 0.994834 -2.257710

H 0.861266 1.065560 -2.294647

C 2.565922 0.334010 -0.743988

C 1.707679 -0.034153 1.544266

C 1.444060 -1.804756 -0.206225

H 0.753438 -2.368303 0.427161

H 1.222031 -2.040248 -1.252230

H 2.454847 -2.169518 0.014310

H 2.578428 0.007268 -1.788565

H 2.558300 1.428507 -0.727880

H 3.507412 0.002761 -0.291736

H 1.852670 1.025888 1.764217

H 0.947689 -0.418242 2.222935

H 2.646252 -0.547697 1.786626

Zero-point correction= 0.314188 Hartree/Particle

Thermal correction to Energy= 0.328563

Thermal correction to Enthalpy= 0.329507

Thermal correction to Gibbs Free Energy= 0.275927

Sum of electronic and zero-point Energies= -432.585079

Sum of electronic and thermal Energies= -432.570704

Sum of electronic and thermal Enthalpies= -432.569760

Sum of electronic and thermal Free Energies= -432.623340

The wave number of the imaginary frequency= -445.7604 cm^-1^

Entry **1** (TS2) (UM06-2X)

C 0.047658 2.333575 0.552425

C 0.002271 0.288453 -0.471605

H -0.684606 2.881923 -0.028953

H -0.214849 2.148379 1.591171

H 1.078251 2.623587 0.376689

C -1.370517 -0.258219 0.057894

C -0.027365 0.757822 -1.758939

C 1.367376 -0.267336 0.050524

C -1.392119 -0.710969 1.525337

C -2.493663 0.791042 -0.066482

C -1.793285 -1.464408 -0.810878

H -1.912525 -1.168143 -1.857101

H -1.066619 -2.278561 -0.773373

H -2.753034 -1.856790 -0.455038

H -2.390647 1.580098 0.682093

H -2.533918 1.259336 -1.052978

H -3.458769 0.300900 0.102951

H -0.722062 -1.550194 1.727367

H -1.143849 0.107567 2.207250

H -2.407403 -1.042631 1.770812

H -0.964565 0.960358 -2.265591

H 0.862943 1.043395 -2.302294

C 2.545769 0.315401 -0.751378

C 1.696375 0.002751 1.532625

C 1.415214 -1.793256 -0.177270

H 0.723154 -2.339484 0.468988

H 1.182325 -2.035811 -1.219066

H 2.424658 -2.159898 0.041753

H 2.557074 -0.044589 -1.784201

H 2.530648 1.409805 -0.767220

H 3.484439 -0.000394 -0.284581

H 1.905604 1.059425 1.713385

H 0.914723 -0.311345 2.221723

H 2.602743 -0.556075 1.792703

Zero-point correction= 0.316960 (Hartree/Particle)

Thermal correction to Energy= 0.330964

Thermal correction to Enthalpy= 0.331908

Thermal correction to Gibbs Free Energy= 0.279198

Sum of electronic and zero-point Energies= -432.351824

Sum of electronic and thermal Energies= -432.337820

Sum of electronic and thermal Enthalpies= -432.336876

Sum of electronic and thermal Free Energies= -432.389585

The wave number of the imaginary frequency= -644.8025 cm^-1^

Entry **2** (TS1)

C 1.590594 -0.006447 1.150985

C 0.858354 0.000366 -0.012608

H 1.848152 -0.935161 1.653510

H 1.846253 0.916212 1.665486

C -1.254479 -0.007254 0.874533

C 0.749084 1.286785 -0.811284

C 0.751653 -1.275755 -0.827954

H 1.602341 -1.349558 -1.520566

H 0.766990 -2.164625 -0.188766

H -0.158235 -1.304408 -1.436082

H 0.761206 2.167199 -0.160427

H 1.600368 1.372059 -1.501845

H -0.160190 1.320916 -1.420032

H -1.145833 -0.920100 1.457012

H -1.146517 0.895839 1.472135

C -2.340942 0.001002 -0.160710

H -2.301111 0.894447 -0.795479

H -2.299138 -0.880536 -0.811819

H -3.339863 -0.004474 0.309102

Zero-point correction= 0.171070 Hartree/Particle

Thermal correction to Energy= 0.179933

Thermal correction to Enthalpy= 0.180877

Thermal correction to Gibbs Free Energy= 0.137330

Sum of electronic and zero-point Energies= -236.195850

Sum of electronic and thermal Energies= -236.186986

Sum of electronic and thermal Enthalpies= -236.186042

Sum of electronic and thermal Free Energies= -236.229589

The wave number of the imaginary frequency= -481.8314 cm^-1^

Entry **2** (TS2)

C -0.082313 -0.374246 1.954117

C -0.061010 -0.169706 0.576536

H 0.824233 -0.579638 2.512193

H -0.962576 -0.202119 2.558852

C 0.272107 2.064254 0.701926

C 1.255330 -0.714947 -0.120448

C -1.472281 -0.237505 -0.140380

C -1.770251 -1.685555 -0.612687

C -2.621586 0.121422 0.836448

C -1.652878 0.702382 -1.356172

C 2.530760 -0.105552 0.516376

C 1.369488 -2.251184 0.094668

C 1.359551 -0.453485 -1.638143

H -0.753258 2.294579 0.983323

H 0.920530 2.037272 1.574942

C 0.806685 2.892829 -0.442978

H 1.831377 2.622352 -0.718607

H 0.189166 2.825561 -1.343475

H 0.832052 3.957826 -0.153929

H 2.609027 0.968539 0.340502

H 2.588420 -0.274985 1.595182

H 3.414357 -0.578009 0.070967

H 0.583498 -0.967616 -2.211367

H 1.317703 0.611274 -1.879732

H 2.323633 -0.832783 -1.998601

H 1.369326 -2.503237 1.159752

H 0.558020 -2.806470 -0.379023

H 2.310780 -2.613730 -0.337564

H -2.764480 -0.640256 1.609308

H -2.466944 1.085261 1.331822

H -3.559227 0.189089 0.273091

H -1.137401 -1.999617 -1.446910

H -1.644901 -2.402201 0.205211

H -2.810053 -1.746988 -0.957139

H -1.712620 1.748894 -1.047820

H -0.862837 0.614471 -2.101164

H -2.599444 0.460282 -1.855387

Zero-point correction= 0.343670 Hartree/Particle

Thermal correction to Energy= 0.359180

Thermal correction to Enthalpy= 0.360124

Thermal correction to Gibbs Free Energy= 0.304097

Sum of electronic and zero-point Energies= -471.867267

Sum of electronic and thermal Energies= -471.851757

Sum of electronic and thermal Enthalpies= -471.850813

Sum of electronic and thermal Free Energies= -471.906840

The wave number of the imaginary frequency= -477.0941 cm^-1^

Entry **3** (TS1)

C -1.356597 1.393174 -0.387516

C -1.056466 0.109637 0.023089

H -1.622299 1.610738 -1.419016

H -1.241182 2.244714 0.277490

C -1.536482 -1.049687 -0.834804

C 1.148799 -0.032790 -0.491908

C -0.988975 -0.178738 1.513304

H -2.572246 -1.306473 -0.570807

H -1.521440 -0.794139 -1.899686

H -0.937925 -1.954908 -0.691315

H -0.550887 0.658776 2.066617

H -2.001345 -0.341890 1.909454

H -0.409228 -1.079508 1.737456

H 0.953291 -0.074951 -1.564149

C 1.778928 1.252794 -0.031117

C 1.730752 -1.298952 0.076001

H 1.799406 -1.260646 1.170381

H 1.157895 -2.191265 -0.199872

H 2.757940 -1.456606 -0.296828

H 1.259311 2.125776 -0.437720

H 1.775619 1.333463 1.064209

H 2.833333 1.314294 -0.348507

Zero-point correction= 0.199653 Hartree/Particle

Thermal correction to Energy= 0.209793

Thermal correction to Enthalpy= 0.210738

Thermal correction to Gibbs Free Energy= 0.164465

Sum of electronic and zero-point Energies= -275.484783

Sum of electronic and thermal Energies= -275.474643

Sum of electronic and thermal Enthalpies= -275.473699

Sum of electronic and thermal Free Energies= -275.519971

The wave number of the imaginary frequency= -487.9975 cm^-1^

Entry **3** (TS2)

C 0.050253 0.166858 -1.917060

C 0.153663 0.138860 -0.516225

H -0.683712 0.765882 -2.438639

H 0.600929 -0.530558 -2.539557

C -0.392446 1.441986 0.212973

C -1.361155 -1.460894 -0.205619

C 1.512565 -0.545186 -0.023895

C -1.464067 -2.101845 1.165797

H -0.851619 -2.112669 -0.911088

C -2.701162 -1.032371 -0.763472

H -2.607901 -0.562989 -1.747051

H -3.346358 -1.918231 -0.886835

H -3.239440 -0.347044 -0.098583

H -0.507216 -2.469114 1.545082

H -1.890167 -1.430821 1.917183

H -2.136024 -2.975673 1.106046

C 1.644309 -2.016327 -0.500184

C 2.729347 0.192815 -0.656368

C 1.738923 -0.567038 1.504722

H 1.841227 0.431290 1.936162

H 0.941941 -1.086596 2.040009

H 2.673981 -1.100561 1.715687

H 0.987645 -2.698441 0.043171

H 1.448069 -2.136248 -1.569806

H 2.672371 -2.353918 -0.323556

H 2.700646 0.140891 -1.749068

H 2.785278 1.244509 -0.375285

H 3.659108 -0.285369 -0.322850

C 0.724241 2.511250 0.368945

C -1.490665 2.156868 -0.617941

C -1.007579 1.195932 1.609792

H -1.959884 0.665442 1.538701

H -0.356398 0.633549 2.279000

H -1.212659 2.161831 2.087925

H 1.508668 2.223499 1.072145

H 1.188960 2.741293 -0.595047

H 0.277057 3.437628 0.750424

H -1.087622 2.606487 -1.530940

H -2.314590 1.497832 -0.894552

H -1.909644 2.972655 -0.017470

Zero-point correction= 0.372786 Hartree/Particle

Thermal correction to Energy= 0.389419

Thermal correction to Enthalpy= 0.390363

Thermal correction to Gibbs Free Energy= 0.332202

Sum of electronic and zero-point Energies= -511.147606

Sum of electronic and thermal Energies= -511.130974

Sum of electronic and thermal Enthalpies= -511.130029

Sum of electronic and thermal Free Energies= -511.188191

The wave number of the imaginary frequency= -480.7800 cm^-1^

Entry **4** (TS1)

C 1.419648 -0.000358 -1.473973

C 1.193417 -0.000073 -0.104325

H 1.464703 0.924520 -2.042511

H 1.464446 -0.925467 -2.042152

C 1.507586 1.276502 0.661428

C -1.055705 0.000013 0.013346

C 1.507330 -1.276405 0.661955

C -1.442388 1.266190 -0.716064

C -1.442670 -1.264154 -0.719391

C -1.440212 -0.001799 1.475297

H -1.081635 -0.891970 2.003819

H -1.079280 0.885751 2.006659

H -2.540029 -0.000454 1.573467

H -1.095066 2.166082 -0.196099

H -1.044586 1.281482 -1.736290

H -2.540239 1.343900 -0.791315

H -1.045448 -1.276474 -1.739876

H -1.094849 -2.165471 -0.202229

H -2.540552 -1.341840 -0.794194

H 1.246976 -2.170716 0.087245

H 2.584964 -1.324332 0.872688

H 0.992817 -1.323818 1.626561

H 2.585025 1.323617 0.873355

H 1.248673 2.170626 0.085792

H 0.992043 1.325071 1.625444

Zero-point correction= 0.228466 Hartree/Particle

Thermal correction to Energy= 0.239633

Thermal correction to Enthalpy= 0.240578

Thermal correction to Gibbs Free Energy= 0.192618

Sum of electronic and zero-point Energies= -314.772102

Sum of electronic and thermal Energies= -314.760934

Sum of electronic and thermal Enthalpies= -314.759990

Sum of electronic and thermal Free Energies= -314.807950

The wave number of the imaginary frequency= -493.0287 cm^-1^

Entry **4** (TS2)

C 0.078837 -0.024257 -1.874069

C 0.243462 0.004908 -0.460616

H -0.172293 0.858757 -2.447474

H 0.014336 -0.961397 -2.415774

C 0.844699 1.401231 0.062467

C -1.861502 -0.049930 0.090830

C 0.941199 -1.350479 0.065170

C 0.088195 -2.617336 -0.195897

C 2.261091 -1.627644 -0.718802

C 1.292912 -1.382165 1.571147

C -2.477586 1.324710 -0.159578

C -2.561646 -1.019174 -0.863632

C -2.062404 -0.465238 1.544280

C 2.397913 1.429955 -0.026660

C 0.430112 2.602351 -0.834001

C 0.492152 1.775224 1.523523

H -0.779265 -2.686439 0.460347

H -0.256455 -2.691497 -1.230742

H 0.705828 -3.501488 0.000111

H 2.065661 -0.661007 1.846224

H 0.424686 -1.210620 2.210601

H 1.687108 -2.375374 1.819731

H 2.074134 -1.720715 -1.792753

H 3.025103 -0.866126 -0.579136

H 2.686284 -2.578064 -0.373055

H 2.891977 0.772299 0.691044

H 2.746336 1.179007 -1.032693

H 2.737400 2.449587 0.193019

H -0.561002 2.024482 1.654498

H 0.739600 0.988976 2.236547

H 1.066512 2.664747 1.810691

H 0.932467 2.567755 -1.805975

H -0.639751 2.680616 -1.012278

H 0.748400 3.529953 -0.344053

H -1.713456 -1.478859 1.753521

H -1.584671 0.213888 2.253979

H -3.142542 -0.453733 1.770594

H -2.083038 2.120608 0.473080

H -2.386641 1.629350 -1.206797

H -3.556601 1.259773 0.055293

H -2.330003 -0.795509 -1.909908

H -2.334523 -2.068333 -0.680451

H -3.650186 -0.903572 -0.743157

Zero-point correction= 0.402315 Hartree/Particle

Thermal correction to Energy= 0.419818

Thermal correction to Enthalpy= 0.420762

Thermal correction to Gibbs Free Energy= 0.361294

Sum of electronic and zero-point Energies= -550.419799

Sum of electronic and thermal Energies= -550.402295

Sum of electronic and thermal Enthalpies= -550.401351

Sum of electronic and thermal Free Energies= -550.460819

The wave number of the imaginary frequency= -442.5454 cm^-1^

Entry **5** (TS1)

C 3.627171 0.652185 0.611061

C 3.044822 -0.567293 0.181806

C 3.669284 -1.219255 -1.037782

C 2.616716 -1.519948 1.276614

H 3.431120 1.049166 1.602032

H 4.177154 1.282343 -0.081660

H 3.022053 -2.008417 -1.431249

H 4.638102 -1.662493 -0.777162

H 3.832089 -0.481936 -1.830020

H 2.078818 -2.380630 0.868036

H 1.970877 -1.010481 1.997625

H 3.496101 -1.896435 1.813724

O 1.526853 -0.105619 -0.652917

N 0.436567 0.228497 0.089703

C -0.726776 -0.738304 -0.017676

C -1.910518 0.212129 -0.420579

C -0.493494 -1.816381 -1.079696

C -0.979607 -1.388697 1.355043

C 0.003761 1.655166 -0.094290

C -1.509989 1.568003 0.162498

C 0.299243 2.167096 -1.517252

C 0.697579 2.556013 0.934812

C -3.272518 -0.253562 0.043400

O -3.782257 -1.216887 -0.770697

O -3.871797 0.144564 1.018577

H 1.775166 2.603475 0.751430

H 1.367948 2.105038 -1.731170

H 0.536689 2.177056 1.949575

H 0.299475 3.576518 0.878949

H -0.099056 -1.950830 1.672807

H -0.226975 1.583944 -2.280780

H 0.322213 -2.486195 -0.794637

H -0.017925 3.212338 -1.608485

H -0.240542 -1.373444 -2.046919

H -1.200528 -0.639743 2.121550

H -1.733514 1.587906 1.233735

H -1.938579 0.258724 -1.514539

H -2.051335 2.399939 -0.298774

H -1.823551 -2.087257 1.307422

H -1.405444 -2.411101 -1.193962

H -4.642767 -1.466336 -0.382897

Zero-point correction= 0.356830 Hartree/Particle

Thermal correction to Energy= 0.376571

Thermal correction to Enthalpy= 0.377515

Thermal correction to Gibbs Free Energy= 0.309386

Sum of electronic and zero-point Energies= -789.792446

Sum of electronic and thermal Energies= -789.772705

Sum of electronic and thermal Enthalpies= -789.771761

Sum of electronic and thermal Free Energies= -789.839890

The wave number of the imaginary frequency= -578.4628 cm^-1^

Entry **5** (TS2)

C 2.296917 1.041922 1.378893

C 2.155471 0.098463 0.308347

C 2.982469 0.558617 -0.974249

C 2.194068 -1.399101 0.822304

H 2.034945 0.802202 2.398865

H 2.574327 2.071145 1.188693

C 2.912969 -0.407233 -2.176072

C 4.477882 0.750500 -0.606667

C 2.491177 1.933151 -1.504270

C 1.732963 -2.453865 -0.208692

C 1.299412 -1.546267 2.085944

C 3.626611 -1.796684 1.262831

O 0.551681 0.426089 -0.383834

N -0.642409 0.292251 0.319381

C -1.672425 -0.538414 -0.445003

C -2.972305 0.343570 -0.352897

C -1.314526 -0.761126 -1.921471

C -1.875237 -1.883967 0.274525

C -1.238542 1.609018 0.741891

C -2.727281 1.247136 0.849363

C -1.011824 2.735214 -0.285269

C -0.711236 2.054319 2.111558

C -4.259445 -0.445316 -0.252211

O -4.667459 -0.903077 -1.466789

O -4.884492 -0.671159 0.760842

H 0.330639 2.378560 2.061347

H 0.052028 2.949151 -0.394992

H -0.788664 1.241742 2.841051

H -1.305580 2.901224 2.476247

H -0.939821 -2.441284 0.329471

H -1.403824 2.481375 -1.275722

H -0.463557 -1.433057 -2.046324

H -1.513888 3.649618 0.052309

H -1.072035 0.182950 -2.417398

H -2.242307 -1.737496 1.294833

H -2.930204 0.696902 1.773721

H -3.036996 0.939625 -1.268977

H -3.365560 2.136409 0.848848

H -2.602019 -2.503401 -0.264711

H -2.176462 -1.207140 -2.429180

H -5.484133 -1.411590 -1.300752

H 1.764682 -1.102227 2.971876

H 0.317719 -1.091562 1.943938

H 1.163453 -2.610484 2.308387

H 0.826087 -2.148541 -0.724379

H 2.493098 -2.664250 -0.961830

H 1.530563 -3.399672 0.306835

H 4.302797 -1.935358 0.414674

H 4.066319 -1.057835 1.941051

H 3.579562 -2.754433 1.796460

H 3.489746 -1.321117 -2.014426

H 1.883421 -0.676346 -2.421860

H 3.346920 0.088156 -3.053012

H 1.498206 1.862559 -1.946493

H 2.465764 2.707486 -0.732053

H 3.189623 2.276004 -2.277183

H 4.611223 1.487696 0.191468

H 4.964362 -0.173937 -0.291708

H 5.015876 1.116713 -1.489614

Zero-point correction= 0.528768 Hartree/Particle

Thermal correction to Energy= 0.555595

Thermal correction to Enthalpy= 0.556539

Thermal correction to Gibbs Free Energy= 0.475232

Sum of electronic and zero-point Energies= -1025.456679

Sum of electronic and thermal Energies= -1025.429853

Sum of electronic and thermal Enthalpies= -1025.428909

Sum of electronic and thermal Free Energies= -1025.510216

The wave number of the imaginary frequency= -500.2352 cm^-1^

Entry **6** (TS1)

C -3.728552 0.114870 -0.706762

C -3.045220 -1.046067 -0.251106

C -3.658179 -1.748009 0.948332

C -2.534395 -1.972841 -1.335123

H -3.548167 0.519079 -1.697633

H -4.353093 0.695468 -0.034201

H -2.958835 -2.475013 1.371828

H -4.571454 -2.277128 0.651181

H -3.918285 -1.026624 1.729047

H -1.918480 -2.774920 -0.918471

H -1.945009 -1.422604 -2.073610

H -3.380207 -2.436649 -1.857918

O -1.611595 -0.485534 0.618939

N -0.556212 0.038531 -0.063558

C 0.710464 -0.799257 -0.073245

C 1.781290 0.238109 0.435991

C 0.585726 -1.982925 0.920197

C 0.985353 -1.367341 -1.495385

C -0.321471 1.498054 0.218265

C 1.209241 1.624050 0.089444

C -0.858824 1.938096 1.605209

C -1.035167 2.331218 -0.879100

C 3.197307 0.096030 -0.087563

O 4.046376 -0.384864 0.859834

O 3.593601 0.397129 -1.194022

H -2.113714 2.154698 -0.797519

H -1.943610 1.792735 1.597149

H -0.728445 1.928167 -1.850272

C -0.769230 3.843573 -0.867649

H 0.295351 -2.207530 -1.638279

C -0.286996 1.268637 2.858351

H -0.298479 -2.565423 0.646976

H -0.688896 3.018600 1.683984

H 0.378268 -1.579713 1.916884

C 0.858006 -0.425226 -2.696079

H 1.493961 1.888802 -0.932570

H 1.834370 0.129216 1.520858

H 1.611388 2.398599 0.750895

H 1.990238 -1.804415 -1.503081

C 1.793417 -2.927450 1.006156

H 4.924126 -0.427342 0.433905

H -0.475804 0.191881 2.855754

H 0.790560 1.436303 2.970582

H -0.768340 1.685182 3.751212

H 1.983204 -3.444874 0.059860

H 2.710856 -2.407304 1.297105

H 1.598788 -3.697804 1.761498

H -0.139737 0.019657 -2.748593

H 1.602118 0.373660 -2.664670

H 1.022107 -0.989278 -3.622109

H -1.164074 4.332633 0.029103

H 0.301367 4.072740 -0.929968

H -1.252268 4.314513 -1.731623

Zero-point correction= 0.472603 Hartree/Particle

Thermal correction to Energy= 0.497466

Thermal correction to Enthalpy= 0.498410

Thermal correction to Gibbs Free Energy= 0.419752

Sum of electronic and zero-point Energies= -946.914290

Sum of electronic and thermal Energies= -946.889428

Sum of electronic and thermal Enthalpies= -946.888484

Sum of electronic and thermal Free Energies= -946.967142

The wave number of the imaginary frequency= -579.3832 cm^-1^

The total energy of nuclei and electrons= -947.660208263 Hartree/Particle

Entry **6** (TS2)

C 2.585420 1.528416 0.625428

C 2.436104 0.171944 0.162427

C 3.199907 -0.031551 -1.226472

C 2.607103 -0.870276 1.346812

H 2.450809 1.804694 1.660456

H 2.762084 2.341978 -0.066757

C 3.146460 -1.465160 -1.795937

C 4.694621 0.362019 -1.084260

C 2.626782 0.890504 -2.336340

C 2.142448 -2.311039 1.048156

C 1.803031 -0.394939 2.587228

C 4.088080 -0.963035 1.797299

O 0.813664 0.092855 -0.500469

N -0.361086 0.254453 0.241062

C -1.389672 -0.857612 -0.013660

C -2.643769 -0.049331 -0.511587

C -0.917322 -1.814149 -1.140558

C -1.635683 -1.673941 1.287079

C -0.983232 1.634996 0.103700

C -2.494970 1.337581 0.125259

C -0.545964 2.405516 -1.168353

C -0.593416 2.476097 1.349852

C -4.025463 -0.619312 -0.252727

O -4.647853 -0.972112 -1.410964

O -4.588387 -0.723719 0.816810

H 0.478314 2.693416 1.305304

H 0.543990 2.465610 -1.155772

H -0.745413 1.845823 2.232215

C -1.356359 3.794054 1.554844

H -0.733722 -2.269506 1.464702

C -0.999161 1.905285 -2.544070

H 0.042818 -2.246864 -0.858236

H -0.911743 3.433029 -1.053869

H -0.718951 -1.217328 -2.036611

C -1.987776 -0.935479 2.584292

H -2.876190 1.314205 1.149333

H -2.550340 0.043235 -1.594111

H -3.065706 2.098157 -0.417916

H -2.433849 -2.397517 1.086498

C -1.874592 -2.957704 -1.510263

H -5.529944 -1.299051 -1.148840

H 2.282604 0.443438 3.102729

H 0.789848 -0.104447 2.310623

H 1.744668 -1.213650 3.313103

H 1.100491 -2.344571 0.740671

H 2.739253 -2.809060 0.285490

H 2.236209 -2.905281 1.964673

H 4.707091 -1.525917 1.093006

H 4.537053 0.024857 1.942884

H 4.129824 -1.493654 2.756674

H 3.782598 -2.160241 -1.241252

H 2.127210 -1.857234 -1.818410

H 3.520755 -1.451193 -2.826607

H 1.622194 0.593471 -2.632048

H 2.599036 1.945074 -2.047967

H 3.279343 0.818435 -3.214706

H 4.815705 1.399382 -0.755999

H 5.243377 -0.275254 -0.390208

H 5.178714 0.267223 -2.064071

H -1.173286 4.517799 0.753258

H -2.439083 3.637706 1.622335

H -1.037831 4.264610 2.492188

H -0.662474 0.884388 -2.744239

H -2.088513 1.937286 -2.661908

H -0.575930 2.547693 -3.325323

H -2.032873 -3.652179 -0.678614

H -2.852347 -2.597255 -1.842601

H -1.442726 -3.536378 -2.335118

H -1.243314 -0.177170 2.837666

H -2.971411 -0.466617 2.527094

H -2.015594 -1.655801 3.410942

Zero-point correction= 0.644187 Hartree/Particle

Thermal correction to Energy= 0.676366

Thermal correction to Enthalpy= 0.677310

Thermal correction to Gibbs Free Energy= 0.584332

Sum of electronic and zero-point Energies= -1182.573690

Sum of electronic and thermal Energies= -1182.541511

Sum of electronic and thermal Enthalpies= -1182.540567

Sum of electronic and thermal Free Energies= -1182.633545

The wave number of the imaginary frequency= -488.2521 cm^-1^

The total energy of nuclei and electrons= -1183.55237316 Hartree/Particle

Entry **7** (TS1)

C 2.858424 2.495686 -0.883418

C 1.638643 2.869469 -0.259328

C 1.769851 3.655972 1.033078

C 0.549776 3.354689 -1.192043

H 2.885741 2.185925 -1.923355

H 3.773107 2.396611 -0.306382

H 0.810897 3.708550 1.556853

H 2.105088 4.678278 0.820274

H 2.499984 3.187376 1.699950

H -0.389323 3.520422 -0.656484

H 0.378365 2.633816 -1.996596

H 0.848005 4.306406 -1.649616

O 0.997700 1.408914 0.536245

N 0.439650 0.388985 -0.180622

C -1.054624 0.170596 0.075690

C -1.128405 -1.397966 0.217123

C -1.507702 0.882753 1.368677

C -1.851582 0.716026 -1.136556

C 1.224211 -0.884886 -0.080378

C 0.153009 -1.911129 -0.457385

C 1.789993 -1.117512 1.345254

C 2.385664 -0.861319 -1.091407

C -2.331599 -2.102870 -0.375414

O -3.235651 -2.466706 0.573256

O -2.495800 -2.371112 -1.546812

H 2.999148 0.023738 -0.885488

H 2.350627 -0.219830 1.625417

H 1.976525 -0.736496 -2.101604

C 3.278410 -2.110357 -1.011186

H -1.467541 1.717668 -1.341528

H 0.971224 -1.205725 2.070357

H -1.155736 1.918125 1.321390

C 2.706358 -2.349198 1.440719

H -1.003369 0.431308 2.231727

H -1.634524 0.109614 -2.022410

H 0.003882 -1.935225 -1.541748

H -1.111295 -1.638537 1.283598

H 0.399547 -2.926652 -0.135110

C -3.375583 0.823796 -0.928109

C -3.030862 0.889753 1.569265

H -3.955753 -2.921401 0.095487

C -3.734006 1.548595 0.375633

H -3.407301 -0.133179 1.699496

H -3.265752 1.428415 2.496477

H -3.838898 -0.168981 -0.923336

H -3.808539 1.348116 -1.789866

H -3.422868 2.602103 0.310099

H -4.821844 1.551848 0.521585

H 2.122346 -3.268872 1.291529

C 3.838662 -2.300519 0.405255

H 3.120413 -2.415134 2.455280

H 2.706069 -3.003138 -1.301751

H 4.097415 -2.021867 -1.736463

H 4.509087 -1.461237 0.642415

H 4.445899 -3.213184 0.459832

Zero-point correction= 0.490577 Hartree/Particle

Thermal correction to Energy= 0.513543

Thermal correction to Enthalpy= 0.514487

Thermal correction to Gibbs Free Energy= 0.438948

Sum of electronic and zero-point Energies= -1023.123522

Sum of electronic and thermal Energies= -1023.100556

Sum of electronic and thermal Enthalpies= -1023.099612

Sum of electronic and thermal Free Energies= -1023.175152

The wave number of the imaginary frequency= -588.6083 cm^-1^

Entry **7** (TS2)

C 2.782319 1.111060 1.008202

C 2.515938 -0.109987 0.296498

C 3.321931 -0.142235 -1.081357

C 2.500233 -1.360293 1.268248

H 2.567876 1.224862 2.060965

H 3.138561 1.995482 0.495001

C 3.162970 -1.434052 -1.910652

C 4.839075 0.057142 -0.820597

C 2.889090 1.018944 -2.016767

C 1.991093 -2.675073 0.629546

C 1.623973 -1.036881 2.512572

C 3.919669 -1.659534 1.817937

O 0.920450 0.079938 -0.425935

N -0.272800 0.277414 0.278777

C -1.375775 -0.711105 -0.148350

C -2.648478 0.218125 -0.232011

C -1.085044 -1.348555 -1.527002

C -1.503942 -1.806598 0.939763

C -0.775576 1.695444 0.204962

C -2.254292 1.482122 0.538873

C -0.576737 2.352508 -1.185074

C -0.098354 2.568741 1.277495

C -3.956290 -0.319817 0.313236

O -4.808820 -0.702062 -0.675498

O -4.272097 -0.379655 1.482691

H 0.982288 2.559329 1.104266

H 0.486671 2.295148 -1.430076

H -0.267022 2.120328 2.264172

C -0.578895 4.029868 1.251263

H -0.498888 -2.164937 1.161396

H -1.102083 1.781456 -1.960241

H -0.065040 -1.739398 -1.518628

C -1.024229 3.824923 -1.231708

H -1.103457 -0.572295 -2.301270

H -1.882322 -1.356566 1.863514

H -2.382887 1.305442 1.611588

H -2.821052 0.460563 -1.283656

H -2.882913 2.334134 0.266800

C -2.373273 -3.020859 0.554041

H -5.618263 -1.010846 -0.225013

H 2.145045 -0.377814 3.214848

H 0.677929 -0.568752 2.237565

H 1.412445 -1.963845 3.056818

H 1.132804 -2.519963 -0.020291

H 2.765529 -3.173963 0.044610

H 1.695780 -3.374987 1.419482

H 4.577817 -2.103505 1.065945

H 4.403089 -0.761913 2.217342

H 3.833170 -2.385294 2.636495

H 3.690552 -2.284134 -1.471443

H 2.114506 -1.703873 -2.052293

H 3.599575 -1.269861 -2.903219

H 1.889009 0.858442 -2.417865

H 2.905355 1.996330 -1.525895

H 3.592243 1.070370 -2.857013

H 5.049440 1.003692 -0.313103

H 5.280179 -0.745460 -0.227803

H 5.362781 0.074684 -1.784124

H -2.116169 3.894766 -1.125345

H -0.790051 4.242064 -2.219731

C -0.355923 4.659745 -0.130611

H -1.645280 4.083990 1.512487

H -0.045994 4.602781 2.021005

H 0.724830 4.718227 -0.328132

H -0.733906 5.690014 -0.149727

H -3.435500 -2.752433 0.553175

C -2.006282 -3.593975 -0.821266

H -2.262752 -3.787963 1.331395

C -2.044161 -2.493529 -1.889285

H -0.996185 -4.027943 -0.784687

H -2.689092 -4.411781 -1.085334

H -3.070858 -2.119349 -1.994896

H -1.758688 -2.899660 -2.868337

Zero-point correction= 0.662200 Hartree/Particle

Thermal correction to Energy= 0.692426

Thermal correction to Enthalpy= 0.693370

Thermal correction to Gibbs Free Energy= 0.604346

Sum of electronic and zero-point Energies= -1258.786510

Sum of electronic and thermal Energies= -1258.756284

Sum of electronic and thermal Enthalpies= -1258.755340

Sum of electronic and thermal Free Energies= -1258.844364

The wave number of the imaginary frequency= -484.4497 cm^-1^

Entry **8** (TS1)

C -3.131059 -0.011161 -0.390343

C -2.294444 -1.246869 -0.710659

C -0.946875 -1.300244 0.044800

N -0.229372 0.005264 -0.148895

C -0.962035 1.300741 0.051121

C -2.307264 1.234032 -0.707410

H -4.053062 -0.015202 -0.985587

H -3.444716 -0.013957 0.661145

H -2.847151 -2.167668 -0.484707

H -2.087937 -1.258432 -1.789843

O 0.951379 0.009242 0.571303

H -2.098607 1.250601 -1.786148

H -2.870227 2.148235 -0.480032

C -0.104505 -2.408454 -0.610636

H 0.811031 -2.600684 -0.048900

H -0.680983 -3.340505 -0.640406

H 0.160853 -2.133816 -1.636208

C -1.157315 -1.671324 1.531124

H -0.222888 -1.537345 2.082333

H -1.930366 -1.069402 2.015372

H -1.458644 -2.722309 1.615961

C -1.181288 1.660333 1.539271

H -1.494668 2.707390 1.629443

H -1.948602 1.046924 2.018155

H -0.246716 1.533834 2.092174

C -0.131738 2.424919 -0.592645

H -0.734777 3.338443 -0.654896

H 0.757217 2.653418 -0.003319

H 0.175951 2.145526 -1.605601

C 2.544249 -0.004737 -0.193803

C 2.413360 -0.152885 -1.603792

H 2.255940 0.708625 -2.245101

H 2.316593 -1.133565 -2.057876

C 3.102857 1.323667 0.292529

C 3.146753 -1.180842 0.558971

H 2.750057 2.162739 -0.311133

H 2.818367 1.496430 1.335446

H 4.198758 1.309639 0.237040

H 2.878550 -1.127238 1.619010

H 2.805452 -2.140069 0.162539

H 4.240978 -1.155459 0.481820

Zero-point correction= 0.371973 Hartree/Particle

Thermal correction to Energy= 0.389468

Thermal correction to Enthalpy= 0.390412

Thermal correction to Gibbs Free Energy= 0.328901

Sum of electronic and zero-point Energies= -640.518123

Sum of electronic and thermal Energies= -640.500628

Sum of electronic and thermal Enthalpies= -640.499684

Sum of electronic and thermal Free Energies= -640.561195

The wave number of the imaginary frequency= -630.3954 cm^-1^

Entry **8** (TS2)

C 1.842315 0.085693 1.315173

C 2.049039 1.591282 1.621213

C 3.190625 -0.667723 1.393346

N 1.203927 -0.200738 -0.028934

C 4.131586 -0.363969 0.233298

C 2.058101 0.060686 -1.251857

C 3.407974 -0.678042 -1.070365

C 0.956658 -0.491369 2.428806

O -0.006306 0.545977 -0.090163

H 3.657869 -0.420364 2.355165

C 2.281328 1.555685 -1.577526

H 4.457628 0.683368 0.257263

H 2.984584 -1.746859 1.408659

C 1.401521 -0.581253 -2.486051

H 5.042914 -0.970019 0.314654

H 3.213116 -1.758594 -1.110769

H 4.037763 -0.438530 -1.936340

H 2.213530 1.727089 2.696660

H 2.911264 2.020788 1.107778

H 1.164496 2.165345 1.338035

H 1.495507 -0.435554 3.381841

H 0.031576 0.073350 2.537037

H 0.717147 -1.540984 2.239245

H 1.333192 2.096036 -1.523500

H 2.994244 2.045047 -0.911425

H 2.669680 1.653879 -2.598261

H 2.131298 -0.611037 -3.303953

H 1.083172 -1.606042 -2.276514

H 0.543042 -0.007590 -2.834849

C -1.616849 -0.029708 -0.471474

C -2.000424 -1.469448 0.101244

C -2.355695 1.275015 0.072940

C -1.600814 -0.072100 -1.915433

H -1.570525 0.830201 -2.509770

H -1.472967 -1.002295 -2.451605

C -0.885792 -2.498246 -0.210870

C -2.309211 -1.552256 1.611973

C -3.279933 -2.008408 -0.608675

H -4.166816 -1.405240 -0.409336

H -3.161350 -2.084118 -1.692640

H -3.480352 -3.018979 -0.231790

H -0.747778 -2.635934 -1.288200

H 0.076267 -2.203469 0.204660

H -1.176576 -3.472246 0.200860

H -1.491541 -1.201860 2.236963

H -3.214478 -1.001554 1.881825

H -2.490894 -2.602382 1.870541

C -2.049896 1.634654 1.545426

C -1.955529 2.526225 -0.756936

C -3.890763 1.136886 -0.073758

H -4.177294 0.835486 -1.086722

H -4.323920 0.425731 0.635005

H -4.353044 2.111254 0.126342

H -2.295743 0.839790 2.248135

H -1.001279 1.899644 1.678106

H -2.654232 2.507205 1.823030

H -0.871989 2.620924 -0.842382

H -2.391796 2.523818 -1.760947

H -2.338206 3.420410 -0.251807

Zero-point correction= 0.544005 Hartree/Particle

Thermal correction to Energy= 0.568626

Thermal correction to Enthalpy= 0.569570

Thermal correction to Gibbs Free Energy= 0.494978

Sum of electronic and zero-point Energies= -876.177242

Sum of electronic and thermal Energies= -876.152620

Sum of electronic and thermal Enthalpies= -876.151676

Sum of electronic and thermal Free Energies= -876.226269

The wave number of the imaginary frequency= -414.6194 cm^-1^

Entry **9** (TS1)

C 2.866498 0.010376 -0.112207

C 2.056357 1.242613 -0.477636

C 0.665085 1.299648 0.188968

N -0.043517 0.000244 -0.061415

C 0.666041 -1.297768 0.186393

C 2.058121 -1.229666 -0.479766

O 4.089650 0.082535 -0.849683

H 3.091317 0.012497 0.965567

H 2.614362 2.148507 -0.213514

H 1.936608 1.244758 -1.568651

O -1.278283 -0.002886 0.559672

H 1.939244 -1.230504 -1.570857

H 2.610151 -2.142785 -0.214168

C -0.124699 2.418106 -0.513571

H -1.059478 2.631423 0.006715

H 0.470360 3.338483 -0.524163

H -0.348172 2.140047 -1.548192

C 0.774176 1.665020 1.688436

H -0.200329 1.545670 2.169704

H 1.500673 1.052463 2.228101

H 1.084430 2.711159 1.796261

C 0.777258 -1.666417 1.685134

H 1.090748 -2.711970 1.791661

H 1.500176 -1.050992 2.226257

H -0.197917 -1.551599 2.165985

C -0.120407 -2.416803 -0.518856

H 0.473614 -3.338133 -0.526133

H -1.057576 -2.629034 -0.002277

H -0.339798 -2.139121 -1.554354

C -2.800887 -0.003728 -0.343464

C -2.537046 -0.016301 -1.742392

H -2.360400 -0.948087 -2.269907

H -2.350136 0.905029 -2.284567

C -3.438021 -1.256840 0.236826

C -3.433159 1.261940 0.214388

H -3.053849 -2.166975 -0.229555

H -3.253475 -1.310764 1.314566

H -4.523511 -1.232221 0.078308

H -3.247301 1.335557 1.290733

H -3.047028 2.161809 -0.269621

H -4.518916 1.237407 0.057509

H 4.603999 -0.712602 -0.638931

Zero-point correction= 0.376135 Hartree/Particle

Thermal correction to Energy= 0.394874

Thermal correction to Enthalpy= 0.395819

Thermal correction to Gibbs Free Energy= 0.331269

Sum of electronic and zero-point Energies= -715.722655

Sum of electronic and thermal Energies= -715.703916

Sum of electronic and thermal Enthalpies= -715.702972

Sum of electronic and thermal Free Energies= -715.767521

The wave number of the imaginary frequency= -625.9592 cm^-1^

Entry **9** (TS2)

C -1.610039 -0.007969 1.295151

C -1.729463 -1.448522 1.858284

C -2.997935 0.665066 1.229960

N -0.963219 0.080923 -0.072915

C -3.897994 0.108228 0.143459

C -1.777836 -0.434173 -1.239322

C -3.172806 0.236994 -1.188481

C -0.780744 0.806852 2.297943

O 0.287560 -0.591412 0.003566

H -3.483673 0.562980 2.207641

C -1.900530 -1.974551 -1.315077

H -4.131023 -0.947434 0.349598

H -2.874371 1.739176 1.041354

C -1.144844 0.038740 -2.558709

O -5.107334 0.871257 0.165881

H -3.064562 1.309058 -1.397445

H -3.779965 -0.188938 -1.999980

H -1.906039 -1.404725 2.939240

H -2.553147 -2.018298 1.423496

H -0.804440 -2.003160 1.688059

H -1.332532 0.884814 3.241659

H 0.173100 0.324862 2.508879

H -0.600292 1.819976 1.929164

H -0.916717 -2.433786 -1.192859

H -2.571409 -2.397399 -0.564964

H -2.289062 -2.262276 -2.299321

H -1.856653 -0.123024 -3.376874

H -0.904597 1.104586 -2.520930

H -0.239689 -0.520004 -2.796424

C 1.870025 0.002458 -0.463599

C 2.161770 1.539236 -0.146881

C 2.671562 -1.145070 0.302251

C 1.875039 -0.208142 -1.892872

H 1.906063 -1.200214 -2.320741

H 1.717817 0.606252 -2.586276

C 1.000388 2.431894 -0.651962

C 2.428687 1.898661 1.330394

C 3.427128 2.019012 -0.920793

H 4.342162 1.523829 -0.592941

H 3.338751 1.882020 -2.001585

H 3.553707 3.093321 -0.737817

H 0.901897 2.394717 -1.742054

H 0.040548 2.142165 -0.227172

H 1.213910 3.474695 -0.388456

H 1.614578 1.617655 1.993682

H 3.353420 1.451835 1.705152

H 2.550805 2.985764 1.405577

C 2.364417 -1.265449 1.813395

C 2.354872 -2.540878 -0.302240

C 4.198707 -0.945703 0.142582

H 4.484120 -0.801037 -0.904691

H 4.581331 -0.103374 0.725455

H 4.710676 -1.845513 0.504757

H 2.544213 -0.347142 2.370259

H 1.333757 -1.575476 1.984563

H 3.020203 -2.034069 2.240958

H 1.279764 -2.710161 -0.379831

H 2.809181 -2.687214 -1.287598

H 2.775354 -3.311911 0.353290

H -5.678876 0.532122 -0.540875

Zero-point correction= 0.547888 Hartree/Particle

Thermal correction to Energy= 0.573846

Thermal correction to Enthalpy= 0.574790

Thermal correction to Gibbs Free Energy= 0.497247

Sum of electronic and zero-point Energies= -951.381859

Sum of electronic and thermal Energies= -951.355902

Sum of electronic and thermal Enthalpies= -951.354958

Sum of electronic and thermal Free Energies= -951.432501

The wave number of the imaginary frequency= -408.7226 cm^-1^

Entry **10** (TS1)

C 2.873808 0.007246 -0.689388

C 2.013264 1.255213 -0.848619

C 0.715127 1.303401 -0.008659

N -0.020406 0.006410 -0.186206

C 0.712081 -1.291805 -0.021132

C 2.002126 -1.236022 -0.870984

H 3.634315 0.013113 -1.488344

O 3.541838 0.064489 0.576724

H 2.613533 2.142231 -0.615254

H 1.740631 1.320671 -1.910012

O -1.171838 -0.000043 0.578857

H 1.720479 -1.278119 -1.931844

H 2.589194 -2.144443 -0.672406

C -0.151272 2.426251 -0.609171

H -1.001730 2.657085 0.033449

H 0.448032 3.338845 -0.708629

H -0.520147 2.145978 -1.601472

C 0.999601 1.662966 1.468557

H 0.089483 1.530465 2.060280

H 1.802981 1.064141 1.895804

H 1.301996 2.715093 1.539252

C 1.006750 -1.656538 1.452965

H 1.337056 -2.701130 1.518758

H 1.786565 -1.031082 1.886844

H 0.093908 -1.552117 2.045612

C -0.159811 -2.410355 -0.620612

H 0.420622 -3.338546 -0.681480

H -1.035151 -2.605209 0.000700

H -0.492985 -2.144372 -1.628569

C -2.792185 -0.014289 -0.125178

C -2.716001 -0.160324 -1.539565

H -2.636775 -1.140399 -1.998557

H -2.584113 0.702306 -2.185116

C -3.363851 -1.192783 0.647696

C -3.333918 1.312565 0.383937

H -3.038411 -2.150737 0.235033

H -3.053656 -1.141924 1.696331

H -4.460275 -1.167574 0.614254

H -3.008973 1.484748 1.415001

H -3.006418 2.152847 -0.232114

H -4.431130 1.296254 0.371050

H 4.039497 -0.761720 0.681149

Zero-point correction= 0.376141 Hartree/Particle

Thermal correction to Energy= 0.394784

Thermal correction to Enthalpy= 0.395728

Thermal correction to Gibbs Free Energy= 0.331861

Sum of electronic and zero-point Energies= -715.721232

Sum of electronic and thermal Energies= -715.702589

Sum of electronic and thermal Enthalpies= -715.701645

Sum of electronic and thermal Free Energies= -715.765512

The wave number of the imaginary frequency= -630.9203 cm^-1^

Entry **10** (TS2)

C 1.637967 0.063407 1.281713

C 1.892734 1.574231 1.522113

C 2.953030 -0.746178 1.387983

N 0.973339 -0.266665 -0.040088

C 3.904743 -0.643708 0.205065

C 1.821321 -0.083038 -1.278056

C 3.127117 -0.895018 -1.083256

C 0.745910 -0.437405 2.428367

O -0.213150 0.514108 -0.125361

H 3.474833 -0.438918 2.301879

C 2.110860 1.387442 -1.661151

O 4.563338 0.628693 0.244025

H 2.696538 -1.807151 1.503890

C 1.122091 -0.735596 -2.483755

H 4.666933 -1.434438 0.308314

H 2.870945 -1.962830 -1.102749

H 3.772651 -0.718098 -1.955529

H 2.056383 1.745428 2.593061

H 2.775406 1.939783 1.001221

H 1.022775 2.159952 1.217183

H 1.301264 -0.369729 3.371007

H -0.150829 0.172819 2.527946

H 0.457167 -1.481674 2.282108

H 1.183266 1.964934 -1.643674

H 2.839159 1.861028 -1.005451

H 2.509563 1.423992 -2.682992

H 1.840645 -0.830224 -3.306856

H 0.752988 -1.734882 -2.237136

H 0.291370 -0.129184 -2.844079

C -1.845225 -0.024818 -0.461387

C -2.268523 -1.422101 0.183481

C -2.533767 1.329712 0.025887

C -1.849371 -0.137362 -1.901774

H -1.801668 0.733680 -2.539928

H -1.752931 -1.095460 -2.393466

C -1.193675 -2.502122 -0.095109

C -2.558967 -1.422617 1.699808

C -3.575699 -1.949654 -0.483209

H -4.440140 -1.311399 -0.294623

H -3.478470 -2.073358 -1.564826

H -3.800582 -2.936913 -0.060984

H -1.080681 -2.698934 -1.166374

H -0.214892 -2.219642 0.288825

H -1.508249 -3.444210 0.369812

H -1.723543 -1.067425 2.297982

H -3.443222 -0.831050 1.951507

H -2.769865 -2.452658 2.011643

C -2.202125 1.748546 1.476886

C -2.097067 2.524204 -0.867038

C -4.073815 1.238727 -0.102852

H -4.379809 0.900437 -1.098352

H -4.525606 0.577576 0.641933

H -4.499866 2.237219 0.054218

H -2.476407 1.001035 2.219891

H -1.142681 1.976410 1.590808

H -2.767964 2.658201 1.713727

H -1.011911 2.573209 -0.968145

H -2.545789 2.490948 -1.865031

H -2.439543 3.455357 -0.401564

H 5.122659 0.690509 -0.546190

Zero-point correction= 0.548121 Hartree/Particle

Thermal correction to Energy= 0.573925

Thermal correction to Enthalpy= 0.574869

Thermal correction to Gibbs Free Energy= 0.497834

Sum of electronic and zero-point Energies= -951.380416

Sum of electronic and thermal Energies= -951.354612

Sum of electronic and thermal Enthalpies= -951.353668

Sum of electronic and thermal Free Energies= -951.430703

The wave number of the imaginary frequency= -411.7443 cm^-1^

Entry **11** (TS1)

C -2.875310 0.011001 -0.104111

C -2.054173 -1.230172 -0.469194

C -0.658056 -1.297727 0.189571

N 0.050786 -0.000209 -0.061544

C -0.657345 1.298050 0.194066

C -2.052344 1.243604 -0.465623

N -4.189541 0.092645 -0.759976

H -3.077523 0.013245 0.974437

H -2.602976 -2.144364 -0.203803

H -1.928084 -1.240905 -1.562092

O 1.287977 -0.002860 0.555556

H -1.922059 1.258009 -1.558327

H -2.606679 2.150779 -0.199677

C 0.127223 -2.417443 -0.516102

H 1.065557 -2.630310 -0.001748

H -0.467316 -3.338502 -0.521695

H 0.345299 -2.140803 -1.552358

C -0.765995 -1.664712 1.688714

H 0.208578 -1.544480 2.169554

H -1.492151 -1.052271 2.228799

H -1.074555 -2.711565 1.796711

C -0.764374 1.659106 1.694432

H -1.069148 2.706499 1.805547

H -1.494921 1.048899 2.231112

H 0.209084 1.532681 2.176008

C 0.131374 2.418752 -0.506433

H -0.464464 3.338718 -0.514402

H 1.066617 2.631968 0.013075

H 0.354921 2.143445 -1.541975

C 2.807546 -0.003165 -0.350536

C 2.541588 -0.014658 -1.749055

H 2.353724 0.907187 -2.290074

H 2.363027 -0.946070 -2.276633

C 3.441211 1.262093 0.206869

C 3.446253 -1.256679 0.227302

H 3.053962 2.162313 -0.275643

H 3.257345 1.334943 1.283603

H 4.526668 1.237703 0.047837

H 3.263738 -1.311568 1.305332

H 3.061109 -2.166436 -0.239015

H 4.531432 -1.231747 0.066620

H -4.060146 0.053509 -1.772430

H -4.739150 -0.733356 -0.521965

Zero-point correction= 0.389118 Hartree/Particle

Thermal correction to Energy= 0.407957

Thermal correction to Enthalpy= 0.408901

Thermal correction to Gibbs Free Energy= 0.344236

Sum of electronic and zero-point Energies= -695.844290

Sum of electronic and thermal Energies= -695.825452

Sum of electronic and thermal Enthalpies= -695.824507

Sum of electronic and thermal Free Energies= -695.889173

The wave number of the imaginary frequency= -629.3420 cm^-1^

Entry **11** (TS2)

C 3.909655 0.101350 0.122741

C 2.996951 0.660958 1.215265

C 1.605290 -0.006583 1.292467

N 0.958449 0.076387 -0.074111

C 1.772226 -0.449854 -1.237163

C 3.168881 0.217275 -1.202391

N 5.219338 0.761123 0.011294

H 4.122235 -0.955173 0.327661

H 3.474135 0.564069 2.200504

H 2.862341 1.737171 1.030715

O -0.295048 -0.591036 0.009430

H 3.046967 1.286813 -1.431808

H 3.779044 -0.210148 -2.005923

C 0.778784 0.813099 2.293639

H -0.175437 0.333376 2.508510

H 1.331790 0.894148 3.236668

H 0.597771 1.824995 1.921338

C 1.726942 -1.444135 1.862541

H 0.802919 -2.000968 1.694062

H 2.550980 -2.014370 1.429301

H 1.902305 -1.395951 2.943713

C 1.895520 -1.990529 -1.297376

H 2.265589 -2.288124 -2.285503

H 2.584598 -2.402484 -0.557654

H 0.915782 -2.451174 -1.149725

C 1.134259 0.008905 -2.559270

H 1.847448 -0.153861 -3.375788

H 0.232724 -0.557165 -2.793470

H 0.886769 1.073524 -2.529585

C -1.876502 0.004686 -0.461726

C -1.881754 -0.210380 -1.889891

H -1.916318 -1.203639 -2.314573

H -1.718715 0.600718 -2.585803

C -2.680654 -1.138255 0.308032

C -2.164228 1.543130 -0.149752

C -2.368075 -2.536497 -0.292904

C -2.372343 -1.255298 1.819247

C -4.207397 -0.935408 0.149113

C -2.430001 1.907643 1.326482

C -1.000290 2.430863 -0.657752

C -3.428218 2.024128 -0.924986

H 5.077138 1.761198 -0.139636

H 5.714070 0.685237 0.900372

H -2.824738 -2.684689 -1.276893

H -1.293482 -2.707891 -0.372515

H -2.788698 -3.304842 0.365714

H -1.342268 -1.567584 1.989955

H -2.549126 -0.334969 2.373860

H -3.029726 -2.021016 2.249586

H -4.587547 -0.090182 0.729471

H -4.493118 -0.793462 -0.898442

H -4.721458 -1.832730 0.514520

H -3.355977 1.464540 1.702569

H -1.616815 1.625777 1.990571

H -2.548998 2.995350 1.398690

H -3.340238 1.882687 -2.005220

H -4.344621 1.532726 -0.595222

H -3.551731 3.099511 -0.745938

H -0.040942 2.137223 -0.234373

H -0.903489 2.391571 -1.747893

H -1.209580 3.474916 -0.395434

Zero-point correction= 0.560918 Hartree/Particle

Thermal correction to Energy= 0.586951

Thermal correction to Enthalpy= 0.587895

Thermal correction to Gibbs Free Energy= 0.510275

Sum of electronic and zero-point Energies= -931.503515

Sum of electronic and thermal Energies= -931.477482

Sum of electronic and thermal Enthalpies= -931.476538

Sum of electronic and thermal Free Energies= -931.554158

The wave number of the imaginary frequency= -413.4870 cm^-1^

Entry **12** (TS1)

C -2.866309 -0.005209 -0.458684

C -2.072325 -1.258858 -0.746738

C -0.736196 -1.299456 0.041864

N -0.017532 0.005580 -0.158589

C -0.748128 1.302237 0.048131

C -2.079355 1.252662 -0.747989

O -4.001949 -0.008042 -0.021176

H -2.672750 -2.140324 -0.504747

H -1.845813 -1.277046 -1.821868

O 1.156817 0.007832 0.571643

H -1.846965 1.270624 -1.821833

H -2.686241 2.130755 -0.509873

C 0.120209 -2.418365 -0.572403

H 1.014302 -2.603774 0.024558

H -0.459392 -3.347681 -0.607525

H 0.421216 -2.158583 -1.591923

C -1.005148 -1.644228 1.524301

H -1.822065 -1.058722 1.953369

H -1.280914 -2.701099 1.616857

H -0.100865 -1.473010 2.114163

C -1.030308 1.633324 1.531500

H -1.845351 1.038092 1.950697

H -0.128570 1.465304 2.126305

H -1.315459 2.687209 1.630029

C 0.100757 2.435709 -0.549712

H 0.967165 2.651753 0.076341

H 0.444170 2.173542 -1.555706

H -0.501050 3.349120 -0.616200

C 2.765535 -0.006582 -0.188047

C 2.633981 -0.153059 -1.596955

H 2.542493 -1.133232 -2.053358

H 2.484585 0.709406 -2.238900

C 3.317188 1.321729 0.304048

C 3.358676 -1.184484 0.567865

H 3.025389 1.492661 1.345157

H 4.413383 1.308318 0.256887

H 2.968553 2.161119 -0.301459

H 3.078870 -1.134525 1.624987

H 3.024039 -2.142795 0.163854

H 4.453586 -1.157007 0.503505

Zero-point correction= 0.352261 Hartree/Particle

Thermal correction to Energy= 0.370641

Thermal correction to Enthalpy= 0.371585

Thermal correction to Gibbs Free Energy= 0.307344

Sum of electronic and zero-point Energies= -714.548764

Sum of electronic and thermal Energies= -714.530384

Sum of electronic and thermal Enthalpies= -714.529440

Sum of electronic and thermal Free Energies= -714.593681

The wave number of the imaginary frequency= -613.4750 cm^-1^

Entry **12** (TS2)

C -3.900001 -0.436681 0.061086

C -3.213112 -0.011249 1.334922

C -1.847713 0.693747 1.092929

N -1.017120 -0.168224 0.164212

C -1.674432 -0.598847 -1.132351

C -3.014017 -1.306528 -0.795547

O -5.027008 -0.097456 -0.249446

H -3.869889 0.653394 1.903037

H -3.035792 -0.914578 1.935539

O 0.188187 0.503323 -0.185749

H -2.795853 -2.232104 -0.245249

H -3.534586 -1.566728 -1.721671

C -1.174164 0.761384 2.472610

H -0.310139 1.425338 2.466995

H -1.890575 1.157227 3.201632

H -0.858779 -0.231487 2.805034

C -2.080911 2.143237 0.608871

H -2.833912 2.219896 -0.177838

H -2.429648 2.755763 1.448339

H -1.144838 2.571282 0.243078

C -1.941829 0.549508 -2.137978

H -2.835707 1.128438 -1.895681

H -1.087145 1.227443 -2.181865

H -2.096468 0.130772 -3.138889

C -0.793666 -1.642639 -1.829801

H 0.114420 -1.190426 -2.225623

H -0.526951 -2.454273 -1.147905

H -1.347726 -2.074906 -2.670493

C 1.813166 0.208097 0.407460

C 1.812743 0.893982 1.679096

H 1.708144 0.356451 2.611543

H 1.782775 1.972454 1.742928

C 2.206827 -1.322613 0.624186

C 2.524002 1.071848 -0.729485

C 3.505681 -1.427555 1.480324

C 1.112155 -2.060741 1.434425

C 2.493347 -2.147567 -0.649272

C 2.181408 0.654758 -2.178754

C 2.130253 2.570715 -0.617635

C 4.063284 1.022699 -0.569754

H 4.374926 1.260120 0.452760

H 4.490245 0.054903 -0.846768

H 4.509272 1.772353 -1.234484

H 1.048670 2.700082 -0.554288

H 2.597660 3.065855 0.239624

H 2.483862 3.094948 -1.512554

H 2.399322 -0.390109 -2.394686

H 1.132395 0.843331 -2.406459

H 2.787025 1.258136 -2.866126

H 4.383686 -1.019430 0.977656

H 3.413399 -0.933266 2.450929

H 3.703791 -2.489085 1.673085

H 1.008217 -1.653202 2.445425

H 0.135095 -2.003167 0.957827

H 1.400689 -3.112941 1.543782

H 1.657822 -2.172388 -1.344709

H 3.378320 -1.790411 -1.182478

H 2.700549 -3.182380 -0.351369

Zero-point correction= 0.523942 Hartree/Particle

Thermal correction to Energy= 0.549546

Thermal correction to Enthalpy= 0.550491

Thermal correction to Gibbs Free Energy= 0.473112

Sum of electronic and zero-point Energies= -950.208441

Sum of electronic and thermal Energies= -950.182837

Sum of electronic and thermal Enthalpies= -950.181893

Sum of electronic and thermal Free Energies= -950.259271

The wave number of the imaginary frequency= -391.1720 cm^-1^

Entry **13** (TS1)

C -0.820618 0.935731 0.057695

N 0.077850 -0.275308 0.181289

C -0.481861 -1.662326 -0.013355

C -1.786288 -1.804738 0.812223

C -2.743321 -0.667697 0.540943

C -2.130265 0.677205 0.851846

O -3.870004 -0.817495 0.105475

O 1.184662 -0.119742 -0.638127

H -1.532141 -1.792316 1.881043

H -2.267226 -2.759228 0.580763

H -1.912212 0.693379 1.926454

H -2.853691 1.468407 0.640080

C 0.529719 -2.668647 0.559373

H 0.078531 -3.667087 0.585718

H 1.427819 -2.724837 -0.059175

H 0.817852 -2.389991 1.577291

C -0.754647 -2.030067 -1.490044

H -1.659628 -1.560128 -1.882918

H 0.094660 -1.734263 -2.110337

H -0.887267 -3.114323 -1.581852

C -0.100520 2.150096 0.694358

H 0.757069 2.399075 0.064802

H -0.779081 3.008283 0.632620

C 0.350018 1.982902 2.150491

H 1.032684 2.792455 2.434564

H -0.492007 2.019946 2.850819

H 0.862635 1.028564 2.295992

C -1.160583 1.245839 -1.433526

H -1.845570 0.476825 -1.803551

H -0.232801 1.142277 -2.004553

C -1.787843 2.614501 -1.738164

H -2.697568 2.799204 -1.154493

H -1.095919 3.442307 -1.552908

H -2.071811 2.659232 -2.795861

C 3.183662 1.444007 -0.258665

C 2.855561 -0.029452 -0.079161

C 2.998128 -0.534445 1.246043

C 3.439893 -0.890310 -1.188453

H 2.814819 2.048895 0.573019

H 4.272321 1.574844 -0.307672

H 2.906242 0.116961 2.108665

H 2.756150 1.823849 -1.191581

H 3.118197 -1.595542 1.433738

H 4.529664 -0.770645 -1.221741

H 3.031613 -0.586172 -2.157340

H 3.218228 -1.950518 -1.039021

Zero-point correction= 0.410012 Hartree/Particle

Thermal correction to Energy= 0.430961

Thermal correction to Enthalpy= 0.431905

Thermal correction to Gibbs Free Energy= 0.362576

Sum of electronic and zero-point Energies= -793.109971

Sum of electronic and thermal Energies= -793.089023

Sum of electronic and thermal Enthalpies= -793.088078

Sum of electronic and thermal Free Energies= -793.157407

The wave number of the imaginary frequency= -591.0988 cm^-1^

Entry **13** (TS2)

C -1.231621 -1.715461 0.067798

N -0.747519 -0.333280 -0.332821

C -1.750588 0.809595 -0.209983

C -3.087906 0.365930 -0.879465

C -3.563633 -1.018970 -0.523409

C -2.496884 -2.053742 -0.762914

O -4.670367 -1.266803 -0.079507

O 0.400110 -0.058365 0.468121

H -2.922945 0.361411 -1.966526

H -3.879284 1.085206 -0.670208

H -2.231946 -2.060226 -1.828543

H -2.867404 -3.044493 -0.485548

C -1.222119 1.974608 -1.083639

C -2.123919 3.211717 -1.195035

H -0.264184 2.291078 -0.670133

H -1.018278 1.582652 -2.087370

C -1.922661 1.309548 1.271346

C -3.342140 1.634761 1.759700

H -1.485111 0.572885 1.943528

H -1.300153 2.203549 1.396146

C -0.172439 -2.759148 -0.303834

H 0.707512 -2.671969 0.331224

H -0.591020 -3.761804 -0.161912

H 0.126695 -2.663450 -1.350906

C -1.569946 -1.888799 1.568748

H -2.477193 -1.356120 1.865577

H -0.742853 -1.551035 2.196754

H -1.742142 -2.950907 1.778197

C 2.562962 -0.200891 -1.361088

C 1.998271 0.471336 -0.030870

C 1.821880 1.891060 -0.239543

C 2.675657 0.168007 1.380706

C 1.506706 -0.132347 -2.491772

C 3.798761 0.590036 -1.891209

H 1.785852 2.320005 -1.231560

C 3.041944 -1.663722 -1.244023

H 1.623831 2.565209 0.581609

C 4.190571 0.485457 1.333920

C 2.511329 -1.281647 1.893773

C 2.077779 1.078914 2.488693

H 2.411204 2.118219 2.402509

H 0.986919 1.056086 2.483209

H 2.423269 0.719914 3.464677

H 1.468032 -1.507589 2.113049

H 2.891529 -2.035326 1.205340

H 3.078695 -1.384830 2.826800

H 4.759480 -0.240098 0.745965

H 4.385565 1.486118 0.933950

H 4.588294 0.454442 2.355429

H 3.924297 -1.760952 -0.605410

H 2.274150 -2.340145 -0.877320

H 3.334781 -2.012466 -2.241510

H 0.572360 -0.617717 -2.214158

H 1.269046 0.901095 -2.765268

H 1.913557 -0.615511 -3.388138

H 3.575265 1.639490 -2.099360

H 4.652540 0.556793 -1.212679

H 4.115508 0.133359 -2.836850

H -3.808013 2.443926 1.186505

H -4.010565 0.768859 1.706207

H -3.304249 1.959898 2.806222

H -3.086590 2.993198 -1.669773

H -2.326142 3.664914 -0.218508

H -1.627662 3.973177 -1.807772

Zero-point correction= 0.582237 Hartree/Particle

Thermal correction to Energy= 0.610152

Thermal correction to Enthalpy= 0.611096

Thermal correction to Gibbs Free Energy= 0.528653

Sum of electronic and zero-point Energies= -1028.765303

Sum of electronic and thermal Energies= -1028.737388

Sum of electronic and thermal Enthalpies= -1028.736444

Sum of electronic and thermal Free Energies= -1028.818887

The wave number of the imaginary frequency= -399.2783 cm^-1^

Entry **14** (TS1)

C 0.875781 -1.140842 0.256087

N -0.130944 -0.027027 0.168868

C 0.198488 1.454697 0.113159

C 1.712167 1.761745 0.396537

C 2.657256 0.587443 0.303763

C 2.117169 -0.620748 1.017708

O 3.723161 0.622905 -0.283307

O -1.134727 -0.361033 -0.717011

H 1.812169 2.127291 1.428586

H 2.074366 2.559055 -0.256213

H 1.825836 -0.326507 2.032364

H 2.873751 -1.404742 1.086168

C -0.653906 2.100727 1.247412

C -0.574262 3.625208 1.409701

H -1.699001 1.818291 1.087193

H -0.352388 1.620208 2.187380

C -0.178517 2.109843 -1.243222

C 0.508176 1.580096 -2.504326

H -1.260916 2.051635 -1.377219

H 0.059780 3.175547 -1.141177

C 0.244763 -2.311554 1.054145

H -0.675529 -2.595701 0.536195

H 0.914330 -3.175972 0.977852

C -0.053640 -2.044242 2.532842

H -0.670776 -2.848860 2.949596

H 0.859506 -1.997852 3.136818

H -0.596225 -1.102019 2.659714

C 1.286299 -1.643951 -1.161803

H 1.559498 -0.776651 -1.768859

H 0.392289 -2.069946 -1.627216

C 2.442652 -2.651987 -1.240535

H 3.393284 -2.203029 -0.935086

H 2.273039 -3.546248 -0.630632

H 2.562937 -2.988160 -2.277001

C -2.978451 -1.931920 -1.100045

C -2.768746 -0.672664 -0.270199

C -2.928685 -0.833390 1.143243

C -3.484938 0.529045 -0.872149

H -2.491157 -2.800952 -0.648503

H -4.049183 -2.151405 -1.191768

H -2.791229 -1.798363 1.618280

H -2.569883 -1.792795 -2.105973

H -3.087147 0.021352 1.792989

H -4.564385 0.337483 -0.915598

H -3.131648 0.712852 -1.891483

H -3.331264 1.436479 -0.281241

H 0.456288 3.978555 1.532498

H -1.008192 4.155417 0.555924

H -1.131050 3.934693 2.301722

H 1.600854 1.571547 -2.412833

H 0.171952 0.567486 -2.736872

H 0.259596 2.220494 -3.359045

Zero-point correction= 0.467492 Hartree/Particle

Thermal correction to Energy= 0.491062

Thermal correction to Enthalpy= 0.492007

Thermal correction to Gibbs Free Energy= 0.416912

Sum of electronic and zero-point Energies= -871.666745

Sum of electronic and thermal Energies= -871.643175

Sum of electronic and thermal Enthalpies= -871.642230

Sum of electronic and thermal Free Energies= -871.717325

The wave number of the imaginary frequency= -587.5837 cm^-1^

Entry **14** (TS2)

C 1.320827 1.400100 -0.037429

N 0.660048 0.090258 -0.506274

C 1.504703 -1.193298 -0.405904

C 2.850037 -0.942601 -1.133613

C 3.572933 0.218311 -0.509674

C 2.767187 1.482651 -0.613512

O 4.664371 0.139777 0.026133

O -0.531442 -0.071571 0.285389

H 2.649628 -0.713229 -2.188551

H 3.477038 -1.834222 -1.084289

H 2.682327 1.702204 -1.687036

H 3.311856 2.305289 -0.151689

C 0.740652 -2.295719 -1.190951

C 1.551253 -3.402291 -1.889012

H 0.024355 -2.769856 -0.517047

H 0.158162 -1.780258 -1.959403

C 1.787618 -1.614980 1.066183

H 2.491641 -0.905197 1.513239

H 0.851390 -1.516078 1.620254

C 2.350767 -3.023414 1.306883

C 0.566403 2.579743 -0.704105

H -0.482504 2.512023 -0.434038

H 0.620264 2.447592 -1.790203

C 1.058419 3.992692 -0.356630

C 1.272651 1.568479 1.526010

H 1.077232 0.600299 1.981207

H 0.390186 2.172315 1.767412

C 2.495140 2.184068 2.225161

C -2.768814 0.855547 -0.956687

C -2.153294 -0.410647 -0.224710

C -2.150385 -1.522897 -1.148499

C -2.673697 -0.836185 1.223788

C -1.917198 1.158092 -2.227384

C -4.199595 0.574116 -1.504805

H -2.097918 -1.361164 -2.217019

C -2.905105 2.107769 -0.062268

H -2.135187 -2.551332 -0.821848

C -4.215383 -0.981038 1.244717

C -2.285813 0.128388 2.372498

C -2.116518 -2.228202 1.624472

H -2.594273 -3.047278 1.076870

H -1.037919 -2.293942 1.481158

H -2.324917 -2.400762 2.686123

H -1.205819 0.190497 2.502308

H -2.672594 1.137584 2.235538

H -2.719305 -0.256834 3.303762

H -4.731700 -0.017444 1.237764

H -4.586163 -1.581514 0.407507

H -4.505607 -1.490617 2.171445

H -3.751151 2.012394 0.625356

H -2.018409 2.308489 0.537128

H -3.100999 2.986539 -0.687717

H -0.864431 0.915173 -2.095671

H -2.285951 0.588599 -3.087732

H -2.002305 2.217008 -2.494070

H -4.236573 -0.339606 -2.106075

H -4.959924 0.500842 -0.726716

H -4.483273 1.409125 -2.157937

H 2.307152 2.235370 3.304328

H 3.401945 1.587882 2.077013

H 2.710929 3.203198 1.886919

H 0.449294 4.729749 -0.892518

H 0.966161 4.213844 0.711675

H 2.100527 4.163570 -0.647104

H 2.533719 -3.160124 2.378980

H 1.656281 -3.810075 0.994583

H 3.305989 -3.187944 0.795965

H 0.855790 -4.119405 -2.341164

H 2.177531 -3.011115 -2.697260

H 2.197025 -3.961145 -1.206881

Zero-point correction= 0.640427 Hartree/Particle

Thermal correction to Energy= 0.670917

Thermal correction to Enthalpy= 0.671862

Thermal correction to Gibbs Free Energy= 0.583571

Sum of electronic and zero-point Energies= -1107.317751

Sum of electronic and thermal Energies= -1107.287260

Sum of electronic and thermal Enthalpies= -1107.286316

Sum of electronic and thermal Free Energies= -1107.374607

The wave number of the imaginary frequency= -385.6686 cm^-1^

Entry **15** (TS1)

C 1.350584 -0.318927 0.100554

C 1.368919 -1.033985 1.483256

C 0.241167 -2.037989 1.545203

C -1.129274 -1.423742 1.344923

C -1.255760 -0.524482 0.070301

N -0.008131 0.344467 -0.090543

C 2.427684 0.788393 0.051106

C 3.875574 0.275394 0.051780

C 1.653787 -1.326449 -1.041834

C 3.112187 -1.824158 -1.079643

C 4.129041 -0.681603 -1.115977

C -1.497426 -1.360359 -1.219761

C -2.857695 -2.082171 -1.270818

C -4.036315 -1.131242 -1.049945

C -3.834108 -0.340144 0.245563

C -2.486406 0.399822 0.248729

O 0.413682 -3.231836 1.720956

O -0.094073 1.434359 0.756102

H 1.219323 -0.265547 2.252521

H 2.315418 -1.544565 1.672692

H -1.318855 -0.782511 2.217549

H -1.873088 -2.223131 1.348800

H 2.273502 1.478659 0.881310

H 2.264268 1.359497 -0.871867

H 4.101430 -0.231882 0.999944

H 4.552667 1.137643 -0.000438

H 1.405313 -0.842463 -1.994950

H 1.025133 -2.213602 -0.955757

H 3.306236 -2.457915 -0.203910

H 3.229926 -2.475197 -1.955071

H 4.031467 -0.132268 -2.064174

H 5.152490 -1.076220 -1.083493

H -0.724100 -2.112564 -1.364213

H -1.423735 -0.668644 -2.069074

H -2.885699 -2.879435 -0.515526

H -2.945013 -2.585131 -2.242248

H -4.981859 -1.687546 -1.024074

H -4.104925 -0.430796 -1.895491

H -3.900722 -1.018705 1.106922

H -4.637089 0.396613 0.376518

H -2.377032 0.966070 1.174054

H -2.491891 1.122765 -0.574078

C -1.281445 3.622247 0.966997

C -0.223813 3.087349 0.016574

C 1.069377 3.660534 0.206208

C -0.636749 2.873136 -1.426784

H -0.953736 3.509574 2.005701

H -2.235568 3.106575 0.846112

H 1.376036 4.025313 1.182318

H -1.445721 4.690578 0.777399

H 1.819001 3.644705 -0.577568

H -0.336354 1.864644 -1.744128

H -0.153920 3.600726 -2.087096

H -1.718819 2.963558 -1.559946

Zero-point correction= 0.485809 Hartree/Particle

Thermal correction to Energy= 0.507625

Thermal correction to Enthalpy= 0.508569

Thermal correction to Gibbs Free Energy= 0.436563

Sum of electronic and zero-point Energies= -947.876184

Sum of electronic and thermal Energies= -947.854368

Sum of electronic and thermal Enthalpies= -947.853424

Sum of electronic and thermal Free Energies= -947.925430

The wave number of the imaginary frequency= -515.7675 cm^-1^

Entry **15** (TS2)

C 1.170855 1.486735 -0.160050

N 0.613504 0.102770 -0.549645

C 1.566188 -1.087894 -0.426392

C 2.852033 -0.748106 -1.224767

C 3.498598 0.490688 -0.671371

C 2.586527 1.682293 -0.781975

O 4.612465 0.524187 -0.180187

O -0.558771 -0.132105 0.245771

H 2.573395 -0.569696 -2.271800

H 3.558738 -1.578282 -1.189834

H 2.466107 1.883723 -1.855939

H 3.073508 2.550671 -0.336010

C 0.913112 -2.297426 -1.125011

C 1.705438 -3.610429 -1.019813

H -0.058086 -2.466011 -0.660536

H 0.723639 -2.040272 -2.174165

C 1.936630 -1.475631 1.033909

H 2.569689 -0.705998 1.482581

H 1.012744 -1.504707 1.619008

C 2.681046 -2.819910 1.165724

C 0.278211 2.576598 -0.798868

H -0.751461 2.384139 -0.515516

H 0.330467 2.484619 -1.888848

C 0.625649 4.016793 -0.383444

H -0.088040 4.700322 -0.861389

C 0.588773 4.182313 1.139496

H 1.617453 4.302275 -0.760231

C 1.214312 1.719258 1.380133

H 1.947464 1.060727 1.850586

H 0.235415 1.436130 1.775822

C 1.522121 3.167334 1.806518

H 2.564420 3.422949 1.570005

H 1.439823 3.232354 2.899206

C -2.858431 0.553580 -1.068989

C -2.149094 -0.624041 -0.271405

C -2.047012 -1.766238 -1.153569

C -2.658013 -1.034225 1.182794

C -2.004189 0.890402 -2.329055

C -4.238095 0.110803 -1.643480

H -1.862368 -1.636750 -2.211785

C -3.153338 1.816964 -0.230094

H -2.121723 -2.785659 -0.802675

C -4.182793 -1.303909 1.187150

C -2.365500 -0.000663 2.299035

C -1.991028 -2.355464 1.650431

H -2.378006 -3.237169 1.128777

H -0.907619 -2.326057 1.532065

H -2.208848 -2.504643 2.713933

H -1.295207 0.103682 2.476590

H -2.778923 0.986838 2.097791

H -2.826787 -0.359504 3.227430

H -4.774654 -0.386925 1.123535

H -4.487046 -1.971958 0.374778

H -4.449494 -1.791119 2.132749

H -3.991906 1.648719 0.452318

H -2.307927 2.153190 0.367991

H -3.444204 2.637024 -0.897145

H -0.937754 0.747311 -2.165653

H -2.297809 0.260808 -3.176412

H -2.177129 1.927873 -2.634556

H -4.162730 -0.818776 -2.216232

H -5.009124 -0.017895 -0.883175

H -4.586366 0.893124 -2.329731

H -0.439581 4.022653 1.496659

H 0.864958 5.205407 1.424976

H 3.699416 -2.717292 0.767358

H 2.798775 -3.051247 2.232210

H 2.658264 -3.542289 -1.562275

C 1.970028 -3.971523 0.446695

H 1.133625 -4.407074 -1.513279

H 1.012201 -4.180896 0.945433

H 2.566444 -4.890178 0.516616

Zero-point correction= 0.657602 Hartree/Particle

Thermal correction to Energy= 0.686670

Thermal correction to Enthalpy= 0.687614

Thermal correction to Gibbs Free Energy= 0.601256

Sum of electronic and zero-point Energies= -1183.532715

Sum of electronic and thermal Energies= -1183.503647

Sum of electronic and thermal Enthalpies= -1183.502703

Sum of electronic and thermal Free Energies= -1183.589061

The wave number of the imaginary frequency= -405.1781 cm^-1^

Entry **16** (TS1)

C 1.308686 -0.404783 0.105896

C 1.291795 -1.148522 1.475789

C 0.067724 -2.031222 1.574614

C -1.233295 -1.271616 1.422524

C -1.288703 -0.460371 0.087790

N -0.003708 0.335414 -0.085313

C 2.450345 0.635979 0.078469

C 3.842745 0.009898 -0.012776

C 1.582137 -1.387973 -1.063973

C 3.040283 -1.868742 -1.126056

O 3.975876 -0.806108 -1.165720

C -1.540464 -1.384491 -1.134926

C -2.972887 -1.935195 -1.199564

O -3.960026 -0.920143 -1.156478

C -3.842177 -0.168748 0.040548

C -2.480600 0.523050 0.142784

O 0.121356 -3.239695 1.722686

O -0.029753 1.418659 0.778849

H 1.247823 -0.384527 2.262618

H 2.186279 -1.757283 1.626046

H -1.303793 -0.550353 2.247646

H -2.064831 -1.975020 1.506567

H 2.392353 1.282726 0.956193

H 2.312124 1.264392 -0.807580

H 4.079608 -0.574282 0.893007

H 4.598879 0.796089 -0.103545

H 1.330476 -0.883593 -2.003291

H 0.968518 -2.288118 -0.991201

H 3.261756 -2.532646 -0.273088

H 3.193374 -2.448991 -2.041136

H -0.876599 -2.250184 -1.141534

H -1.340185 -0.805436 -2.043271

H -3.144520 -2.660498 -0.385878

H -3.116765 -2.465228 -2.146072

H -4.030058 -0.815999 0.914302

H -4.637509 0.582619 0.011672

H -2.438921 1.112460 1.060206

H -2.401545 1.213811 -0.702447

C -1.241021 3.660389 0.733303

C -0.004287 3.099235 0.050997

C 1.222975 3.630183 0.537509

C -0.107278 2.918166 -1.447091

H -1.197929 3.478798 1.811919

H -2.160497 3.222115 0.342779

H 1.296004 4.025813 1.546582

H -1.291702 4.744956 0.574179

H 2.138098 3.566534 -0.040429

H 0.867420 2.718054 -1.901028

H -0.507986 3.834191 -1.898797

H -0.769406 2.088585 -1.706954

Zero-point correction= 0.437569 Hartree/Particle

Thermal correction to Energy= 0.459007

Thermal correction to Enthalpy= 0.459951

Thermal correction to Gibbs Free Energy= 0.388463

Sum of electronic and zero-point Energies= -1019.707644

Sum of electronic and thermal Energies= -1019.686206

Sum of electronic and thermal Enthalpies= -1019.685262

Sum of electronic and thermal Free Energies= -1019.756749

The wave number of the imaginary frequency= -554.9833 cm^-1^

Entry **16** (TS2)

C 1.289626 1.417733 -0.186703

N 0.642368 0.086346 -0.596994

C 1.515005 -1.161086 -0.463905

C 2.857809 -0.914858 -1.202080

C 3.545746 0.312514 -0.668044

C 2.690982 1.538027 -0.851699

O 4.636100 0.310519 -0.126824

O -0.537094 -0.082976 0.197520

H 2.647895 -0.763215 -2.269047

H 3.514993 -1.780782 -1.098695

H 2.550051 1.681524 -1.931654

H 3.217810 2.410692 -0.461148

C 0.804624 -2.315504 -1.196245

C 1.417920 -3.691488 -0.925554

H -0.226566 -2.353408 -0.853243

H 0.781696 -2.113145 -2.274415

C 1.789281 -1.592613 1.005499

H 2.546302 -0.951387 1.466760

H 0.864290 -1.473013 1.573194

C 2.282957 -3.040375 1.139375

C 0.445536 2.584585 -0.743937

H -0.592287 2.417511 -0.484739

H 0.514149 2.606906 -1.837402

C 0.840434 3.954431 -0.181996

H 0.138077 4.715607 -0.536646

O 0.769905 3.974338 1.234570

H 1.848836 4.257110 -0.513041

C 1.400365 1.601947 1.355484

H 2.186231 0.968673 1.776166

H 0.451304 1.291336 1.797163

C 1.689936 3.045864 1.787099

H 2.720091 3.340478 1.521230

H 1.597119 3.131756 2.874345

C -2.815713 0.805244 -0.991399

C -2.172223 -0.477709 -0.315593

C -2.136055 -1.545764 -1.287409

C -2.662863 -0.977777 1.114817

C -1.989846 1.166696 -2.264691

C -4.247147 0.513023 -1.534279

H -1.953375 -1.341553 -2.334345

C -2.969235 2.020986 -0.048473

H -2.274012 -2.583546 -1.018625

C -4.204749 -1.122800 1.155702

C -2.256362 -0.070167 2.303396

C -2.101223 -2.388931 1.439194

H -2.608335 -3.182365 0.879787

H -1.029129 -2.475754 1.259287

H -2.277623 -2.600844 2.499732

H -1.178452 -0.077320 2.466148

H -2.578562 0.964178 2.190232

H -2.734788 -0.460865 3.209886

H -4.719280 -0.159651 1.206031

H -4.592351 -1.681279 0.297396

H -4.477991 -1.678284 2.060759

H -3.795437 1.872163 0.653525

H -2.078787 2.239722 0.539500

H -3.212795 2.911623 -0.639774

H -0.931514 0.931281 -2.165288

H -2.369064 0.625311 -3.138330

H -2.092633 2.233703 -2.488863

H -4.269625 -0.384543 -2.160694

H -4.999214 0.403930 -0.752577

H -4.552164 1.360433 -2.161061

H 3.318077 -3.135846 0.768447

H 2.284685 -3.337067 2.192869

H 2.433298 -3.780270 -1.349107

O 1.448686 -3.971097 0.465765

H 0.802901 -4.472360 -1.384402

Zero-point correction= 0.609768 Hartree/Particle

Thermal correction to Energy= 0.638138

Thermal correction to Enthalpy= 0.639082

Thermal correction to Gibbs Free Energy= 0.554418

Sum of electronic and zero-point Energies= -1255.364206

Sum of electronic and thermal Energies= -1255.335837

Sum of electronic and thermal Enthalpies= -1255.334892

Sum of electronic and thermal Free Energies= -1255.419556

The wave number of the imaginary frequency= -416.3797 cm^-1^

Entry **17** (TS1)

C 1.333724 -0.074873 0.652951

C 1.301582 -0.442231 2.167059

C 0.099051 -1.311946 2.457596

C -1.218386 -0.652797 2.103952

C -1.267949 -0.175632 0.616117

N 0.012693 0.571144 0.264833

C 2.457141 0.945632 0.369504

C 3.872679 0.376918 0.504095

C 1.625788 -1.326347 -0.218467

C 3.076591 -1.837638 -0.113962

N 4.050579 -0.787005 -0.355529

C -1.503463 -1.361832 -0.360663

C -2.909776 -1.976877 -0.276703

N -3.958085 -0.974267 -0.403728

C -3.835196 0.117811 0.549131

C -2.460377 0.793546 0.438105

O 0.180832 -2.446598 2.893891

O -0.042926 1.838476 0.819936

H 1.216087 0.496217 2.729329

H 2.207511 -0.962477 2.486818

H -1.330601 0.235837 2.739671

H -2.030585 -1.346037 2.335347

H 2.347675 1.805882 1.031323

H 2.332987 1.303797 -0.658059

H 4.088016 0.095668 1.545880

H 4.610814 1.120353 0.202171

H 1.405037 -1.078426 -1.262864

H 0.994785 -2.169426 0.065349

H 3.245721 -2.269501 0.884304

H 3.211752 -2.646965 -0.831916

C 5.058689 -0.799976 -1.292501

H -0.805805 -2.179802 -0.182482

H -1.330545 -0.998369 -1.380052

H -3.034230 -2.513893 0.675838

H -3.051547 -2.700157 -1.080176

C -4.967401 -1.150362 -1.323271

H -3.998722 -0.263191 1.569352

H -4.609254 0.863629 0.365245

H -2.396381 1.590940 1.179058

H -2.390050 1.250316 -0.553399

C -1.142694 4.057935 0.425435

C -0.070038 3.276478 -0.312792

C 1.239542 3.827662 -0.190584

C -0.429243 2.726919 -1.678981

H -0.871094 4.181737 1.478835

H -2.115755 3.566523 0.371372

H 1.516803 4.407960 0.684704

H -1.244276 5.056606 -0.017762

H 2.021124 3.605122 -0.908838

H -0.181919 1.656976 -1.718491

H 0.130244 3.237361 -2.469200

H -1.495661 2.838323 -1.896458

O -4.997816 -2.122057 -2.071665

C -6.055286 -0.083563 -1.385805

O 5.827458 0.147111 -1.427609

C 5.191120 -2.041796 -2.167255

H -6.593369 0.008894 -0.435520

H -5.651138 0.903029 -1.640079

H -6.758669 -0.386388 -2.161989

H 5.367741 -2.945853 -1.573699

H 4.297011 -2.210927 -2.778359

H 6.043021 -1.880496 -2.828459

Zero-point correction= 0.538578 Hartree/Particle

Thermal correction to Energy= 0.567307

Thermal correction to Enthalpy= 0.568251

Thermal correction to Gibbs Free Energy= 0.477925

Sum of electronic and zero-point Energies= -1285.206700

Sum of electronic and thermal Energies= -1285.177971

Sum of electronic and thermal Enthalpies= -1285.177027

Sum of electronic and thermal Free Energies= -1285.267354

The wave number of the imaginary frequency= -503.4327 cm^-1^

Entry **17** (TS2)

C 1.160153 -1.305280 -0.737737

N -0.113292 -0.479799 -0.846383

C -1.443034 -1.201505 -0.665931

C -1.446730 -2.494946 -1.532590

C -0.191492 -3.315926 -1.386594

C 1.038825 -2.509277 -1.709190

O -0.172317 -4.474248 -1.012296

O -0.000629 0.588410 0.096177

H -1.526185 -2.202897 -2.588521

H -2.312862 -3.113674 -1.290294

H 0.957223 -2.122993 -2.733291

H 1.922806 -3.147295 -1.649379

C -2.554150 -0.289191 -1.227230

C -3.976867 -0.802345 -0.973383

H -2.470813 0.685830 -0.748376

H -2.396194 -0.142339 -2.301581

C -1.785235 -1.531429 0.815032

H -1.146942 -2.331243 1.199524

H -1.585382 -0.634466 1.407061

C -3.246019 -1.965864 1.043874

C 2.334579 -0.424819 -1.207408

H 2.303816 0.496362 -0.632446

H 2.199228 -0.169252 -2.264614

C 3.718085 -1.054562 -1.003453

H 4.502948 -0.340191 -1.253525

N 3.905693 -1.435775 0.392360

H 3.855824 -1.940209 -1.641243

C 1.467694 -1.812179 0.703074

H 0.783736 -2.616587 0.986536

H 1.307856 -0.982150 1.396031

C 2.896555 -2.361370 0.885624

H 2.999626 -3.320777 0.356435

H 3.065770 -2.569630 1.942576

C 0.335089 2.891611 -1.542470

C -0.184585 2.309849 -0.147721

C -1.621926 2.487500 -0.051225

C 0.538190 2.721475 1.216471

C -0.173379 2.021402 -2.716761

C -0.251927 4.316938 -1.786048

H -2.228173 2.738124 -0.910728

C 1.862380 3.059684 -1.706352

H -2.156380 2.296313 0.868515

C 0.562363 4.263016 1.365734

C 1.987050 2.208470 1.381945

C -0.241819 2.184689 2.448468

H -1.191812 2.704501 2.607234

H -0.434204 1.113306 2.370736

H 0.362630 2.356274 3.346172

H 2.020985 1.121666 1.458346

H 2.659925 2.517541 0.583628

H 2.393297 2.617554 2.314999

H 1.262505 4.745690 0.678894

H -0.429348 4.703410 1.218949

H 0.888350 4.511721 2.382715

H 2.275365 3.806461 -1.023425

H 2.420312 2.134053 -1.583730

H 2.059147 3.419423 -2.723012

H 0.195905 0.998424 -2.663116

H -1.266389 1.973399 -2.745963

H 0.156139 2.472193 -3.660325

H -1.343553 4.331046 -1.827927

H 0.064949 5.044008 -1.037394

H 0.109451 4.671913 -2.758656

C 4.938704 -0.885921 1.117139

H -3.417703 -2.965550 0.616043

H -3.427093 -2.052808 2.115715

H -4.170814 -1.737604 -1.519118

N -4.191726 -1.030101 0.452508

H -4.712879 -0.067255 -1.299770

C -5.170112 -0.326528 1.116719

O 5.725208 -0.086377 0.618545

C 5.078829 -1.308642 2.575658

O -5.887382 0.486557 0.540813

C -5.341898 -0.595765 2.607965

H -5.568687 -1.648166 2.812157

H -4.445543 -0.329019 3.179746

H -6.173919 0.019218 2.952316

H 4.201477 -1.028384 3.169863

H 5.222575 -2.390097 2.678582

H 5.953325 -0.795374 2.976688

Zero-point correction= 0.709987 Hartree/Particle

Thermal correction to Energy= 0.746015

Thermal correction to Enthalpy= 0.746959

Thermal correction to Gibbs Free Energy= 0.642841

Sum of electronic and zero-point Energies= -1520.869092

Sum of electronic and thermal Energies= -1520.833064

Sum of electronic and thermal Enthalpies= -1520.832120

Sum of electronic and thermal Free Energies= -1520.936238

The wave number of the imaginary frequency= -341.7539 cm^-1^

Entry **18** (TS1)

C 1.331509 -0.095114 0.693493

C 1.310218 -0.658968 2.144813

C 0.178509 -1.649498 2.267327

C -1.181266 -1.055108 1.963867

C -1.270362 -0.226574 0.635323

N 0.002635 0.580746 0.405926

C 2.448218 0.962253 0.545169

C 3.873868 0.401276 0.639386

C 1.614856 -1.225724 -0.333355

C 3.049632 -1.781004 -0.271700

C 4.113508 -0.694488 -0.396679

C -1.548170 -1.123598 -0.606605

C -2.923452 -1.814792 -0.604573

C -4.073582 -0.830564 -0.405768

C -3.830486 0.028991 0.832865

C -2.471871 0.742711 0.763319

O 0.337688 -2.821072 2.563767

O -0.056813 1.776752 1.096792

H 1.140281 0.188040 2.821819

H 2.248141 -1.147881 2.417663

H -1.408686 -0.369999 2.793494

H -1.920328 -1.858984 1.987435

H 2.308765 1.746080 1.290310

H 2.333568 1.426869 -0.439813

H 4.081277 -0.013624 1.632995

H 4.598010 1.208680 0.478543

H 1.418905 -0.833192 -1.336998

H 0.947665 -2.074660 -0.175710

H 3.217270 -2.308695 0.674088

H 3.189745 -2.512264 -1.075566

O 4.068220 -0.159226 -1.729194

O 5.429282 -1.229681 -0.218155

H -0.794250 -1.900181 -0.724540

H -1.476540 -0.478398 -1.489100

H -2.985345 -2.572883 0.185287

H -3.067357 -2.342213 -1.554644

O -5.299943 -1.561306 -0.306696

O -4.207453 0.039903 -1.541803

H -3.901859 -0.617093 1.715414

H -4.631706 0.772571 0.911683

H -2.339495 1.374499 1.642435

H -2.488567 1.400051 -0.109283

C -1.267328 3.984391 0.818186

C -0.094051 3.317609 0.119820

C 1.166080 3.920422 0.388349

C -0.325062 2.894013 -1.314095

H -1.098944 4.012226 1.899727

H -2.208246 3.466062 0.627609

H 1.332660 4.473654 1.308309

H -1.372534 5.018431 0.466099

H 2.021484 3.759825 -0.258571

H -0.488230 3.786649 -1.931320

H -1.200323 2.249875 -1.417539

H 0.538116 2.355898 -1.715134

C -6.240237 -0.971293 -1.198172

C -5.340272 -0.394859 -2.286436

C 6.081589 -1.235003 -1.483583

C 5.407701 -0.070968 -2.202883

H 5.394702 -0.160711 -3.292818

H 5.867596 0.889463 -1.925303

H 5.913491 -2.183844 -2.014353

H 7.154931 -1.100791 -1.320769

H -6.820206 -0.180540 -0.699349

H -6.923334 -1.751217 -1.546757

H -5.761366 0.468558 -2.809285

H -5.062867 -1.163117 -3.023617

Zero-point correction= 0.574469 Hartree/Particle

Thermal correction to Energy= 0.603001

Thermal correction to Enthalpy= 0.603945

Thermal correction to Gibbs Free Energy= 0.515299

Sum of electronic and zero-point Energies= -1403.477696

Sum of electronic and thermal Energies= -1403.449163

Sum of electronic and thermal Enthalpies= -1403.448219

Sum of electronic and thermal Free Energies= -1403.536865

The wave number of the imaginary frequency= -571.0342 cm^-1^

Entry **18** (TS2)

C -1.253719 -1.155242 0.717856

N 0.067716 -0.409819 0.837930

C 1.356535 -1.206562 0.636901

C 1.274077 -2.554588 1.416570

C -0.052513 -3.263198 1.322484

C -1.197038 -2.358526 1.693393

O -0.182853 -4.421088 0.969225

O 0.015326 0.687612 -0.072214

H 1.437839 -2.346850 2.483659

H 2.064776 -3.231979 1.088761

H -1.040582 -1.973458 2.709476

H -2.129742 -2.924278 1.675266

C 2.500995 -0.396597 1.288051

C 3.901825 -0.986038 1.067659

H 2.493860 0.605193 0.861919

H 2.299104 -0.290707 2.359757

C 1.709466 -1.451175 -0.858912

H 1.002880 -2.146324 -1.317715

H 1.600701 -0.496138 -1.376736

C 3.130794 -1.988723 -1.104890

C -2.382290 -0.211856 1.182578

H -2.330878 0.685027 0.570066

H -2.201534 0.083183 2.222208

C -3.796975 -0.796235 1.048302

H -4.537600 -0.040927 1.336822

C -4.085928 -1.245508 -0.385361

H -3.951593 -1.654302 1.713001

C -1.582072 -1.645719 -0.723619

H -0.888340 -2.433104 -1.027519

H -1.437192 -0.806288 -1.408517

C -3.007803 -2.205384 -0.884595

H -3.116886 -3.146093 -0.333113

H -3.198599 -2.430805 -1.939961

C -0.160940 2.971175 1.612726

C 0.344256 2.386884 0.215617

C 1.790614 2.446358 0.151278

C -0.315886 2.884279 -1.151707

C 0.272110 2.041238 2.772061

C 0.505583 4.350800 1.904591

H 2.391398 2.658821 1.025240

C -1.678141 3.222667 1.752376

H 2.332836 2.162027 -0.739067

C -0.220954 4.427077 -1.256784

C -1.793367 2.485406 -1.365141

C 0.453614 2.324016 -2.378636

H 1.449783 2.763721 -2.488618

H 0.550724 1.238427 -2.332766

H -0.105972 2.576302 -3.286616

H -1.916709 1.409804 -1.479473

H -2.458460 2.820747 -0.570456

H -2.144144 2.956103 -2.292345

H -0.908068 4.942082 -0.579717

H 0.794668 4.788522 -1.063768

H -0.491209 4.727302 -2.276362

H -2.031146 4.013649 1.085574

H -2.279779 2.332872 1.582025

H -1.878468 3.558817 2.776736

H -0.130797 1.035298 2.663649

H 1.361152 1.951937 2.836841

H -0.074291 2.469270 3.720492

H 1.596307 4.299945 1.945250

H 0.232305 5.120137 1.181246

H 0.164020 4.692741 2.889363

O -4.140898 -0.118600 -1.274126

O -5.381661 -1.855195 -0.425004

H 3.237069 -3.020699 -0.748363

H 3.332524 -2.013109 -2.182417

H 4.008813 -1.969074 1.541596

C 4.208043 -1.148121 -0.420520

H 4.657274 -0.329952 1.515180

O 4.298973 0.159529 -1.012247

O 5.481011 -1.772414 -0.625304

C 6.381740 -0.796380 -1.136948

C 5.445512 0.169034 -1.856455

C -6.120375 -1.260091 -1.486510

C -5.510794 0.135818 -1.565758

H -5.979370 -1.811595 -2.427908

H -7.181140 -1.271698 -1.219552

H -5.571755 0.600333 -2.553887

H -5.959909 0.810342 -0.821263

H 5.200366 -0.190134 -2.867101

H 5.817646 1.195396 -1.920576

H 7.099804 -1.296394 -1.793535

H 6.926908 -0.292975 -0.324573

Zero-point correction= 0.746059 Hartree/Particle

Thermal correction to Energy= 0.781885

Thermal correction to Enthalpy= 0.782829

Thermal correction to Gibbs Free Energy= 0.680579

Sum of electronic and zero-point Energies= -1639.140403

Sum of electronic and thermal Energies= -1639.104577

Sum of electronic and thermal Enthalpies= -1639.103633

Sum of electronic and thermal Free Energies= -1639.205883

The wave number of the imaginary frequency= -381.5233 cm^-1^

Entry **19** (TS1)

C 0.165983 0.410123 -0.308740

C -0.043869 1.378738 -1.513370

C -0.841006 2.589677 -1.078465

C -2.196680 2.236346 -0.507516

C -2.085749 1.221237 0.676056

N -1.181767 0.059820 0.295348

C 0.831679 -0.899261 -0.782694

C 2.287628 -0.736399 -1.229154

C 1.106432 1.031369 0.758086

C 2.575818 1.135233 0.306550

N 3.094029 -0.136055 -0.172202

C -1.588959 1.937540 1.953821

C -3.500902 0.708283 0.982317

O -0.411581 3.728975 -1.135982

O -1.860070 -0.770235 -0.577965

H -0.608753 0.828539 -2.276556

H 0.902429 1.709163 -1.947976

H -2.789769 1.761914 -1.300619

H -2.707555 3.148953 -0.185760

H 0.248628 -1.336265 -1.594795

H 0.810500 -1.606399 0.053308

H 2.355948 -0.116714 -2.136104

H 2.726212 -1.707522 -1.459760

H 1.052226 0.413425 1.661783

H 0.800149 2.041995 1.028360

H 2.668191 1.892257 -0.487350

H 3.173366 1.489479 1.147028

C 4.242779 -0.763877 0.251270

C -3.627536 -2.532479 -0.684145

C -2.262051 -2.472735 -0.024416

C -1.270080 -3.283494 -0.642933

C -2.221961 -2.430171 1.489696

H -3.547107 -2.305142 -1.752070

H -4.330054 -1.830510 -0.230566

H -1.333296 -3.531259 -1.698510

H -4.046123 -3.541764 -0.582992

H -0.375288 -3.591153 -0.112226

H -1.478589 -1.691341 1.816514

H -1.940294 -3.406350 1.898191

H -3.189529 -2.152574 1.915693

O 4.610472 -1.832610 -0.227719

C 5.052885 -0.084886 1.350444

H 5.916389 -0.719328 1.552562

H 5.404558 0.907662 1.046801

H 4.475755 0.032154 2.274925

H -1.212716 1.212859 2.681727

H -0.817303 2.685622 1.767501

H -2.429826 2.468845 2.411849

H -3.502449 0.047075 1.853179

H -4.138245 1.568275 1.213953

H -3.930335 0.181243 0.132366

Zero-point correction= 0.445623 Hartree/Particle

Thermal correction to Energy= 0.469110

Thermal correction to Enthalpy= 0.470055

Thermal correction to Gibbs Free Energy= 0.392998

Sum of electronic and zero-point Energies= -999.876229

Sum of electronic and thermal Energies= -999.852742

Sum of electronic and thermal Enthalpies= -999.851798

Sum of electronic and thermal Free Energies= -999.928854

The wave number of the imaginary frequency= -518.2949 cm^-1^

Entry **19** (TS2)

C -1.204128 0.853621 -0.434291

N 0.208326 1.353216 -0.175584

C 0.350537 2.687378 0.532687

C -0.527888 3.741710 -0.202214

C -1.936374 3.265408 -0.446729

C -1.964810 1.963184 -1.207600

O -2.932179 3.838771 -0.044414

O 0.892560 0.336121 0.552579

H -0.071484 3.955558 -1.178647

H -0.544326 4.667722 0.379209

H -1.470654 2.108502 -2.177200

H -3.001205 1.671576 -1.389531

C 1.794075 3.196759 0.392900

C 0.001202 2.673620 2.039578

C -1.114464 -0.390742 -1.339355

H -0.443537 -1.099104 -0.859946

H -0.679298 -0.110868 -2.305470

C -2.454444 -1.100083 -1.569008

H -2.306566 -2.023663 -2.129445

N -3.069454 -1.458480 -0.295805

H -3.149936 -0.469837 -2.143579

C -1.984676 0.459471 0.854912

H -2.262728 1.346368 1.428752

H -1.322615 -0.144323 1.481615

C -3.284898 -0.324112 0.590917

H -4.041344 0.342376 0.148494

H -3.691087 -0.666584 1.543175

C 2.743345 -0.690210 -1.350343

C 2.431369 -0.408214 0.192406

C 3.371861 0.565496 0.704409

C 2.235448 -1.624720 1.206667

C 2.442070 0.562420 -2.209382

C 4.265310 -0.975878 -1.544695

H 3.901480 1.241131 0.047422

C 2.025729 -1.893131 -2.002625

H 3.482356 0.744053 1.764633

C 3.459470 -2.571238 1.158894

C 0.965781 -2.475812 0.974443

C 2.142785 -1.119971 2.673441

H 3.103665 -0.764427 3.059234

H 1.403169 -0.325061 2.781770

H 1.840023 -1.956653 3.313051

H 0.059765 -1.904029 1.175736

H 0.892042 -2.892125 -0.029002

H 0.984362 -3.323339 1.670434

H 3.508416 -3.158275 0.238216

H 4.401517 -2.024878 1.273427

H 3.386047 -3.283004 1.989922

H 2.318669 -2.846522 -1.556205

H 0.940712 -1.820456 -1.977954

H 2.320700 -1.935810 -3.057666

H 1.408297 0.890119 -2.115070

H 3.081565 1.408378 -1.936576

H 2.652293 0.330134 -3.260022

H 4.899644 -0.160478 -1.188469

H 4.594843 -1.894759 -1.057556

H 4.456254 -1.090080 -2.618713

C -3.409746 -2.768257 -0.046460

O -3.201815 -3.658820 -0.865311

C -4.058244 -3.083323 1.297380

H -3.402484 -2.832292 2.138909

H -5.001770 -2.543076 1.434540

H -4.258589 -4.155056 1.314609

H 1.822991 4.260636 0.655417

H 2.472943 2.675498 1.067042

H 2.153231 3.086544 -0.633699

H 0.435140 3.556844 2.522241

H -1.073118 2.701389 2.233288

H 0.424112 1.785891 2.514917

Zero-point correction= 0.617137 Hartree/Particle

Thermal correction to Energy= 0.647927

Thermal correction to Enthalpy= 0.648871

Thermal correction to Gibbs Free Energy= 0.558548

Sum of electronic and zero-point Energies= -1235.538871

Sum of electronic and thermal Energies= -1235.508081

Sum of electronic and thermal Enthalpies= -1235.507136

Sum of electronic and thermal Free Energies= -1235.597459

The wave number of the imaginary frequency= -353.9807 cm^-1^

Entry **20** (TS1) (UB3LYP)

C -0.591134 -0.244391 1.226351

C -1.308989 -1.558414 0.855543

C -1.620146 0.854600 1.544203

N 0.249847 0.227919 0.099072

C -2.497002 1.101946 0.300581

C -0.547566 0.443082 -1.137558

C -1.576795 1.550358 -0.852615

H 0.089412 -0.396629 2.069840

O 1.281234 -0.639162 -0.130198

H -2.237463 0.540327 2.396126

C -1.267794 -0.858470 -1.549597

C -3.219282 -0.205892 -0.090851

H -1.100389 1.775839 1.835382

H 0.156877 0.757669 -1.912662

H -3.235375 1.885722 0.513973

H -1.058952 2.480986 -0.589239

H -2.162312 1.743509 -1.761269

H -1.922761 -1.902406 1.699277

H -0.559603 -2.332189 0.653554

H -0.521490 -1.629296 -1.773871

H -1.853172 -0.687836 -2.463584

C -2.183759 -1.312264 -0.392754

H -3.883940 -0.524279 0.724470

H -3.854922 -0.034604 -0.970999

H -2.703054 -2.238522 -0.671430

C 2.957336 0.096815 0.100159

C 3.700191 -1.048126 -0.556393

C 3.066677 0.227244 1.497250

C 2.855681 1.369947 -0.711770

H 3.792912 1.937354 -0.654223

H 2.654608 1.151520 -1.765242

H 2.048443 1.999659 -0.323957

H 3.645468 -1.953182 0.056389

H 4.757763 -0.788885 -0.691969

H 3.271167 -1.270351 -1.537860

H 3.354033 -0.617852 2.115604

H 2.719431 1.122711 2.004468

Zero-point correction= 0.332734 Hartree/Particle

Thermal correction to Energy= 0.346478

Thermal correction to Enthalpy= 0.347422

Thermal correction to Gibbs Free Energy= 0.292514

Sum of electronic and zero-point Energies= -638.163226

Sum of electronic and thermal Energies= -638.149482

Sum of electronic and thermal Enthalpies= -638.148537

Sum of electronic and thermal Free Energies= -638.203445

The wave number of the imaginary frequency= -583.0436 cm^-1^

Entry **20** (TS1) (UM06-2X)

C -0.570658 -0.337438 1.197023

C -1.304184 -1.609650 0.747771

C -1.575367 0.748261 1.593538

N 0.257517 0.184119 0.094157

C -2.453598 1.086749 0.380685

C -0.535820 0.504509 -1.110554

C -1.541445 1.599130 -0.742862

H 0.123315 -0.551558 2.016664

O 1.260644 -0.659386 -0.195743

H -2.191524 0.387798 2.426450

C -1.271634 -0.753105 -1.599528

C -3.192820 -0.177460 -0.088901

H -1.036794 1.640301 1.934167

H 0.173624 0.860111 -1.863370

H -3.179363 1.862358 0.651945

H -1.004667 2.498481 -0.418379

H -2.132095 1.860802 -1.629375

H -1.918705 -2.000113 1.569036

H -0.562999 -2.373061 0.486303

H -0.532620 -1.513793 -1.875348

H -1.864337 -0.515767 -2.492172

C -2.178702 -1.269874 -0.470540

H -3.853199 -0.540586 0.709717

H -3.828284 0.061711 -0.951875

H -2.711819 -2.167358 -0.805620

C 2.943285 0.108455 0.118419

C 3.712666 -1.021666 -0.517858

C 2.960802 0.231787 1.503009

C 2.805146 1.362897 -0.707579

H 3.724588 1.956312 -0.661650

H 2.607063 1.116065 -1.754724

H 1.978481 1.970930 -0.325469

H 3.693573 -1.911574 0.116624

H 4.756466 -0.729790 -0.678268

H 3.270975 -1.275842 -1.485133

H 3.253567 -0.601288 2.133225

H 2.560957 1.116538 1.988365

Zero-point correction= 0.335914 (Hartree/Particle)

Thermal correction to Energy= 0.349472

Thermal correction to Enthalpy= 0.350416

Thermal correction to Gibbs Free Energy= 0.295891

Sum of electronic and zero-point Energies= -637.864030

Sum of electronic and thermal Energies= -637.850473

Sum of electronic and thermal Enthalpies= -637.849529

Sum of electronic and thermal Free Energies= -637.904054

The wave number of the imaginary frequency= -856.6447 cm^-1^

Entry **20** (TS2) (UB3LYP)

C -1.705523 0.973134 0.733533

C -2.846264 1.705056 0.002952

C -2.289442 -0.010371 1.769232

N -0.893674 0.255762 -0.286123

C -3.165982 -1.048485 1.036320

C -1.697769 -0.782180 -0.982458

C -2.284961 -1.800511 0.016671

H -1.043749 1.696756 1.215880

O 0.217709 -0.317729 0.304051

H -2.881736 0.540892 2.512422

C -2.835684 -0.073447 -1.738970

C -4.310906 -0.325727 0.292676

H -1.471507 -0.507047 2.303954

H -1.018899 -1.274782 -1.685431

H -3.586148 -1.758422 1.760696

H -1.472100 -2.326917 0.528630

H -2.874125 -2.554313 -0.523495

H -3.437985 2.269660 0.735812

H -2.429638 2.427413 -0.709837

H -2.414269 0.628295 -2.469278

H -3.419806 -0.818113 -2.295782

C -3.727195 0.674318 -0.728562

H -4.952466 0.203828 1.010909

H -4.947217 -1.059250 -0.221983

H -4.541926 1.186988 -1.256356

C 1.860413 0.006240 -0.458138

C 1.608930 -0.058749 -1.852921

C 2.590965 -1.291606 0.081756

C 2.216614 1.451603 0.078981

C 3.712355 1.773018 -0.169859

C 1.940250 1.682836 1.584735

C 1.406228 2.521676 -0.701447

H 1.193322 0.773744 -2.402577

H 1.672567 -0.995246 -2.393600

C 2.852618 -1.300027 1.602462

C 3.952643 -1.476179 -0.639067

C 1.761462 -2.569402 -0.215932

H 1.484689 -2.664778 -1.269782

H 0.846552 -2.595738 0.375285

H 2.365433 -3.448480 0.039538

H 4.430011 -2.393220 -0.272254

H 4.645803 -0.652640 -0.458352

H 3.828078 -1.577952 -1.721972

H 3.621857 -0.582525 1.900976

H 3.215438 -2.293150 1.894022

H 1.941249 -1.097223 2.170254

H 4.379352 1.197101 0.478411

H 3.890831 2.834006 0.045269

H 3.998514 1.591065 -1.211412

H 1.520707 3.492387 -0.206702

H 0.342645 2.275462 -0.746136

H 1.771530 2.644058 -1.726454

H 2.052424 2.751175 1.804769

H 2.640426 1.153574 2.231200

H 0.931685 1.377707 1.862042

Zero-point correction= 0.504145 Hartree/Particle

Thermal correction to Energy= 0.525245

Thermal correction to Enthalpy= 0.526189

Thermal correction to Gibbs Free Energy= 0.456725

Sum of electronic and zero-point Energies= -873.837360

Sum of electronic and thermal Energies= -873.816260

Sum of electronic and thermal Enthalpies= -873.815316

Sum of electronic and thermal Free Energies= -873.884780

The wave number of the imaginary frequency= -578.3306 cm^-1^

Entry **20** (TS2) (UM06-2X)

C -1.688340 0.979641 0.719439

C -2.822087 1.696605 -0.019750

C -2.263338 0.009194 1.762777

N -0.883717 0.232499 -0.271011

C -3.130405 -1.037671 1.042593

C -1.678021 -0.794328 -0.973631

C -2.255363 -1.799059 0.033693

H -1.017578 1.708339 1.184365

O 0.190379 -0.332789 0.320720

H -2.859695 0.562367 2.499492

C -2.808575 -0.094735 -1.734154

C -4.272740 -0.328826 0.294942

H -1.437445 -0.477754 2.294336

H -0.985917 -1.286520 -1.666129

H -3.547617 -1.738880 1.774618

H -1.433747 -2.308960 0.548140

H -2.845645 -2.559999 -0.492628

H -3.415384 2.268775 0.704308

H -2.402694 2.405413 -0.743676

H -2.383092 0.602831 -2.465042

H -3.392063 -0.843698 -2.283635

C -3.697016 0.657979 -0.733992

H -4.911982 0.208373 1.008054

H -4.904145 -1.069995 -0.212692

H -4.513346 1.161010 -1.265165

C 1.871329 0.006959 -0.480184

C 1.572372 -0.076391 -1.843111

C 2.591685 -1.264320 0.077037

C 2.182035 1.438122 0.070191

C 3.668258 1.772441 -0.163364

C 1.889628 1.654575 1.570168

C 1.366512 2.488673 -0.712506

H 1.135204 0.745019 -2.392796

H 1.641333 -1.018214 -2.374529

C 2.895803 -1.213240 1.580116

C 3.920387 -1.479817 -0.676579

C 1.742691 -2.534599 -0.142726

H 1.362299 -2.620824 -1.164099

H 0.889915 -2.559121 0.536183

H 2.368270 -3.413142 0.052375

H 4.436329 -2.349866 -0.254013

H 4.594092 -0.624124 -0.599338

H 3.746640 -1.676582 -1.738848

H 3.680755 -0.491790 1.823972

H 3.254650 -2.198584 1.898466

H 2.000288 -0.975559 2.160270

H 4.329988 1.178998 0.474294

H 3.841725 2.827898 0.077768

H 3.951823 1.609682 -1.208785

H 1.479682 3.464226 -0.229843

H 0.303545 2.232945 -0.748287

H 1.729001 2.596766 -1.739249

H 1.867641 2.731007 1.772027

H 2.660604 1.231706 2.214641

H 0.930667 1.225862 1.861015

Zero-point correction= 0.509388 (Hartree/Particle)

Thermal correction to Energy= 0.529835

Thermal correction to Enthalpy= 0.530779

Thermal correction to Gibbs Free Energy= 0.463189

Sum of electronic and zero-point Energies= -873.433652

Sum of electronic and thermal Energies= -873.413205

Sum of electronic and thermal Enthalpies= -873.412261

Sum of electronic and thermal Free Energies= -873.479851

The wave number of the imaginary frequency= -907.4723 cm^-1^

Entry **21** (TS1) (UB3LYP)

C -0.678597 1.164997 -0.002080

C -1.377955 0.969963 1.365998

C -1.748279 1.164873 -1.118419

N 0.228271 -0.002802 -0.273870

C -2.546649 -0.149591 -1.112976

C -0.503436 -1.302631 -0.243424

C -1.556579 -1.301103 -1.362037

C 0.122242 2.464350 -0.038407

O 1.268305 -0.043398 0.620031

H -2.417471 2.021597 -0.962171

C -1.180115 -1.513750 1.125540

C -3.231113 -0.335444 0.257681

H -1.256572 1.308023 -2.089292

H 0.244367 -2.078543 -0.424293

H -3.303363 -0.127069 -1.908126

H -1.066201 -1.179926 -2.336044

H -2.076512 -2.268318 -1.371905

H -2.050755 1.817294 1.558044

H -0.618509 0.968614 2.157122

H -0.412805 -1.542379 1.907401

H -1.703254 -2.479810 1.137698

C -2.165853 -0.355737 1.375262

H -3.946444 0.480298 0.432996

H -3.806353 -1.271817 0.267166

H -2.655176 -0.486112 2.349496

C 2.947343 -0.149673 -0.096986

C 3.586717 -0.730912 1.148065

C 3.269651 1.185067 -0.419892

C 2.773394 -1.106387 -1.257681

H 3.709547 -1.192701 -1.822500

H 2.494124 -2.106693 -0.912887

H 1.995610 -0.738738 -1.935089

H 3.564513 -0.009045 1.970444

H 4.633614 -0.994053 0.951223

H 3.056978 -1.632777 1.467703

H 3.623582 1.874192 0.340930

H 3.027826 1.599172 -1.393992

H -0.548876 3.316084 0.122881

H 0.891778 2.474006 0.736371

H 0.611393 2.591368 -1.010196

Zero-point correction= 0.360516 Hartree/Particle

Thermal correction to Energy= 0.375645

Thermal correction to Enthalpy= 0.376589

Thermal correction to Gibbs Free Energy= 0.319254

Sum of electronic and zero-point Energies= -677.450234

Sum of electronic and thermal Energies= -677.435105

Sum of electronic and thermal Enthalpies= -677.434161

Sum of electronic and thermal Free Energies= -677.491496

The wave number of the imaginary frequency= -578.8620 cm^-1^

Entry **21** (TS1) (UM06-2X)

C -0.656901 1.151796 -0.016238

C -1.360919 0.992886 1.344394

C -1.709773 1.153294 -1.135262

N 0.229741 -0.026214 -0.248153

C -2.522648 -0.145128 -1.115194

C -0.512213 -1.309956 -0.213759

C -1.550255 -1.309510 -1.337177

C 0.169771 2.426561 -0.064243

O 1.241251 -0.058255 0.639136

H -2.367072 2.021454 -0.994909

C -1.197486 -1.487228 1.148274

C -3.215479 -0.298756 0.248260

H -1.203891 1.271935 -2.101707

H 0.229767 -2.095507 -0.375058

H -3.272654 -0.125978 -1.914743

H -1.046787 -1.206137 -2.305766

H -2.084679 -2.267591 -1.335565

H -2.021504 1.853327 1.516429

H -0.599540 0.991148 2.134086

H -0.432342 -1.508772 1.932398

H -1.735254 -2.443688 1.172928

C -2.164182 -0.315879 1.370574

H -3.917960 0.530563 0.404575

H -3.800030 -1.227954 0.267566

H -2.662200 -0.423468 2.341371

C 2.945918 -0.151943 -0.123970

C 3.633185 -0.445315 1.186095

C 3.118709 1.103699 -0.696278

C 2.761160 -1.326729 -1.052404

H 3.685612 -1.525777 -1.605001

H 2.497953 -2.227821 -0.491391

H 1.965745 -1.111088 -1.773886

H 3.657246 0.445065 1.820064

H 4.662558 -0.776728 1.010357

H 3.098996 -1.236219 1.719347

H 3.459098 1.947205 -0.104961

H 2.802175 1.299291 -1.715580

H -0.483569 3.291234 0.094224

H 0.940408 2.417289 0.709817

H 0.656419 2.530999 -1.039053

Zero-point correction= 0.363970 (Hartree/Particle)

Thermal correction to Energy= 0.378800

Thermal correction to Enthalpy= 0.379744

Thermal correction to Gibbs Free Energy= 0.323092

Sum of electronic and zero-point Energies= -677.134590

Sum of electronic and thermal Energies= -677.119761

Sum of electronic and thermal Enthalpies= -677.118817

Sum of electronic and thermal Free Energies= -677.175469

The wave number of the imaginary frequency= -866.2028 cm^-1^

Entry **21** (TS2) (UB3LYP)

C -1.815491 0.043116 1.162280

C -2.418812 -1.372360 0.986092

C -2.945572 1.091461 1.025671

N -0.826012 0.343555 0.061390

C -3.640736 0.977017 -0.340176

C -1.466136 0.176678 -1.281959

C -2.579875 1.226443 -1.425828

C -1.161255 0.179144 2.534192

O 0.261807 -0.529736 0.114450

H -3.668046 0.932941 1.837551

C -2.051869 -1.237003 -1.471975

C -4.232691 -0.439371 -0.500538

H -2.524034 2.096307 1.157003

H -0.683604 0.348172 -2.021187

H -4.440018 1.726065 -0.415249

H -2.159930 2.235207 -1.326209

H -3.020801 1.149789 -2.428690

H -3.136032 -1.562851 1.796489

H -1.618809 -2.116636 1.077749

H -1.249798 -1.981054 -1.411788

H -2.495103 -1.313715 -2.474477

C -3.109959 -1.492573 -0.385193

H -4.997078 -0.614960 0.269521

H -4.734817 -0.529405 -1.473988

H -3.533434 -2.499115 -0.500124

C 1.952048 0.027216 0.421905

C 2.655790 -1.269593 -0.174567

C 2.002761 0.037556 1.848442

C 2.171180 1.458655 -0.216828

C 3.687911 1.773965 -0.294706

C 1.575890 1.679188 -1.627836

C 1.541737 2.569923 0.664829

C 1.968970 -2.569559 0.331417

C 4.130687 -1.356211 0.303882

C 2.650747 -1.355574 -1.713748

H 2.141250 -0.878460 2.409230

H 1.756999 0.912370 2.431308

H -1.913848 0.011660 3.313670

H -0.360904 -0.551417 2.666503

H -0.744543 1.181880 2.676516

H 0.495083 2.349498 0.886792

H 2.087644 2.731344 1.599741

H 1.576983 3.515860 0.111550

H 1.762186 0.864677 -2.324795

H 0.501965 1.850585 -1.570312

H 2.022843 2.585275 -2.055730

H 4.192138 1.591600 0.659724

H 4.198374 1.199901 -1.073173

H 3.813868 2.835286 -0.541167

H 1.908348 -2.625204 1.421890

H 0.960449 -2.677572 -0.066143

H 2.565165 -3.427515 -0.002086

H 1.634131 -1.296829 -2.111088

H 3.262019 -0.580443 -2.185104

H 3.072333 -2.321529 -2.017266

H 4.756765 -0.550982 -0.082699

H 4.205345 -1.345097 1.395947

H 4.562582 -2.301347 -0.047374

Zero-point correction= 0.532377 Hartree/Particle

Thermal correction to Energy= 0.554606

Thermal correction to Enthalpy= 0.555551

Thermal correction to Gibbs Free Energy= 0.484386

Sum of electronic and zero-point Energies= -913.117614

Sum of electronic and thermal Energies= -913.095384

Sum of electronic and thermal Enthalpies= -913.094440

Sum of electronic and thermal Free Energies= -913.165604

The wave number of the imaginary frequency= -503.6610 cm^-1^

Entry **21** (TS2) (UM06-2X)

C -1.787454 0.043844 1.153361

C -2.404516 -1.359156 1.000879

C -2.891717 1.105231 1.024876

N -0.813922 0.300720 0.046109

C -3.598438 0.989562 -0.329148

C -1.459410 0.147596 -1.286492

C -2.550224 1.211102 -1.425891

C -1.098476 0.172408 2.501015

O 0.234196 -0.561177 0.109260

H -3.608588 0.965229 1.844402

C -2.060340 -1.255593 -1.451407

C -4.209170 -0.414635 -0.469851

H -2.444206 2.100808 1.140340

H -0.675078 0.295725 -2.029999

H -4.386696 1.747762 -0.404836

H -2.107905 2.210765 -1.334403

H -3.006004 1.133240 -2.420727

H -3.113402 -1.532386 1.821589

H -1.603687 -2.104374 1.087541

H -1.261273 -2.002880 -1.379760

H -2.512243 -1.342594 -2.447817

C -3.107938 -1.482090 -0.356306

H -4.965487 -0.570856 0.310659

H -4.718606 -0.505133 -1.438317

H -3.549184 -2.480551 -0.457998

C 1.984145 0.034055 0.440650

C 2.652563 -1.245917 -0.175228

C 2.012676 0.040885 1.843363

C 2.138534 1.445392 -0.213055

C 3.638049 1.767539 -0.378052

C 1.456201 1.657059 -1.578555

C 1.551572 2.547530 0.692510

C 1.976754 -2.539648 0.336020

C 4.131924 -1.334498 0.258189

C 2.591489 -1.316050 -1.705314

H 2.178037 -0.870815 2.404061

H 1.768516 0.913391 2.430000

H -1.838374 0.038940 3.297433

H -0.321409 -0.586861 2.613998

H -0.644651 1.162025 2.617020

H 0.518284 2.315012 0.967580

H 2.145772 2.714027 1.595700

H 1.547753 3.489192 0.132761

H 1.569102 0.828001 -2.274042

H 0.394291 1.860490 -1.440366

H 1.897924 2.544476 -2.047010

H 4.188533 1.593392 0.552277

H 4.104655 1.184591 -1.177195

H 3.743690 2.826170 -0.641563

H 1.898586 -2.580922 1.425335

H 0.975994 -2.660945 -0.078237

H 2.586955 -3.393418 0.020973

H 1.554553 -1.270726 -2.051078

H 3.168041 -0.523393 -2.190686

H 3.015853 -2.272096 -2.032326

H 4.740649 -0.514880 -0.125477

H 4.226661 -1.345499 1.348576

H 4.559487 -2.268211 -0.124620

Zero-point correction= 0.538382 (Hartree/Particle)

Thermal correction to Energy= 0.559895

Thermal correction to Enthalpy= 0.560840

Thermal correction to Gibbs Free Energy= 0.491436

Sum of electronic and zero-point Energies= -912.697812

Sum of electronic and thermal Energies= -912.676298

Sum of electronic and thermal Enthalpies= -912.675354

Sum of electronic and thermal Free Energies= -912.744758

The wave number of the imaginary frequency= -834.9256 cm^-1^

Entry **22** (TS1) (UB3LYP)

C -0.647106 -1.248451 0.133960

C -1.293182 -1.255319 -1.272225

C -1.764010 -1.234803 1.200359

N 0.163197 0.006482 0.322097

C -2.660836 -0.001487 1.026643

C -0.655795 1.253824 0.124177

C -1.772747 1.239433 1.190820

C 0.236663 -2.478197 0.338400

O 1.240872 0.004392 -0.545720

H -2.351910 -2.157422 1.103006

C -1.302390 1.245286 -1.281892

C -3.293794 -0.008952 -0.379691

H -1.310431 -1.239698 2.199898

C 0.217123 2.492524 0.319027

H -3.448883 -0.001222 1.791400

H -1.319318 1.255573 2.190309

H -2.367431 2.156893 1.086090

H -1.885956 -2.171603 -1.401866

H -0.499617 -1.269605 -2.029109

H -0.509116 1.259695 -2.039130

H -1.901906 2.156189 -1.418334

C -2.181288 -0.008924 -1.448659

H -3.933024 -0.894794 -0.499723

H -3.939475 0.871293 -0.506265

H -2.630814 -0.014387 -2.450493

C 2.927968 -0.007410 0.086223

C 3.448378 -1.196445 -0.701809

C 2.884872 -0.139995 1.496610

C 3.421983 1.320065 -0.460907

H 4.519578 1.335565 -0.470288

H 3.072918 1.461489 -1.488884

H 3.078525 2.162987 0.143425

H 3.136545 -2.145603 -0.259018

H 4.545438 -1.179079 -0.730168

H 3.081549 -1.156122 -1.732599

H 2.806733 -1.115744 1.966007

H 2.757015 0.727606 2.136614

H -0.383194 -3.382269 0.328632

H 0.982009 -2.568797 -0.453422

H 0.752390 -2.428263 1.303610

H 0.744283 2.447867 1.278756

H 0.952386 2.592281 -0.480836

H -0.412678 3.389757 0.317252

Zero-point correction= 0.388293 Hartree/Particle

Thermal correction to Energy= 0.404733

Thermal correction to Enthalpy= 0.405677

Thermal correction to Gibbs Free Energy= 0.346030

Sum of electronic and zero-point Energies= -716.731151

Sum of electronic and thermal Energies= -716.714711

Sum of electronic and thermal Enthalpies= -716.713767

Sum of electronic and thermal Free Energies= -716.773413

The wave number of the imaginary frequency= -653.5509 cm^-1^

Entry **22** (TS1) (UM06-2X)

C 0.647331 1.244073 0.116569

C 1.313202 1.245297 -1.271199

C 1.734350 1.234813 1.200700

N -0.157816 -0.000008 0.282724

C 2.625396 0.000016 1.051003

C 0.647354 -1.244076 0.116574

C 1.734373 -1.234795 1.200704

C -0.256836 2.453873 0.291306

O -1.210451 -0.000025 -0.565533

H 2.328285 2.153538 1.108420

C 1.313223 -1.245293 -1.271195

C 3.282265 0.000019 -0.337597

H 1.255080 1.244316 2.187894

C -0.256789 -2.453892 0.291325

H 3.397871 0.000027 1.829366

H 1.255101 -1.244301 2.187897

H 2.328322 -2.153511 1.108431

H 1.908827 2.160235 -1.392692

H 0.526892 1.253971 -2.036407

H 0.526912 -1.253983 -2.036402

H 1.908866 -2.160218 -1.392686

C 2.196722 0.000010 -1.425637

H 3.923917 0.884324 -0.447010

H 3.923929 -0.884276 -0.447006

H 2.666480 0.000013 -2.416486

C -2.935777 -0.000014 0.108134

C -3.412065 1.264410 -0.567436

C -2.827459 -0.000122 1.500964

C -3.412113 -1.264304 -0.567650

H -4.505986 -1.264117 -0.636997

H -3.004037 -1.322187 -1.581627

H -3.103932 -2.154622 -0.014333

H -3.103785 2.154621 -0.014005

H -4.505942 1.264311 -0.636714

H -3.004052 1.322416 -1.581432

H -2.714933 0.926532 2.053485

H -2.714900 -0.926856 2.053343

H 0.348587 3.365880 0.317946

H -0.966582 2.532165 -0.534224

H -0.813919 2.377821 1.231624

H -0.813793 -2.377889 1.231694

H -0.966601 -2.532152 -0.534151

H 0.348646 -3.365895 0.317874

Zero-point correction= 0.391579 (Hartree/Particle)

Thermal correction to Energy= 0.407767

Thermal correction to Enthalpy= 0.408711

Thermal correction to Gibbs Free Energy= 0.349443

Sum of electronic and zero-point Energies= -716.400180

Sum of electronic and thermal Energies= -716.383993

Sum of electronic and thermal Enthalpies= -716.383049

Sum of electronic and thermal Free Energies= -716.442316

The wave number of the imaginary frequency= -970.3868 cm^-1^

Entry **22** (TS2) (UB3LYP)

C 1.724947 0.600482 -1.096076

C 2.286103 -0.742194 -1.619728

C 2.901440 1.498083 -0.647560

N 0.830344 0.388094 0.120990

C 3.733080 0.808765 0.438088

C 1.614566 -0.349321 1.210512

C 2.795005 0.557880 1.622556

C 0.993706 1.314828 -2.230170

O -0.271093 -0.429688 -0.233000

H 3.520802 1.718659 -1.527095

C 2.185226 -1.686277 0.670031

C 4.282343 -0.529573 -0.096102

H 2.507996 2.454439 -0.279188

C 0.776408 -0.640852 2.455141

H 4.563484 1.457907 0.746543

H 2.402152 1.507887 2.006854

H 3.330464 0.069236 2.447911

H 2.903192 -0.551294 -2.508761

H 1.450951 -1.379168 -1.933497

H 1.359287 -2.348822 0.386436

H 2.736946 -2.184980 1.478621

C 3.111415 -1.435915 -0.527624

H 4.953503 -0.347127 -0.947071

H 4.878726 -1.028835 0.680383

H 3.501876 -2.391348 -0.902981

C -1.974802 0.124021 -0.452361

C -2.665952 -1.301580 -0.246883

C -1.987587 0.476926 -1.842027

C -2.328416 1.352212 0.481334

C -3.855796 1.618906 0.445507

C -1.900792 1.223505 1.956979

C -1.641168 2.655122 -0.005751

C -1.843866 -2.435383 -0.918486

C -4.060049 -1.319634 -0.938938

C -2.892626 -1.737035 1.214729

H -1.996376 -0.285978 -2.611062

H -1.887237 1.494515 -2.187057

H 1.711740 1.530986 -3.029985

H 0.203045 0.692631 -2.650034

H 0.561153 2.264704 -1.903472

H -0.560977 2.519195 -0.078803

H -2.028809 3.023937 -0.960451

H -1.831361 3.442807 0.732625

H -2.210995 0.297979 2.437158

H -0.820506 1.314819 2.039957

H -2.347977 2.051153 2.521480

H -4.234473 1.691304 -0.579482

H -4.430888 0.853437 0.974172

H -4.057704 2.575112 0.943497

H -1.577622 -2.213365 -1.955642

H -0.922039 -2.644308 -0.376553

H -2.453825 -3.347096 -0.925438

H -1.962259 -1.772823 1.783922

H -3.603269 -1.095859 1.743508

H -3.314469 -2.749535 1.216239

H -4.756550 -0.590683 -0.521672

H -3.988863 -1.136531 -2.015036

H -4.506918 -2.312362 -0.804198

H 0.531552 0.267975 3.009668

H -0.145966 -1.161369 2.202371

H 1.359439 -1.284147 3.124169

Zero-point correction= 0.559970 Hartree/Particle

Thermal correction to Energy= 0.583640

Thermal correction to Enthalpy= 0.584585

Thermal correction to Gibbs Free Energy= 0.511318

Sum of electronic and zero-point Energies= -952.390247

Sum of electronic and thermal Energies= -952.366576

Sum of electronic and thermal Enthalpies= -952.365632

Sum of electronic and thermal Free Energies= -952.438898

The wave number of the imaginary frequency= -483.2369 cm^-1^

Entry **22** (TS2) (UM06-2X)

C 1.704019 0.532699 -1.117775

C 2.287455 -0.813332 -1.582358

C 2.847699 1.476737 -0.718649

N 0.823826 0.349645 0.095450

C 3.692321 0.860390 0.395109

C 1.615682 -0.308334 1.213357

C 2.765124 0.636575 1.587816

C 0.943940 1.153957 -2.278424

O -0.242190 -0.463128 -0.216547

H 3.462889 1.670010 -1.606851

C 2.207268 -1.649838 0.729925

C 4.269952 -0.482475 -0.078752

H 2.422226 2.435000 -0.393270

C 0.779319 -0.583544 2.455616

H 4.506414 1.540507 0.672671

H 2.343972 1.590007 1.931390

H 3.312423 0.191935 2.429660

H 2.891454 -0.647287 -2.484606

H 1.456555 -1.477236 -1.852775

H 1.385296 -2.328884 0.469789

H 2.762965 -2.105089 1.560162

C 3.128964 -1.434221 -0.469220

H 4.934005 -0.319836 -0.938004

H 4.874778 -0.932129 0.719819

H 3.543589 -2.393819 -0.800593

C -2.017117 0.126873 -0.470736

C -2.678294 -1.277213 -0.212900

C -2.001363 0.442384 -1.844203

C -2.303885 1.353968 0.450809

C -3.823366 1.605140 0.521248

C -1.749582 1.251608 1.875738

C -1.669148 2.643081 -0.110106

C -1.862145 -2.417438 -0.861625

C -4.077909 -1.315450 -0.872126

C -2.866019 -1.667824 1.258196

H -2.058615 -0.335273 -2.596577

H -1.845773 1.443165 -2.215229

H 1.657361 1.381765 -3.077771

H 0.203657 0.458541 -2.675738

H 0.447813 2.085201 -1.992863

H -0.596660 2.503881 -0.268209

H -2.139857 2.991122 -1.033920

H -1.797530 3.439473 0.630733

H -1.980862 0.319764 2.387035

H -0.668935 1.378457 1.845288

H -2.169508 2.071233 2.470757

H -4.262344 1.670996 -0.480101

H -4.353230 0.832270 1.084148

H -3.998862 2.559495 1.030873

H -1.571076 -2.199340 -1.892680

H -0.953611 -2.629679 -0.297698

H -2.481734 -3.321241 -0.874709

H -1.913931 -1.715013 1.788801

H -3.538241 -0.995310 1.797451

H -3.311273 -2.668658 1.295233

H -4.757788 -0.561014 -0.472865

H -4.019123 -1.176967 -1.955346

H -4.529186 -2.297011 -0.687944

H 0.537362 0.326550 3.007048

H -0.141817 -1.101977 2.192866

H 1.360458 -1.228559 3.122599

Zero-point correction= 0.565039 (Hartree/Particle)

Thermal correction to Energy= 0.588275

Thermal correction to Enthalpy= 0.589219

Thermal correction to Gibbs Free Energy= 0.516709

Sum of electronic and zero-point Energies= -951.954869

Sum of electronic and thermal Energies= -951.931634

Sum of electronic and thermal Enthalpies= -951.930689

Sum of electronic and thermal Free Energies= -952.003199

The wave number of the imaginary frequency= -797.6991cm^-1^

Entry **23** (TS1)

C -0.798085 1.257316 -0.211245

C -1.611798 1.335553 1.095350

C -1.938918 1.115118 -1.229068

N 0.033571 0.017952 -0.243109

C -2.696752 -0.090120 -0.627563

C -0.732162 -1.242434 0.008700

C -1.872473 -1.340751 -1.012620

H -0.120664 2.096558 -0.385222

O 1.069077 0.124040 0.642700

H -2.541709 2.030803 -1.230129

C -1.547304 -1.130748 1.312388

H -1.595861 0.932426 -2.252405

H -0.016856 -2.066679 -0.015855

H -3.747902 -0.144930 -0.923246

H -1.534557 -1.321566 -2.053550

H -2.425958 -2.273104 -0.850828

H -2.215643 2.251546 1.112134

H -0.981937 1.324392 1.989521

H -0.925106 -0.932084 2.189808

H -2.103798 -2.059725 1.489728

C -2.472832 0.056318 0.949905

H -3.402661 0.081971 1.524362

C 2.743119 0.062791 -0.131009

C 3.484556 -0.183360 1.166607

C 2.869221 1.333871 -0.722317

C 2.625316 -1.127337 -1.058475

H 3.556278 -1.267088 -1.621822

H 2.426607 -2.046795 -0.498864

H 1.811337 -0.969492 -1.773357

H 3.442752 0.696954 1.815419

H 4.538869 -0.411913 0.965763

H 3.044842 -1.028420 1.704342

H 3.165493 2.196028 -0.132421

H 2.527522 1.515832 -1.737126

Zero-point correction= 0.302764 Hartree/Particle

Thermal correction to Energy= 0.315651

Thermal correction to Enthalpy= 0.316595

Thermal correction to Gibbs Free Energy= 0.263309

Sum of electronic and zero-point Energies= -598.856210

Sum of electronic and thermal Energies= -598.843323

Sum of electronic and thermal Enthalpies= -598.842379

Sum of electronic and thermal Free Energies= -598.895665

The wave number of the imaginary frequency= -578.6563 cm^-1^

Entry **23** (TS2)

C 1.872033 -0.850063 -0.947554

C 2.564579 -1.784408 0.063950

C 3.105093 -0.225504 -1.616400

N 1.100623 0.228692 -0.259833

C 3.865358 0.284652 -0.371553

C 1.902269 0.985904 0.750633

C 3.138668 1.578360 0.057883

H 1.181358 -1.346434 -1.633105

O -0.018040 -0.311477 0.347180

H 3.657737 -1.002238 -2.157756

C 2.589980 0.029290 1.745666

H 2.863433 0.575062 -2.322633

H 1.244486 1.727671 1.205548

H 4.940243 0.410133 -0.527409

H 2.900567 2.232881 -0.786455

H 3.714470 2.159847 0.787377

H 3.122170 -2.566203 -0.466894

H 1.868387 -2.272245 0.752103

H 1.884643 -0.608315 2.286505

H 3.166925 0.602690 2.482102

C 3.493154 -0.768672 0.772905

H 4.363939 -1.224295 1.251820

C -1.649217 -0.023644 -0.454617

C -2.399617 -1.271028 0.170111

C -1.382445 -0.194552 -1.837986

C -1.997064 1.459989 -0.030697

C -3.485361 1.778038 -0.324298

C -1.741180 1.797389 1.459551

C -1.164254 2.463122 -0.874421

C -1.575869 -2.573460 -0.014386

C -3.750158 -1.499359 -0.558642

C -2.687637 -1.158389 1.681702

H -1.443309 -1.169676 -2.305750

H -0.959361 0.593004 -2.445151

H -0.101962 2.208037 -0.880192

H -1.510206 2.509634 -1.912316

H -1.281147 3.469166 -0.456841

H -2.473459 1.345260 2.129070

H -0.750630 1.478757 1.782699

H -1.817931 2.882692 1.594546

H -3.757539 1.532513 -1.356567

H -4.168280 1.251412 0.348690

H -3.655798 2.852385 -0.180391

H -1.297402 -2.758914 -1.055846

H -0.661895 -2.554171 0.578720

H -2.185874 -3.424473 0.312050

H -1.784486 -0.918404 2.248077

H -3.455296 -0.413295 1.909099

H -3.064769 -2.122525 2.043675

H -4.439288 -0.659568 -0.452247

H -3.607946 -1.682236 -1.628593

H -4.241811 -2.383168 -0.133626

Zero-point correction= 0.473994 Hartree/Particle

Thermal correction to Energy= 0.494242

Thermal correction to Enthalpy= 0.495186

Thermal correction to Gibbs Free Energy= 0.427335

Sum of electronic and zero-point Energies= -834.530593

Sum of electronic and thermal Energies= -834.510345

Sum of electronic and thermal Enthalpies= -834.509401

Sum of electronic and thermal Free Energies= -834.577252

The wave number of the imaginary frequency= -573.8835 cm^-1^

Entry **24** (TS1)

C 0.837744 1.278145 0.105756

C 1.561041 1.231673 -1.259437

C 2.062761 1.222101 1.037568

N 0.037344 0.006362 0.310987

C 2.807689 -0.006326 0.488925

C 0.826467 -1.274446 0.114605

C 2.052336 -1.224915 1.045172

C -0.032230 2.506689 0.326234

O -1.048003 0.005507 -0.547636

H 2.635523 2.149455 0.916336

C 1.548855 -1.243394 -1.251328

H 1.797901 1.124637 2.095698

C -0.056624 -2.492078 0.344493

H 3.879749 -0.010072 0.705769

H 1.789153 -1.118869 2.102892

H 2.616554 -2.158141 0.928984

H 2.142552 2.151203 -1.405408

H 0.867703 1.132485 -2.100646

H 0.855896 -1.142556 -2.092593

H 2.121405 -2.169324 -1.392102

C 2.460199 -0.009660 -1.070902

H 3.342089 -0.016084 -1.717585

C -2.728972 -0.007051 0.096732

C -3.227339 1.327329 -0.429926

C -2.676005 -0.156645 1.505578

C -3.260383 -1.184651 -0.701519

H -4.357345 -1.159053 -0.726167

H -2.896277 -1.136405 -1.732937

H -2.954695 -2.140750 -0.269521

H -2.883661 2.162681 0.184580

H -4.324985 1.340663 -0.434359

H -2.883009 1.483384 -1.457378

H -2.540093 0.702774 2.154949

H -2.598528 -1.138186 1.962791

H 0.596465 3.404727 0.314346

H -0.785552 2.609432 -0.456082

H -0.539410 2.458526 1.296395

H -0.544938 -2.442235 1.323714

H -0.825660 -2.578454 -0.424627

H 0.558531 -3.399058 0.315414

Zero-point correction= 0.358150 Hartree/Particle

Thermal correction to Energy= 0.373843

Thermal correction to Enthalpy= 0.374787

Thermal correction to Gibbs Free Energy= 0.316303

Sum of electronic and zero-point Energies= -677.425559

Sum of electronic and thermal Energies= -677.409866

Sum of electronic and thermal Enthalpies= -677.408922

Sum of electronic and thermal Free Energies= -677.467406

The wave number of the imaginary frequency= -647.9617 cm^-1^

Entry **24** (TS2)

C 1.913601 0.851482 -0.924533

C 2.555682 -0.299596 -1.728488

C 3.185132 1.447870 -0.288832

N 1.024628 0.311418 0.206782

C 3.839023 0.203186 0.328908

C 1.768967 -0.721806 1.077939

C 3.049680 -0.059399 1.619620

C 1.200127 1.861314 -1.809841

O -0.083959 -0.362607 -0.360414

H 3.794646 1.889380 -1.086354

C 2.416927 -1.815762 0.195862

H 2.976172 2.232554 0.446084

C 0.918572 -1.302207 2.198474

H 4.919578 0.290742 0.474443

H 2.853976 0.850902 2.196058

H 3.551041 -0.774721 2.283066

H 3.166510 0.120220 -2.538030

H 1.813813 -0.966926 -2.177173

H 1.693512 -2.363567 -0.415320

H 2.927323 -2.540608 0.842889

C 3.404863 -0.986163 -0.643445

H 4.245284 -1.565036 -1.037181

C -1.783658 0.243091 -0.407976

C -2.496723 -1.178561 -0.562726

C -1.792411 0.932299 -1.665515

C -2.118145 1.204333 0.804740

C -3.640208 1.501750 0.834558

C -1.702197 0.700701 2.201358

C -1.403869 2.574174 0.663137

C -1.680788 -2.129258 -1.480213

C -3.881662 -1.002130 -1.250223

C -2.753836 -1.953530 0.745985

H -1.798407 0.384257 -2.599873

H -1.687325 2.003360 -1.745505

H 1.927327 2.275383 -2.518622

H 0.399950 1.396646 -2.386484

H 0.782649 2.693208 -1.236218

H -0.325361 2.440428 0.568468

H -1.772520 3.176284 -0.172845

H -1.591556 3.156337 1.572835

H -2.040463 -0.306078 2.436280

H -0.620247 0.739186 2.303007

H -2.130400 1.373906 2.954421

H -4.010448 1.838113 -0.139593

H -4.233838 0.639621 1.151562

H -3.827536 2.305001 1.557627

H -1.383439 -1.662018 -2.423071

H -0.776988 -2.487722 -0.988261

H -2.307162 -2.995794 -1.725977

H -1.834788 -2.150190 1.300556

H -3.460765 -1.446049 1.408210

H -3.194392 -2.926084 0.494514

H -4.562414 -0.364304 -0.684419

H -3.793878 -0.586330 -2.258260

H -4.356063 -1.986679 -1.342130

H 0.705864 -0.567730 2.978781

H -0.022027 -1.703064 1.825219

H 1.477186 -2.122441 2.664401

Zero-point correction= 0.530530 Hartree/Particle

Thermal correction to Energy= 0.553205

Thermal correction to Enthalpy= 0.554149

Thermal correction to Gibbs Free Energy= 0.482788

Sum of electronic and zero-point Energies= -913.084140

Sum of electronic and thermal Energies= -913.061465

Sum of electronic and thermal Enthalpies= -913.060521

Sum of electronic and thermal Free Energies= -913.131882

The wave number of the imaginary frequency= -475.5475 cm^-1^

Entry **25** (TS1)

C 2.812114 -0.578651 1.428896

C 2.717415 -0.165346 0.086553

C 3.503013 1.062376 -0.324948

C 2.581874 -1.244828 -0.965692

H 2.429705 -1.544916 1.744569

H 3.122560 0.114215 2.205219

H 3.095234 1.487580 -1.246791

H 4.554149 0.801032 -0.500590

H 3.466690 1.830066 0.454071

H 2.400723 -0.812179 -1.954606

H 1.749129 -1.910013 -0.715870

H 3.498169 -1.845808 -1.019361

O 1.068795 0.655478 0.011334

N 0.010494 -0.209001 0.050328

C -0.840401 0.038984 1.234452

C -1.467596 1.450402 1.169018

C -2.209213 1.737191 -0.151948

C -1.821540 -1.144769 1.353137

C -0.781013 -0.150874 -1.201114

C -1.411415 1.250737 -1.378481

C -1.759374 -1.342566 -1.186576

C -2.639268 -1.410582 0.075622

H -0.070411 -0.307439 -2.018942

H -1.155505 -2.257331 -1.247820

H -0.165389 0.007221 2.096670

H -1.222161 -2.036013 1.580318

H -0.581637 1.945245 -1.557484

H -2.385424 -1.313700 -2.087756

H -0.640911 2.163157 1.273237

H -2.041571 1.266845 -2.277523

H -2.489871 -0.983915 2.208739

H -3.202274 1.276171 -0.137621

H -2.135267 1.605994 2.026630

H -2.385565 2.817067 -0.240974

H -3.107780 -2.400835 0.141173

H -3.463986 -0.695705 -0.001765

Zero-point correction= 0.325329 Hartree/Particle

Thermal correction to Energy= 0.339593

Thermal correction to Enthalpy= 0.340537

Thermal correction to Gibbs Free Energy= 0.284616

Sum of electronic and zero-point Energies= -600.054123

Sum of electronic and thermal Energies= -600.039859

Sum of electronic and thermal Enthalpies= -600.038915

Sum of electronic and thermal Free Energies= -600.094836

The wave number of the imaginary frequency= -583.1690 cm^-1^

Entry **25** (TS2)

C 1.362207 0.053327 -1.857855

C 1.645163 0.050483 -0.466659

C 2.434584 -1.247126 -0.017671

C 1.647540 -2.536351 -0.374373

C 3.788141 -1.336672 -0.771798

C 2.726094 -1.332035 1.495125

C 1.968697 1.477086 0.140049

C 3.447045 1.861622 -0.121476

C 1.108208 2.560005 -0.565458

C 1.717709 1.619146 1.661161

H 0.902171 0.898513 -2.349442

H 1.442451 -0.850017 -2.450026

H 2.284108 -3.405559 -0.169584

H 1.364372 -2.587630 -1.429666

H 0.740430 -2.623516 0.223073

H 4.305232 -2.257045 -0.473283

H 4.454392 -0.501366 -0.549525

H 3.647502 -1.373102 -1.857056

H 3.130354 -2.325538 1.723875

H 1.818810 -1.196008 2.088769

H 3.473906 -0.603696 1.820451

H 3.593182 2.917321 0.139412

H 3.717305 1.737842 -1.175676

H 4.146557 1.280680 0.486779

H 1.197454 3.504825 -0.018129

H 0.053485 2.278593 -0.607779

H 1.450608 2.752401 -1.587736

H 1.808868 2.676498 1.937075

H 2.442644 1.072941 2.264761

H 0.722334 1.274585 1.939596

O 0.037660 -0.369615 0.312683

N -1.104048 0.191271 -0.230061

C -1.907258 -0.832335 -0.938070

C -2.420223 -1.923167 0.031338

C -3.143635 -1.367336 1.274350

C -2.989443 -0.085833 -1.744395

C -1.915903 0.859658 0.815208

C -2.407918 -0.153994 1.873913

C -3.014958 1.670510 0.095051

C -3.865133 0.844917 -0.887466

H -1.255746 1.575865 1.312782

H -2.513236 2.473485 -0.460343

H -1.225450 -1.306392 -1.652199

H -2.470207 0.513078 -2.503752

H -1.520586 -0.507321 2.413674

H -3.656340 2.155234 0.842544

H -1.546339 -2.496595 0.361042

H -3.044728 0.355342 2.609225

H -3.612884 -0.811838 -2.282085

H -4.175272 -1.098182 1.025825

H -3.070460 -2.623852 -0.508870

H -3.219156 -2.157785 2.032400

H -4.429904 1.522646 -1.540258

H -4.614317 0.262187 -0.342750

Zero-point correction= 0.496724 Hartree/Particle

Thermal correction to Energy= 0.518340

Thermal correction to Enthalpy= 0.519284

Thermal correction to Gibbs Free Energy= 0.448679

Sum of electronic and zero-point Energies= -835.727918

Sum of electronic and thermal Energies= -835.706303

Sum of electronic and thermal Enthalpies= -835.705358

Sum of electronic and thermal Free Energies= -835.775963

The wave number of the imaginary frequency= -583.2252 cm^-1^

Entry **26** (TS1)

C -3.068985 0.433123 1.384942

C -2.931887 -0.044392 0.070269

C -3.546367 -1.387389 -0.260718

C -2.922677 0.973393 -1.048740

H -2.814291 1.458196 1.637536

H -3.295000 -0.242985 2.203941

H -3.073490 -1.818093 -1.148222

H -4.618859 -1.274492 -0.463445

H -3.427661 -2.090370 0.569364

H -2.670304 0.506327 -2.005785

H -2.192777 1.761728 -0.838508

H -3.910937 1.439344 -1.148817

O -1.172511 -0.656449 0.040692

N -0.231173 0.335878 0.045145

C 0.622448 0.249665 1.245649

C 1.417955 -1.083696 1.253379

C 2.155655 -1.345690 -0.051829

C 1.505548 1.509916 1.304026

C 0.568035 0.319672 -1.198274

C 1.368785 -1.007035 -1.310980

C 1.447448 1.583274 -1.225505

C 2.333961 1.709176 0.024452

H -0.143606 0.363560 -2.027284

H 0.781541 2.453433 -1.289190

H -0.058494 0.250641 2.102366

H 0.843951 2.373995 1.447053

H 0.634994 -1.812142 -1.455488

H 2.061145 1.578775 -2.134789

H 0.682617 -1.891019 1.374376

H 2.048040 -1.009453 -2.169331

H 2.159745 1.455615 2.182870

H 2.126650 -1.141067 2.085510

O 3.275183 -1.823926 -0.088624

H 2.816275 2.693934 0.041708

H 3.147776 0.975841 -0.016436

Zero-point correction= 0.305355 Hartree/Particle

Thermal correction to Energy= 0.320378

Thermal correction to Enthalpy= 0.321323

Thermal correction to Gibbs Free Energy= 0.263178

Sum of electronic and zero-point Energies= -674.091784

Sum of electronic and thermal Energies= -674.076761

Sum of electronic and thermal Enthalpies= -674.075817

Sum of electronic and thermal Free Energies= -674.133961

The wave number of the imaginary frequency= -562.5499 cm^-1^

Entry **26** (TS2)

C 1.630120 0.212132 -1.828079

C 1.847352 0.049070 -0.436339

C 2.507066 -1.347599 -0.089052

C 1.639341 -2.525539 -0.606671

C 3.883545 -1.474373 -0.796852

C 2.720142 -1.606220 1.417073

C 2.238364 1.374862 0.331216

C 3.757083 1.652086 0.195600

C 1.516145 2.593209 -0.304629

C 1.901069 1.389636 1.842078

H 1.269831 1.139161 -2.250322

H 1.668827 -0.631084 -2.506726

H 2.198477 -3.459633 -0.476897

H 1.393898 -2.444089 -1.669529

H 0.706099 -2.603868 -0.049297

H 4.309884 -2.460458 -0.575997

H 4.604977 -0.725540 -0.465039

H 3.790118 -1.390512 -1.884523

H 3.037103 -2.646627 1.556873

H 1.798778 -1.459813 1.986357

H 3.504506 -0.976482 1.846537

H 3.971812 2.657682 0.578030

H 4.083815 1.614707 -0.849003

H 4.364693 0.951228 0.775258

H 1.643928 3.466530 0.344114

H 0.446660 2.413269 -0.437093

H 1.940153 2.857445 -1.278942

H 2.098992 2.393547 2.235594

H 2.510579 0.697040 2.421692

H 0.854281 1.148361 2.025088

O 0.152326 -0.309539 0.224214

N -0.914412 0.363812 -0.343272

C -1.738623 -0.556185 -1.153370

C -2.377235 -1.667168 -0.275317

C -3.078960 -1.129764 0.963849

C -2.769112 0.281419 -1.932988

C -1.727235 1.033341 0.694578

C -2.346405 -0.000389 1.673175

C -2.770850 1.922172 -0.011219

C -3.642882 1.140870 -1.006933

H -1.051497 1.679916 1.259998

H -2.229118 2.712623 -0.546240

H -1.057435 -1.028869 -1.867315

H -2.217320 0.932725 -2.622806

H -1.518256 -0.456626 2.233372

H -3.394847 2.414221 0.745200

H -1.564210 -2.315581 0.077670

H -3.023289 0.467507 2.395014

H -3.390886 -0.384192 -2.544195

O -4.127490 -1.592559 1.375848

H -3.077563 -2.289356 -0.841945

H -4.245471 1.838846 -1.600711

H -4.358866 0.508668 -0.468519

Zero-point correction= 0.476672 Hartree/Particle

Thermal correction to Energy= 0.499061

Thermal correction to Enthalpy= 0.500005

Thermal correction to Gibbs Free Energy= 0.426973

Sum of electronic and zero-point Energies= -909.765633

Sum of electronic and thermal Energies= -909.743244

Sum of electronic and thermal Enthalpies= -909.742300

Sum of electronic and thermal Free Energies= -909.815333

The wave number of the imaginary frequency= -549.4949 cm^-1^

Entry **27** (TS1)

C 2.668858 -0.470356 1.506215

C 2.605183 -0.234165 0.125699

C 3.454263 0.881845 -0.443786

C 2.370807 -1.417308 -0.786406

H 2.213248 -1.353442 1.944156

H 3.033782 0.293087 2.186757

H 3.043729 1.226994 -1.397229

H 4.479335 0.530126 -0.617187

H 3.496180 1.733682 0.241822

H 1.519319 -2.006771 -0.432945

H 3.256049 -2.065645 -0.805645

H 2.167519 -1.090628 -1.811200

O 0.985992 0.692212 -0.036856

N -0.126083 -0.101753 0.097508

C -1.054536 0.481988 1.098564

C -1.660938 1.716752 0.375178

C -2.101614 -0.598164 1.426571

C -0.939850 -0.068079 -1.147701

C -1.583623 1.346877 -1.136284

C -1.978612 -1.199071 -1.042460

C -2.903361 -0.995401 0.172862

H -0.276286 -0.227049 -2.000375

H -1.439727 -2.150207 -0.948721

H -0.475635 0.747829 1.985675

H -1.573139 -1.472590 1.826587

H -0.940821 2.050947 -1.671976

H -2.568915 -1.249206 -1.966541

H -1.055046 2.602423 0.584897

H -2.563770 1.355860 -1.625470

H -2.776973 -0.237677 2.213144

H -2.683320 1.927718 0.707156

H -3.487930 -1.903317 0.364088

H -3.632405 -0.207072 -0.055419

Zero-point correction= 0.295530 Hartree/Particle

Thermal correction to Energy= 0.308862

Thermal correction to Enthalpy= 0.309806

Thermal correction to Gibbs Free Energy= 0.255557

Sum of electronic and zero-point Energies= -560.767505

Sum of electronic and thermal Energies= -560.754174

Sum of electronic and thermal Enthalpies= -560.753230

Sum of electronic and thermal Free Energies= -560.807478

The wave number of the imaginary frequency= -559.5692 cm^-1^

Entry **27** (TS2)

C 1.163702 -0.054409 -1.842366

C 1.516788 0.036199 -0.477713

C 2.340464 -1.217308 0.024755

C 1.806849 1.500722 0.042347

H 0.671372 0.752667 -2.365295

H 1.225393 -0.993126 -2.379334

C 2.670615 -1.207474 1.531879

C 3.676158 -1.321475 -0.758519

C 1.572352 -2.539981 -0.239431

C 0.900305 2.517702 -0.700708

C 3.269693 1.906248 -0.269908

C 1.575936 1.724566 1.556678

O -0.088947 -0.372426 0.397265

N -1.265672 0.097218 -0.170584

C -2.093986 -1.052448 -0.623032

C -2.610557 -1.700895 0.692090

C -3.227164 -0.474852 -1.490724

C -2.124510 0.694119 0.888208

C -2.623374 -0.525305 1.712746

C -3.267661 1.441961 0.177000

C -4.116799 0.484790 -0.679324

H -1.527034 1.391189 1.478610

H -2.826213 2.221703 -0.456102

H -1.462004 -1.722179 -1.209942

H -2.770643 0.058806 -2.333641

H -1.934579 -0.723124 2.538899

H -3.896279 1.947399 0.921576

H -1.926048 -2.488989 1.018210

H -3.614887 -0.350230 2.144475

H -3.829206 -1.291471 -1.909567

H -3.597814 -2.157760 0.562416

H -4.776734 1.051982 -1.346716

H -4.776916 -0.098488 -0.023947

H 0.651210 -2.584203 0.341232

H 1.318127 -2.682149 -1.294156

H 2.211451 -3.382629 0.050500

H 3.507197 -1.408073 -1.837024

H 4.333769 -0.466553 -0.590625

H 4.215536 -2.219782 -0.433374

H 3.402137 -0.438773 1.796223

H 1.773993 -1.065792 2.140358

H 3.112816 -2.173082 1.805134

H 0.583268 1.395147 1.862627

H 2.307634 1.212330 2.181325

H 1.669068 2.795741 1.772127

H 1.201306 2.650754 -1.745180

H -0.149178 2.213384 -0.681610

H 0.988766 3.497404 -0.218456

H 3.994679 1.374316 0.353410

H 3.522622 1.732144 -1.321436

H 3.395604 2.977382 -0.068602

Zero-point correction= 0.466398 Hartree/Particle

Thermal correction to Energy= 0.487269

Thermal correction to Enthalpy= 0.488213

Thermal correction to Gibbs Free Energy= 0.417798

Sum of electronic and zero-point Energies= -796.442840

Sum of electronic and thermal Energies= -796.421969

Sum of electronic and thermal Enthalpies= -796.421025

Sum of electronic and thermal Free Energies= -796.491439

The wave number of the imaginary frequency= -551.2769 cm^-1^

Entry **28** (TS1)

C -2.840409 -1.431240 0.102561

C -1.685967 -0.697244 -0.125146

C -1.694025 0.701540 -0.101199

C -2.856591 1.413594 0.152447

C -4.028900 0.680060 0.381913

C -4.020917 -0.719765 0.357489

C -0.300115 -1.168727 -0.412114

C -0.314173 1.195089 -0.379504

N 0.460845 0.021282 -0.545118

O 1.731266 0.041031 -0.933594

O 0.120960 -2.300068 -0.517199

O 0.104173 2.331109 -0.434927

H -2.821665 -2.516486 0.081738

H -2.850905 2.499088 0.167925

H -4.959201 1.204550 0.580948

H -4.944876 -1.261522 0.538997

C 4.189879 -0.171277 -0.517574

C 3.049574 0.061526 0.447178

C 2.858512 1.354026 0.958438

C 2.657823 -1.123355 1.298967

H 5.121955 -0.329449 0.039781

H 4.326287 0.687044 -1.181188

H 2.463705 -2.002942 0.681668

H 3.473830 -1.358957 1.995186

H 3.271135 2.220992 0.453764

H 1.765524 -0.911665 1.896916

H 2.188404 1.537198 1.792739

H 4.000543 -1.058749 -1.127629

Zero-point correction= 0.216070 Hartree/Particle

Thermal correction to Energy= 0.230867

Thermal correction to Enthalpy= 0.231811

Thermal correction to Gibbs Free Energy= 0.173146

Sum of electronic and zero-point Energies= -744.609085

Sum of electronic and thermal Energies= -744.594288

Sum of electronic and thermal Enthalpies= -744.593344

Sum of electronic and thermal Free Energies= -744.652009

The wave number of the imaginary frequency= -420.5947 cm^-1^

Entry **28** (TS2)

C -1.311248 -0.688760 1.550757

C -1.928055 -0.116105 0.408446

C -2.897261 -1.123500 -0.331589

C -2.230485 1.430339 0.501878

H -0.723535 -0.102044 2.243296

H -1.299520 -1.759024 1.705600

C -3.544539 -0.573606 -1.619620

C -4.034270 -1.543458 0.641286

C -2.166761 -2.429253 -0.743503

C -2.186411 2.193189 -0.845036

C -1.216473 2.144040 1.432880

C -3.626505 1.654170 1.139398

O -0.534703 -0.170563 -0.862736

N 0.744269 -0.113477 -0.471420

C 1.572722 1.011330 -0.706716

C 2.941145 0.569164 -0.313331

O 1.216493 2.087045 -1.138965

C 1.471108 -1.235485 -0.007762

C 2.880154 -0.761589 0.108002

O 1.009745 -2.326506 0.254670

C 4.011741 -1.436715 0.539557

H -1.324078 1.846002 2.481071

H -0.181696 1.984307 1.122476

H -1.400262 3.222514 1.387905

H -1.192701 2.152643 -1.290116

H -2.911678 1.829466 -1.571166

H -2.429261 3.244688 -0.648187

H -4.443523 1.349717 0.480257

H -3.731375 1.123593 2.092181

H -3.753828 2.724676 1.338836

H -4.239598 0.249411 -1.432853

H -2.790194 -0.242490 -2.337977

H -4.121186 -1.378334 -2.090885

H -1.449464 -2.249531 -1.543587

H -1.624256 -2.902507 0.076756

H -2.917465 -3.143886 -1.101435

H -3.640332 -2.026954 1.541302

H -4.663073 -0.708621 0.955491

H -4.681022 -2.269178 0.134302

C 5.224728 -0.733712 0.536550

H 3.952882 -2.471313 0.863064

C 5.285446 0.600003 0.115042

H 6.133087 -1.230741 0.865164

C 4.135154 1.274109 -0.318199

H 6.239663 1.119140 0.123811

H 4.169118 2.308040 -0.647336

Zero-point correction= 0.387502 Hartree/Particle

Thermal correction to Energy= 0.409606

Thermal correction to Enthalpy= 0.410550

Thermal correction to Gibbs Free Energy= 0.337702

Sum of electronic and zero-point Energies= -980.286655

Sum of electronic and thermal Energies= -980.264551

Sum of electronic and thermal Enthalpies= -980.263607

Sum of electronic and thermal Free Energies= -980.336454

The wave number of the imaginary frequency= -339.4238 cm^-1^

Entry **29** (TS1)

C 1.733118 0.698816 -0.017914

C 0.337392 1.249834 0.181716

N -0.489820 0.006200 -0.013619

C 0.331601 -1.246287 0.175185

C 1.728981 -0.696599 -0.029459

C 2.926656 1.403777 -0.167496

O -1.667410 0.003110 0.661659

C 2.918661 -1.405921 -0.189665

C -0.033141 2.286919 -0.890708

H -1.070583 2.610711 -0.765862

H 0.607025 3.173150 -0.809189

H 0.086388 1.864259 -1.892984

C 0.161808 1.862564 1.586146

H -0.886516 2.122766 1.752288

H 0.471627 1.165239 2.369870

H 0.771966 2.768162 1.677466

C 0.172713 -1.841129 1.591449

H 0.794634 -2.737879 1.690976

H 0.480519 -1.128921 2.362094

H -0.870056 -2.114219 1.773339

C -0.033473 -2.309491 -0.871126

H 0.643782 -3.167865 -0.793147

H -1.051042 -2.677215 -0.708976

H 0.037255 -1.902789 -1.883787

C -3.209480 0.069181 -0.285883

C -3.528758 1.452896 -0.305753

H -3.255788 2.081398 -1.147608

H -3.942733 1.941881 0.570950

C -2.895767 -0.553865 -1.628598

C -4.004220 -0.786052 0.682659

H -1.948472 -0.158624 -2.011939

H -2.808978 -1.641122 -1.561361

H -3.686553 -0.320992 -2.350792

H -3.555941 -1.778291 0.787126

H -4.038639 -0.318387 1.671845

H -5.034998 -0.907795 0.326401

C 4.116799 -0.702202 -0.336413

C 4.120869 0.695922 -0.325094

H 2.934140 2.491031 -0.162995

H 5.057117 1.235316 -0.442270

H 5.049850 -1.245119 -0.462367

H 2.921463 -2.493147 -0.201291

Zero-point correction= 0.364779 Hartree/Particle

Thermal correction to Energy= 0.384125

Thermal correction to Enthalpy= 0.385069

Thermal correction to Gibbs Free Energy= 0.318526

Sum of electronic and zero-point Energies= -753.647392

Sum of electronic and thermal Energies= -753.628046

Sum of electronic and thermal Enthalpies= -753.627102

Sum of electronic and thermal Free Energies= -753.693645

The wave number of the imaginary frequency= -554.9969 cm^-1^

Entry **29** (TS2)

C -2.058083 -1.173268 1.456568

C -2.193308 -0.263442 0.356660

C -3.001501 -0.927546 -0.845453

C -2.499777 1.216859 0.826042

H -1.788491 -0.842683 2.449023

H -2.120057 -2.244339 1.310311

C -3.214350 -0.015435 -2.072243

C -4.397683 -1.394849 -0.354464

C -2.290884 -2.197828 -1.382742

C -2.324403 2.286737 -0.278908

C -1.573633 1.587185 2.019413

C -3.949231 1.350536 1.360267

O -0.607398 -0.262150 -0.442782

N 0.581589 0.061410 0.204541

C 1.395120 1.089084 -0.567781

C 2.811777 0.600845 -0.338697

C 1.077174 1.103190 -2.077205

C 1.238007 2.499276 0.032596

C 1.430853 -1.148375 0.547274

C 2.831671 -0.634817 0.297036

C 1.153772 -2.374291 -0.343052

C 1.241721 -1.520491 2.028476

H 0.224902 -1.872530 2.217625

H 0.152769 -2.770472 -0.170119

H 1.438074 -0.656704 2.670800

H 1.932056 -2.324267 2.311014

H 0.222745 2.881278 -0.073866

H 1.250101 -2.128528 -1.404658

H 0.059726 1.449342 -2.273058

H 1.881726 -3.159994 -0.110564

H 1.190064 0.107132 -2.514552

H 1.498671 2.493265 1.094968

C 4.043518 -1.257399 0.599714

H 1.911227 3.198090 -0.478165

H 1.772326 1.779233 -2.587857

H -1.900742 1.111135 2.949862

H -0.536169 1.305588 1.834316

H -1.619772 2.668237 2.190834

H -1.458887 2.084482 -0.905953

H -3.198604 2.359431 -0.927855

H -2.193381 3.272355 0.181627

H -4.698140 1.322093 0.563893

H -4.188201 0.570571 2.091021

H -4.051599 2.321122 1.862083

H -3.935506 0.783609 -1.884067

H -2.277572 0.427964 -2.417186

H -3.618967 -0.618494 -2.893858

H -1.354147 -1.953589 -1.881640

H -2.079848 -2.934564 -0.602284

H -2.953738 -2.684737 -2.107974

H -4.320859 -2.120944 0.461385

H -5.029918 -0.574251 -0.012700

H -4.920335 -1.884613 -1.185177

C 5.238927 -0.626016 0.251400

H 4.063248 -2.223301 1.098817

C 5.219142 0.616364 -0.391307

H 6.188705 -1.101770 0.481199

C 4.004150 1.237294 -0.687384

H 6.153581 1.102163 -0.659651

H 3.995205 2.204345 -1.184382

Zero-point correction= 0.536589 Hartree/Particle

Thermal correction to Energy= 0.563040

Thermal correction to Enthalpy= 0.563985

Thermal correction to Gibbs Free Energy= 0.483914

Sum of electronic and zero-point Energies= -989.311843

Sum of electronic and thermal Energies= -989.285392

Sum of electronic and thermal Enthalpies= -989.284448

Sum of electronic and thermal Free Energies= -989.364518

The wave number of the imaginary frequency= -510.1703 cm^-1^

Entry **30** (TS1)

C -3.360155 -1.181329 0.997558

C -3.330742 -0.063620 0.123154

C -4.113876 -0.209323 -1.170523

C -3.467434 1.299109 0.772876

H -3.052690 -1.094934 2.034671

H -3.572719 -2.177010 0.620328

H -3.787247 0.536963 -1.901242

H -5.184288 -0.063793 -0.981961

H -3.972851 -1.202285 -1.607545

H -3.241952 2.098049 0.060482

H -2.806674 1.397750 1.637908

H -4.498190 1.441172 1.122651

O -1.750604 -0.036246 -0.681441

N -0.578513 -0.008309 0.020067

C 0.242531 1.250540 -0.192662

C 1.641142 0.701707 0.015286

C 0.033583 1.802850 -1.637163

C -0.108317 2.348191 0.841997

C 0.258007 -1.257018 -0.173376

C 1.648589 -0.695645 0.033941

C 0.067755 -1.831042 -1.612539

C -0.119304 -2.334340 0.871439

H -1.144803 -2.661064 0.660963

H -1.000435 -2.031641 -1.738858

C -0.003046 -1.923224 2.340598

H 0.518825 -3.209506 0.698863

H -1.114473 2.720692 0.620194

H 0.315728 -1.042449 -2.330403

H -1.037887 1.991973 -1.757923

C 0.874748 -3.083463 -1.975372

H 0.282361 1.007039 -2.346378

C -0.013826 1.947637 2.315776

C 2.841265 -1.390852 0.243422

H 0.566106 3.195409 0.667686

C 0.822103 3.059616 -2.024799

C 4.031312 -0.678756 0.413148

H 2.850194 -2.476247 0.288794

C 4.024511 0.717903 0.392594

H 4.962987 -1.214862 0.573641

C 2.827586 1.413647 0.203943

H 4.950907 1.267610 0.536793

H 2.827688 2.499837 0.219704

H -0.702526 1.133344 2.554237

H 0.995697 1.612572 2.576430

H -0.259535 2.801681 2.957857

H 0.551761 3.930587 -1.418285

H 1.903578 2.908577 -1.940317

H 0.610975 3.316629 -3.069465

H 1.953559 -2.919949 -1.880110

H 0.606859 -3.950272 -1.361873

H 0.678650 -3.355439 -3.019221

H 1.018302 -1.620857 2.595779

H -0.665644 -1.085355 2.569626

H -0.276421 -2.761610 2.992244

Zero-point correction= 0.480220 Hartree/Particle

Thermal correction to Energy= 0.504806

Thermal correction to Enthalpy= 0.505750

Thermal correction to Gibbs Free Energy= 0.427567

Sum of electronic and zero-point Energies= -910.774578

Sum of electronic and thermal Energies= -910.749992

Sum of electronic and thermal Enthalpies= -910.749048

Sum of electronic and thermal Free Energies= -910.827232

The wave number of the imaginary frequency= -599.0933 cm^-1^

Entry **30** (TS2)

C 2.321106 1.254110 1.371560

C 2.472767 0.283651 0.324917

C 3.286870 0.885813 -0.907403

C 2.809223 -1.162477 0.875972

H 2.002473 0.983195 2.367827

H 2.409614 2.314296 1.170002

C 3.514449 -0.089669 -2.082344

C 4.677005 1.389454 -0.434096

C 2.576148 2.118928 -1.522015

C 2.672063 -2.298871 -0.166643

C 1.879049 -1.484851 2.075573

C 4.254041 -1.234578 1.435883

O 0.893925 0.214594 -0.471400

N -0.327122 -0.025716 0.178890

C -1.137380 -1.109716 -0.544952

C -2.552780 -0.611573 -0.335508

C -0.750655 -1.184303 -2.056965

C -0.944652 -2.501673 0.116769

C -1.175273 1.234163 0.343320

C -2.572936 0.669804 0.208958

C -0.891737 2.281286 -0.777944

C -0.940183 1.892454 1.724548

H 0.082885 2.282562 1.747627

H 0.127401 2.642622 -0.637040

C -1.183693 1.017884 2.956311

H -1.593598 2.771937 1.785778

H 0.122395 -2.728630 0.124483

H -0.904983 1.766162 -1.743554

H 0.267440 -1.579694 -2.122499

C -1.837419 3.486548 -0.864341

H -0.700309 -0.165227 -2.450798

C -1.516882 -2.685161 1.527418

C -3.786833 1.273388 0.549456

H -1.401865 -3.251955 -0.540974

C -1.662640 -2.009773 -2.974050

H 2.176087 -0.942886 2.979442

H 0.840994 -1.242329 1.853573

H 1.948197 -2.551667 2.314551

H 1.815874 -2.152478 -0.821822

H 3.560578 -2.396183 -0.792506

H 2.547190 -3.257063 0.350105

H 5.013884 -1.241594 0.649833

H 4.469086 -0.408000 2.121470

H 4.365944 -2.171169 1.996688

H 4.248041 -0.865702 -1.851959

H 2.584530 -0.565431 -2.401875

H 3.909413 0.474217 -2.935821

H 1.660418 1.835865 -2.038488

H 2.330418 2.886293 -0.782712

H 3.253215 2.582519 -2.249347

H 4.592867 2.155998 0.343189

H 5.315666 0.593455 -0.050132

H 5.198143 1.840521 -1.287259

C -4.982685 0.593008 0.312959

H -3.807678 2.260896 1.001232

C -4.963799 -0.687764 -0.246072

H -5.929694 1.058645 0.572798

C -3.748941 -1.299312 -0.560786

H -5.896217 -1.217723 -0.421916

H -3.741564 -2.309091 -0.960441

H -1.170797 -1.906286 2.211198

H -2.610616 -2.661077 1.530152

H -1.203416 -3.653551 1.934869

H -1.745106 -3.056787 -2.661074

H -2.673079 -1.593411 -3.030032

H -1.251431 -2.010191 -3.990322

H -2.867457 3.188581 -1.082884

H -1.845498 4.084110 0.054099

H -1.508784 4.148324 -1.674421

H -2.199069 0.607525 2.966106

H -0.483644 0.179958 2.997724

H -1.054884 1.609090 3.870939

Zero-point correction= 0.652069 Hartree/Particle

Thermal correction to Energy= 0.683838

Thermal correction to Enthalpy= 0.684782

Thermal correction to Gibbs Free Energy= 0.593510

Sum of electronic and zero-point Energies= -1146.434836

Sum of electronic and thermal Energies= -1146.403067

Sum of electronic and thermal Enthalpies= -1146.402123

Sum of electronic and thermal Free Energies= -1146.493395

The wave number of the imaginary frequency= -492.7431 cm^-1^

Entry **31** (TS1)

C -0.317747 -1.265487 -0.454993

C 0.211839 0.021530 -0.281554

C -0.697615 1.097671 -0.069459

C -2.061735 0.817543 0.044666

C -2.597879 -0.474997 -0.062817

C -1.687359 -1.501009 -0.338322

C -0.169815 2.466721 -0.076246

C -1.122914 3.636379 -0.093339

C 1.158371 2.656970 -0.109829

C 2.199727 1.562177 0.002608

C 2.699798 1.562747 1.471707

C 3.363633 1.865951 -0.959493

N 1.569033 0.275237 -0.372342

O 2.377472 -0.732675 -0.817167

C -4.112374 -0.700316 0.087890

C -4.493463 -2.187946 -0.034887

C -4.867395 0.081230 -1.013853

C -4.577941 -0.200318 1.476195

H 0.343789 -2.078462 -0.712876

H -2.735416 1.652676 0.208247

H -2.034202 -2.520239 -0.472138

H -1.740682 3.663808 0.813739

H -1.810531 3.583744 -0.946679

H -0.577295 4.582679 -0.153169

H 1.562022 3.667015 -0.146560

H 3.537842 0.878392 1.626420

H 1.886396 1.286185 2.149448

H 3.038599 2.569499 1.743597

H 4.161543 1.126440 -0.874265

H 3.780810 2.851231 -0.723272

H 3.010541 1.877254 -1.994924

H -5.575892 -2.305649 0.091471

H -4.001689 -2.797603 0.731862

H -4.230132 -2.596588 -1.017142

H -5.950750 -0.064420 -0.917057

H -4.565432 -0.260276 -2.010571

H -4.669835 1.157104 -0.956708

H -5.658417 -0.349558 1.596340

H -4.372508 0.866547 1.614771

H -4.067621 -0.744543 2.279169

C 4.487179 -1.479900 0.313055

C 3.016577 -1.873760 0.194921

C 2.301833 -1.949848 1.453330

C 2.864983 -3.104160 -0.696494

H 4.632834 -0.600237 0.943519

H 4.903480 -1.270362 -0.677482

H 2.555238 -1.296963 2.281514

H 5.059366 -2.303746 0.756138

H 1.421755 -2.574319 1.558730

H 3.174702 -2.867257 -1.719362

H 1.834203 -3.467369 -0.724327

H 3.498056 -3.917307 -0.323229

Zero-point correction= 0.456162 Hartree/Particle

Thermal correction to Energy= 0.480085

Thermal correction to Enthalpy= 0.481029

Thermal correction to Gibbs Free Energy= 0.404822

Sum of electronic and zero-point Energies= -909.594114

Sum of electronic and thermal Energies= -909.570191

Sum of electronic and thermal Enthalpies= -909.569247

Sum of electronic and thermal Free Energies= -909.645454

The wave number of the imaginary frequency= -322.6716 cm^-1^

Entry **31** (TS2)

C -0.905545 -0.898036 -0.745268

C -0.717305 0.404788 -0.283457

C -1.845029 1.130109 0.181928

C -3.084643 0.487387 0.243896

C -3.281465 -0.837609 -0.177262

C -2.162807 -1.504026 -0.688301

C -1.661771 2.548079 0.524352

C -0.545956 3.157586 0.090852

C 0.529035 2.455794 -0.725577

N 0.552201 1.037534 -0.234908

O 1.567528 0.266750 -0.773816

H -0.060768 -1.435438 -1.152224

H -3.931080 1.052409 0.621855

C -4.677126 -1.478912 -0.080421

H -2.251644 -2.519658 -1.058541

C 2.197022 0.583594 2.484047

C 2.497830 -0.690948 1.647258

C 2.950498 -0.311670 0.177207

C 3.845354 0.813145 0.152994

C 3.434726 -1.422225 -0.861305

C 3.342000 -0.905488 -2.324167

C 4.932000 -1.763314 -0.619405

C 2.660633 -2.755333 -0.820414

C 3.653805 -1.427009 2.374424

C 1.239588 -1.578757 1.772154

H 3.877394 1.548141 0.942766

H 1.483138 1.236425 1.977955

H 3.098806 1.154090 2.727800

H 4.433848 1.036658 -0.728205

H 1.754120 0.278235 3.438817

H 0.340872 -1.069392 1.429971

H 1.096834 -1.817109 2.833275

H 3.814258 0.071316 -2.462607

H 4.600255 -0.881395 2.298200

H 2.309366 -0.825413 -2.663838

H 3.402246 -1.511079 3.438643

H 5.582593 -0.895930 -0.765101

H 1.316131 -2.526435 1.242123

H 3.807324 -2.442649 1.998615

H 5.124832 -2.161488 0.377962

H 1.584379 -2.614888 -0.935312

H 3.865058 -1.616102 -2.975537

H 2.841525 -3.320413 0.098381

H 5.239492 -2.528541 -1.342572

H 3.001030 -3.383857 -1.652283

C -5.140456 -1.487171 1.395884

C -5.685612 -0.665624 -0.926572

C -4.687193 -2.931802 -0.592580

H -4.400796 -2.993537 -1.648759

H -4.008503 -3.571415 -0.016820

H -5.695620 -3.350823 -0.499251

H -5.757367 0.373369 -0.587746

H -5.389336 -0.652122 -1.981724

H -6.687653 -1.107679 -0.859778

H -4.452292 -2.070835 2.018001

H -5.188911 -0.475391 1.812512

H -6.139688 -1.931944 1.483299

C 0.208744 2.509939 -2.238706

C 1.865495 3.158268 -0.471063

H -0.388764 4.216204 0.288746

C -2.724850 3.287356 1.296412

H -3.668712 3.337335 0.737916

H -2.944853 2.793063 2.251079

H -2.409890 4.313606 1.507900

H -0.760410 2.049688 -2.452793

H 0.176428 3.549099 -2.584667

H 0.981672 1.978716 -2.804381

H 1.777675 4.203332 -0.789499

H 2.127218 3.150627 0.590515

H 2.672332 2.695754 -1.041263

Zero-point correction= 0.627404 Hartree/Particle

Thermal correction to Energy= 0.658647

Thermal correction to Enthalpy= 0.659591

Thermal correction to Gibbs Free Energy= 0.569087

Sum of electronic and zero-point Energies= -1145.263735

Sum of electronic and thermal Energies= -1145.232492

Sum of electronic and thermal Enthalpies= -1145.231547

Sum of electronic and thermal Free Energies= -1145.322052

The wave number of the imaginary frequency= -444.1660 cm^-1^

Entry **32** (TS1)

C -3.688006 0.923854 -0.712336

C -2.349102 0.509039 -0.717628

C -1.396992 1.302199 -0.033218

C -1.795635 2.484951 0.607870

C -3.130206 2.878941 0.578107

C -4.084784 2.098288 -0.077987

C -1.880814 -0.701926 -1.408571

N -0.052067 0.898643 -0.065885

C 0.218056 -0.580325 0.034624

O 0.811513 1.689992 0.627304

C 1.731675 -0.810337 -0.170684

C 2.296069 -0.716862 -1.453266

C 3.661325 -0.912771 -1.659266

C 4.498323 -1.208220 -0.581171

C 3.951695 -1.303129 0.697583

C 2.582985 -1.108157 0.899220

C -0.249638 -1.116384 1.420121

C -0.285846 -0.280003 2.545973

C -0.681401 -0.772250 3.790993

C -1.042917 -2.110891 3.940940

C -1.004593 -2.953512 2.830200

C -0.614673 -2.460382 1.584384

C -0.620133 -1.246332 -1.077666

C -2.648935 -1.329469 -2.405618

C -0.201953 -2.426408 -1.705559

C -2.217029 -2.495343 -3.027339

C -0.990704 -3.054931 -2.668129

H -4.437378 0.306340 -1.197904

H -1.051067 3.064754 1.136775

H -3.427892 3.790964 1.089265

H -5.129776 2.394294 -0.086142

H 1.657022 -0.507627 -2.304510

H 4.068023 -0.839456 -2.664632

H 5.562002 -1.366292 -0.738555

H 4.588545 -1.532438 1.547979

H 2.180251 -1.187413 1.901930

H 0.005792 0.759600 2.449927

H -0.706782 -0.101009 4.645735

H -1.352763 -2.492297 4.910374

H -1.285147 -3.999310 2.926931

H -0.605893 -3.132657 0.733444

H -3.592353 -0.886574 -2.708270

H 0.756135 -2.860500 -1.441601

H -2.832134 -2.961029 -3.792662

H -0.641847 -3.970010 -3.138722

C 1.368266 2.717699 -1.770748

C 1.685756 2.982932 -0.316585

C 1.141588 4.184031 0.219770

C 3.094339 2.638014 0.129138

H 2.005414 3.354559 -2.397633

H 0.324493 2.947990 -2.002439

H 3.382600 1.637008 -0.195494

H 3.806364 3.359510 -0.293038

H 0.295048 4.675371 -0.249194

H 3.172652 2.684910 1.219828

H 1.488183 4.577751 1.170989

H 1.562550 1.679217 -2.038531

Zero-point correction= 0.467736 Hartree/Particle

Thermal correction to Energy= 0.493643

Thermal correction to Enthalpy= 0.494587

Thermal correction to Gibbs Free Energy= 0.412243

Sum of electronic and zero-point Energies= -1250.125719

Sum of electronic and thermal Energies= -1250.099812

Sum of electronic and thermal Enthalpies= -1250.098868

Sum of electronic and thermal Free Energies= -1250.181211

The wave number of the imaginary frequency= -536.4838 cm^-1^

Entry **32** (TS2)

C 2.459256 -3.138612 -1.200988

C 1.881456 -1.873270 -1.039365

C 0.667544 -1.763444 -0.316560

C 0.094552 -2.914013 0.243166

C 0.685150 -4.162554 0.056765

C 1.866014 -4.284142 -0.674710

C 2.504485 -0.641654 -1.542118

N 0.083064 -0.483809 -0.235204

C 1.058100 0.617296 0.143956

O -1.140604 -0.415476 0.422404

C 0.333186 1.984239 0.199746

C 0.063991 2.713941 -0.969923

C -0.519802 3.979448 -0.918426

C -0.873569 4.542439 0.307750

C -0.636617 3.821808 1.477196

C -0.032657 2.563667 1.422615

C 1.728223 0.306534 1.515808

C 1.041924 -0.407019 2.509788

C 1.629380 -0.658565 3.750426

C 2.916375 -0.198314 4.028916

C 3.607436 0.517053 3.051666

C 3.020016 0.764610 1.809879

C 2.140643 0.594716 -0.964374

C 3.479307 -0.662618 -2.555154

C 2.837512 1.748018 -1.349313

C 4.139049 0.495607 -2.948386

C 3.830943 1.705706 -2.326832

H 3.405861 -3.225738 -1.725495

H -0.789286 -2.816508 0.852100

H 0.227035 -5.039226 0.507631

H 2.335942 -5.253666 -0.811937

H 0.326531 2.300263 -1.935926

H -0.701909 4.521428 -1.842833

H -1.327055 5.529199 0.350494

H -0.908122 4.240652 2.442648

H 0.163618 2.036750 2.348444

H 0.037050 -0.759775 2.310959

H 1.075657 -1.218899 4.499613

H 3.375067 -0.396426 4.994079

H 4.612158 0.882039 3.249256

H 3.584037 1.312770 1.063551

H 3.715961 -1.601580 -3.045552

H 2.600098 2.697983 -0.884665

H 4.890519 0.453380 -3.732220

H 4.350884 2.618129 -2.605614

C -2.938690 0.221485 -1.560137

C -2.650400 -0.821428 -0.395424

C -2.477313 -2.140001 -0.956016

C -3.529392 -0.815325 0.935601

C -4.188547 -0.205183 -2.383489

C -1.776390 0.231991 -2.589364

C -3.409923 0.470565 1.793895

C -5.026977 -1.024270 0.605465

H -2.065041 -2.276716 -1.946447

C -3.127696 -1.980125 1.880274

H -2.698354 -3.043272 -0.407228

C -3.189228 1.661677 -1.074809

H -4.176341 1.764120 -0.614418

H -2.435273 1.999939 -0.366379

H -3.171575 2.341194 -1.934480

H -4.101922 -1.230470 -2.756335

H -5.124174 -0.123334 -1.829017

H -4.268797 0.458557 -3.253504

H -0.802831 0.365337 -2.125534

H -1.739244 -0.691582 -3.176339

H -1.944733 1.048777 -3.300491

H -3.932993 0.302218 2.742977

H -2.371145 0.714998 2.016337

H -3.872319 1.339796 1.328353

H -3.313325 -2.970580 1.453957

H -2.080470 -1.915321 2.179539

H -3.735106 -1.916899 2.789922

H -5.479546 -0.140775 0.148761

H -5.188768 -1.879906 -0.058913

H -5.572753 -1.217964 1.536742

Zero-point correction= 0.639610 Hartree/Particle

Thermal correction to Energy= 0.672640

Thermal correction to Enthalpy= 0.673584

Thermal correction to Gibbs Free Energy= 0.578095

Sum of electronic and zero-point Energies= -1485.785526

Sum of electronic and thermal Energies= -1485.752496

Sum of electronic and thermal Enthalpies= -1485.751552

Sum of electronic and thermal Free Energies= -1485.847041

The wave number of the imaginary frequency= -400.5258 cm^-1^

Entry **33** (TS1)

C -2.444518 0.126197 -0.810399

C -1.193472 -0.410676 -0.485993

C -1.140769 -1.666241 0.146768

C -2.306555 -2.351531 0.467515

C -3.550051 -1.797506 0.155653

C -3.614823 -0.559542 -0.484748

O 0.054952 -2.270020 0.473272

C 1.224238 -1.615127 0.149006

C 1.224944 -0.359262 -0.484253

C 2.452047 0.226743 -0.812503

N 0.001779 0.244945 -0.827570

O -0.032855 1.606412 -0.913410

C 3.650180 -0.409418 -0.486683

C 3.636950 -1.647724 0.156064

C 2.417698 -2.252075 0.469068

H -2.476776 1.080751 -1.321216

H -2.218658 -3.311796 0.965984

H -4.457863 -2.335726 0.410962

H -4.576615 -0.124801 -0.740949

H 2.444928 1.181121 -1.324698

H 4.592872 0.063963 -0.745303

H 4.566363 -2.147795 0.411222

H 2.369628 -3.215252 0.967354

C -1.284355 3.389885 0.228739

C -0.076899 2.499412 0.501758

C -0.207247 1.646740 1.663639

C 1.236760 3.271870 0.427191

H -2.219728 2.833939 0.335213

H -1.234559 3.796027 -0.786854

H -1.183032 1.344513 2.028971

H -1.301064 4.230743 0.932625

H 0.668051 1.226161 2.148441

H 1.372730 3.695797 -0.573150

H 2.091276 2.628417 0.651492

H 1.228456 4.097711 1.149078

Zero-point correction= 0.281266 Hartree/Particle

Thermal correction to Energy= 0.297572

Thermal correction to Enthalpy= 0.298516

Thermal correction to Gibbs Free Energy= 0.236172

Sum of electronic and zero-point Energies= -824.126830

Sum of electronic and thermal Energies= -824.110524

Sum of electronic and thermal Enthalpies= -824.109580

Sum of electronic and thermal Free Energies= -824.171924

The wave number of the imaginary frequency= -294.4849 cm^-1^

Entry **33** (TS2)

C -1.123003 -2.223912 -1.197090

C -1.471010 -1.106619 -0.436048

C -2.731730 -1.066477 0.183259

C -3.612073 -2.133345 0.087660

C -3.238296 -3.270761 -0.636602

C -2.002858 -3.307462 -1.281231

O -3.101597 0.055068 0.910190

C -2.587274 1.251207 0.433701

C -1.329346 1.265543 -0.198184

C -0.834510 2.472485 -0.696257

N -0.650851 0.030535 -0.259953

O 0.601101 -0.002166 -0.797192

C -1.565624 3.651193 -0.515285

C -2.794821 3.630205 0.140313

C -3.317071 2.416498 0.603847

H -0.167107 -2.234254 -1.705995

H -4.575156 -2.061152 0.582993

H -3.919346 -4.113849 -0.703308

H -1.716005 -4.179545 -1.861873

H 0.107991 2.472668 -1.226497

H -1.166551 4.586835 -0.896595

H -3.359161 4.547005 0.282321

H -4.281502 2.360760 1.098674

C 0.908761 -0.533232 2.495836

C 1.949309 -0.841401 1.382848

C 1.960658 0.293860 0.273982

C 1.735156 1.605802 0.824434

C 3.076079 0.327738 -0.864179

C 2.643737 1.246236 -2.041089

C 4.401037 0.926529 -0.320431

C 3.400536 -1.045679 -1.488065

C 3.325041 -0.902085 2.095712

C 1.621736 -2.266516 0.881940

H 1.136971 1.767031 1.709103

H -0.080747 -0.306548 2.092780

H 1.220323 0.293511 3.142195

H 2.060353 2.497201 0.304241

H 0.812593 -1.417284 3.135813

H 0.576872 -2.364748 0.591507

H 1.797063 -2.968159 1.706530

H 2.414580 2.266821 -1.719386

H 3.656740 0.086695 2.428573

H 1.775382 0.851466 -2.568367

H 3.230896 -1.538093 2.983996

H 4.258116 1.928938 0.094715

H 2.237082 -2.592140 0.045393

H 4.106285 -1.339850 1.467920

H 4.868729 0.309421 0.447340

H 2.504117 -1.535988 -1.876187

H 3.476063 1.316170 -2.751082

H 3.895551 -1.721456 -0.785622

H 5.114033 1.011486 -1.149257

H 4.089150 -0.898732 -2.328670

Zero-point correction= 0.452764 Hartree/Particle

Thermal correction to Energy= 0.476394

Thermal correction to Enthalpy= 0.477338

Thermal correction to Gibbs Free Energy= 0.401813

Sum of electronic and zero-point Energies= -1059.795867

Sum of electronic and thermal Energies= -1059.772237

Sum of electronic and thermal Enthalpies= -1059.771293

Sum of electronic and thermal Free Energies= -1059.846818

The wave number of the imaginary frequency= -426.0489 cm^-1^

Entry **34** (TS1)

C 1.340676 -0.559831 -0.197979

N 0.068743 0.023354 -0.296791

C 2.486776 0.240445 -0.371797

C 1.512066 -1.940336 0.035669

C 2.791007 -2.479731 0.137079

C 3.758003 -0.319277 -0.266457

C 3.926730 -1.678476 -0.001632

H 0.651396 -2.591824 0.122372

H 2.896113 -3.546524 0.318447

H 2.364320 1.285490 -0.617063

H 4.626166 0.320683 -0.403916

H 4.920531 -2.108669 0.080773

O 0.005272 1.286084 -0.787196

C -1.169588 -0.661178 -0.179746

C -1.458866 -1.457071 0.938871

C -2.138163 -0.502135 -1.182404

C -3.371437 -1.138269 -1.067942

C -2.691771 -2.102882 1.035817

C -3.653716 -1.946967 0.036559

H -1.909593 0.125633 -2.036600

H -4.113055 -1.010289 -1.852155

H -0.725299 -1.555553 1.732643

H -2.904460 -2.716741 1.907095

H -4.614854 -2.446445 0.118829

C -0.042156 3.713045 -0.639686

C -0.200613 2.590113 0.370797

C 0.828083 2.477913 1.351958

C -1.623906 2.412557 0.874233

H -0.330788 4.669294 -0.187735

H 0.993364 3.793691 -0.983914

H 1.811017 2.906372 1.186365

H -0.682159 3.537189 -1.509812

H 0.694984 1.863356 2.236882

H -2.325538 2.305522 0.042654

H -1.916691 3.293504 1.459673

H -1.717587 1.535644 1.520523

Zero-point correction= 0.298717 Hartree/Particle

Thermal correction to Energy= 0.315426

Thermal correction to Enthalpy= 0.316370

Thermal correction to Gibbs Free Energy= 0.253260

Sum of electronic and zero-point Energies= -750.089392

Sum of electronic and thermal Energies= -750.072683

Sum of electronic and thermal Enthalpies= -750.071739

Sum of electronic and thermal Free Energies= -750.134849

The wave number of the imaginary frequency= -508.8806 cm^-1^

Entry **34** (TS2)

C -1.539631 -1.177089 -0.239383

N -0.868410 0.066703 0.033459

C -2.161861 -1.890239 0.790756

C -1.565470 -1.682786 -1.546219

C -2.209038 -2.888999 -1.814421

C -2.820827 -3.089701 0.512189

C -2.844229 -3.593900 -0.788205

H -1.073041 -1.125709 -2.335967

H -2.223804 -3.275217 -2.830233

H -2.114668 -1.513606 1.807430

H -3.302182 -3.636237 1.318722

H -3.349742 -4.531690 -1.001449

O 0.336432 0.162666 -0.629863

C -1.636173 1.264267 0.073366

C -2.921796 1.271755 0.648848

C -1.115456 2.469693 -0.422954

C -1.853394 3.649795 -0.325776

C -3.646574 2.456729 0.735039

C -3.120038 3.658686 0.255663

H -0.148785 2.465136 -0.902521

H -1.428963 4.569329 -0.721599

H -3.358126 0.354081 1.024223

H -4.636152 2.435972 1.184756

H -3.690249 4.580231 0.329379

C 1.958304 -0.885972 1.269476

C 1.841912 0.373422 0.315514

C 1.635170 1.605480 1.015581

C 2.812312 0.555094 -0.932332

C 3.394736 -1.000677 1.842244

C 1.026942 -0.723748 2.502899

H 1.108349 1.654069 1.957682

C 1.615157 -2.247248 0.621266

H 1.870228 2.557936 0.558151

C 3.061354 -0.734270 -1.742029

C 4.187177 1.110084 -0.474255

C 2.247610 1.593162 -1.940003

H 1.326547 1.245737 -2.407851

H 2.054061 2.567786 -1.482274

H 2.993282 1.755487 -2.727192

H 3.675794 -1.458648 -1.200652

H 2.123454 -1.213616 -2.032410

H 3.606968 -0.478879 -2.658423

H 4.085067 2.057584 0.064170

H 4.738095 0.419070 0.164850

H 4.805558 1.297517 -1.360509

H 0.606073 -2.271904 0.214733

H 2.305659 -2.528808 -0.172364

H 1.682502 -3.023679 1.392939

H 0.010044 -0.448523 2.216755

H 0.985023 -1.675004 3.044747

H 1.403094 0.026875 3.205618

H 4.122165 -1.331789 1.095700

H 3.739527 -0.053813 2.270611

H 3.394527 -1.749163 2.644078

Zero-point correction= 0.470083 Hartree/Particle

Thermal correction to Energy= 0.494182

Thermal correction to Enthalpy= 0.495126

Thermal correction to Gibbs Free Energy= 0.417389

Sum of electronic and zero-point Energies= -985.758428

Sum of electronic and thermal Energies= -985.734329

Sum of electronic and thermal Enthalpies= -985.733385

Sum of electronic and thermal Free Energies= -985.811122

The wave number of the imaginary frequency= -451.6900 cm^-1^

Entry **35** (TS1)

C 2.189492 0.483453 -1.205377

C 1.215681 0.281103 -0.215957

C 1.529604 -0.537329 0.874508

C 2.777867 -1.158509 0.955936

C 3.761692 -0.973543 -0.023897

C 3.431875 -0.129303 -1.100663

C 5.146599 -1.640900 0.038627

C 5.340786 -2.540102 -1.205682

C 6.245640 -0.551914 0.058303

C 5.317656 -2.516036 1.294649

N -0.037749 0.937883 -0.322062

O -0.004335 2.211461 -0.787700

C -1.295080 0.322949 -0.240788

C -2.462185 1.085599 -0.413128

C -3.720593 0.491029 -0.326078

C -3.887967 -0.876816 -0.079653

C -2.708095 -1.629741 0.056175

C -1.442804 -1.062704 -0.025449

C -5.262797 -1.557755 0.028360

C -5.411091 -2.203947 1.426594

C -6.422145 -0.562087 -0.164167

C -5.387236 -2.655341 -1.055370

H 1.958375 1.127714 -2.046743

H 0.803788 -0.680402 1.668870

H 2.976655 -1.784714 1.818876

H 4.158497 0.048655 -1.888965

H 6.328771 -3.016283 -1.182680

H 5.268238 -1.968369 -2.136852

H 4.582629 -3.330781 -1.239816

H 7.240013 -1.013689 0.095556

H 6.139056 0.096742 0.935294

H 6.207338 0.082918 -0.833139

H 6.315483 -2.968974 1.295711

H 4.585371 -3.330830 1.327580

H 5.220187 -1.930639 2.216123

H -2.373913 2.137246 -0.645305

H -4.586250 1.129831 -0.466823

H -2.768356 -2.702151 0.223700

H -0.571770 -1.700255 0.063342

H -5.343028 -1.446749 2.216132

H -6.383129 -2.704888 1.517882

H -4.632628 -2.951284 1.613731

H -7.380252 -1.087240 -0.077821

H -6.392203 -0.089053 -1.152366

H -6.408732 0.228992 0.594202

H -6.360593 -3.156892 -0.984312

H -4.610902 -3.420658 -0.951284

H -5.298118 -2.225105 -2.059407

C 1.567286 3.335500 0.926193

C 0.151156 3.492646 0.395714

C -0.893861 3.343612 1.355396

C -0.009962 4.633195 -0.594817

H 1.665727 2.446812 1.555512

H 1.827886 4.209514 1.536709

H -0.766505 2.713484 2.230118

H 2.289050 3.261372 0.108268

H -1.881152 3.757392 1.178844

H 0.248120 5.585894 -0.117604

H -1.039598 4.698456 -0.959455

H 0.651752 4.488701 -1.454428

Zero-point correction= 0.524622 Hartree/Particle

Thermal correction to Energy= 0.552533

Thermal correction to Enthalpy= 0.553478

Thermal correction to Gibbs Free Energy= 0.465677

Sum of electronic and zero-point Energies= -1064.373440

Sum of electronic and thermal Energies= -1064.345529

Sum of electronic and thermal Enthalpies= -1064.344585

Sum of electronic and thermal Free Energies= -1064.432386

The wave number of the imaginary frequency= -506.1955 cm^-1^

Entry **35** (TS2)

C 1.160914 -0.544617 0.050997

N -0.056723 0.216640 0.137046

C 1.711907 -1.148083 1.181696

C 1.813654 -0.695846 -1.179840

C 2.986345 -1.437385 -1.263201

C 2.882166 -1.905948 1.081238

C 3.549718 -2.068890 -0.138587

H 1.395078 -0.221824 -2.061431

H 3.466105 -1.535053 -2.233240

H 1.233651 -1.019667 2.147598

H 3.274516 -2.359957 1.984334

C 4.839858 -2.895825 -0.284653

O -0.000305 1.383599 -0.595008

C -1.298267 -0.474861 0.080512

C -1.459989 -1.723462 0.703630

C -2.411559 0.080592 -0.568709

C -3.633629 -0.586466 -0.572928

C -2.691556 -2.374569 0.684600

C -3.817623 -1.828500 0.052680

H -2.304388 1.020034 -1.088797

H -4.463984 -0.117060 -1.094845

H -0.623778 -2.198134 1.202888

H -2.757605 -3.335787 1.183059

C -5.188705 -2.525265 0.015701

C 0.995599 3.054428 1.278524

C -0.203866 2.945169 0.249079

C -1.488136 2.836004 0.874222

C -0.254299 3.857032 -1.052858

C 1.150702 4.513441 1.780098

C 0.705045 2.204786 2.546017

H -1.623377 2.355448 1.832272

C 2.375155 2.607280 0.740101

H -2.395743 3.092839 0.343516

C 1.095869 4.007158 -1.785160

C -0.769832 5.278099 -0.701763

C -1.249269 3.290486 -2.101914

H -0.924961 2.324169 -2.487836

H -2.264725 3.182434 -1.708797

H -1.308761 3.994083 -2.940659

H 1.813605 4.611593 -1.224031

H 1.546885 3.036077 -2.003907

H 0.926774 4.521357 -2.738837

H -1.753467 5.248358 -0.222258

H -0.094739 5.830803 -0.047206

H -0.871458 5.856894 -1.628076

H 2.371505 1.575290 0.394163

H 2.740493 3.233846 -0.072541

H 3.104995 2.683954 1.555260

H 0.388073 1.190589 2.297557

H 1.617574 2.140500 3.148758

H -0.063559 2.661701 3.177939

H 1.571932 5.178878 1.021283

H 0.197705 4.930834 2.121419

H 1.841699 4.520697 2.632038

C 4.590764 -4.062290 -1.270649

C 5.973617 -1.996291 -0.831322

C 5.307152 -3.492831 1.056255

H 5.530247 -2.713761 1.794222

H 4.558161 -4.167734 1.485901

H 6.223755 -4.073189 0.901971

H 3.796767 -4.722853 -0.904092

H 4.293986 -3.702129 -2.261278

H 5.502144 -4.660572 -1.392105

H 5.721622 -1.567835 -1.807016

H 6.180524 -1.166928 -0.145449

H 6.896268 -2.577297 -0.951102

C -6.252472 -1.612263 0.670458

C -5.589322 -2.801909 -1.453017

C -5.181930 -3.868336 0.770256

H -4.471015 -4.578337 0.332406

H -4.928546 -3.739716 1.828860

H -6.176990 -4.325414 0.721631

H -5.650880 -1.878492 -2.038664

H -4.858520 -3.457380 -1.940438

H -6.570004 -3.292200 -1.497928

H -6.001297 -1.407449 1.717536

H -6.335721 -0.650253 0.153794

H -7.239114 -2.091662 0.644078

Zero-point correction= 0.695860 Hartree/Particle

Thermal correction to Energy= 0.731222

Thermal correction to Enthalpy= 0.732166

Thermal correction to Gibbs Free Energy= 0.629722

Sum of electronic and zero-point Energies= -1300.042578

Sum of electronic and thermal Energies= -1300.007216

Sum of electronic and thermal Enthalpies= -1300.006272

Sum of electronic and thermal Free Energies= -1300.108716

The wave number of the imaginary frequency= -461.3309 cm^-1^

Entry **36** (TS1)

C 2.733339 -1.459074 0.914080

C 1.485683 -0.852370 0.826449

C 1.201437 0.030163 -0.230656

C 2.193751 0.316279 -1.184941

C 3.441894 -0.282718 -1.096690

C 3.698199 -1.169296 -0.049233

H 2.975239 -2.135347 1.724668

H 0.739784 -1.043284 1.589966

H 1.966929 1.006958 -1.988253

H 4.215316 -0.085976 -1.828942

N -0.051315 0.679560 -0.333145

O -0.018551 1.963265 -0.765120

C -1.302273 0.065187 -0.250758

C -1.442969 -1.334258 -0.101143

C -2.701260 -1.907454 -0.013642

C -3.837250 -1.099258 -0.088238

C -3.723198 0.277468 -0.279522

C -2.466348 0.855852 -0.368805

H -0.572445 -1.976532 -0.073823

H -2.817739 -2.978178 0.100842

H -4.620666 0.878058 -0.363571

H -2.365812 1.914159 -0.557249

N 5.015781 -1.804840 0.045188

O 5.205620 -2.592879 0.972750

O 5.853995 -1.512105 -0.808517

N -5.159666 -1.706061 0.013101

O -6.141339 -0.963392 -0.068045

O -5.221388 -2.927352 0.176050

C -0.006852 4.399672 -0.505712

C 0.177790 3.255648 0.472196

C -0.852185 3.062969 1.433607

C 1.601950 3.072345 0.962513

H 0.264938 5.346585 -0.024550

H -1.044294 4.472056 -0.844825

H 2.305944 3.019378 0.128317

H 1.880069 3.930117 1.587833

H -1.842935 3.480094 1.287069

H 1.710159 2.170620 1.571514

H -0.700784 2.428817 2.301723

H 0.635764 4.264633 -1.380703

Zero-point correction= 0.304115 Hartree/Particle

Thermal correction to Energy= 0.325881

Thermal correction to Enthalpy= 0.326825

Thermal correction to Gibbs Free Energy= 0.250795

Sum of electronic and zero-point Energies= -1159.091276

Sum of electronic and thermal Energies= -1159.069511

Sum of electronic and thermal Enthalpies= -1159.068567

Sum of electronic and thermal Free Energies= -1159.144596

The wave number of the imaginary frequency= -476.7673 cm^-1^

Entry **36** (TS2)

C 2.957016 -2.549574 0.203900

C 1.667490 -2.042684 0.238636

C 1.411960 -0.705874 -0.142144

C 2.488408 0.086396 -0.595917

C 3.777177 -0.424211 -0.627678

C 4.010123 -1.737420 -0.219741

H 3.159887 -3.572315 0.497299

H 0.859631 -2.690968 0.552378

H 2.291297 1.090373 -0.942537

H 4.606951 0.179354 -0.974823

N 0.121305 -0.162498 -0.093491

O -0.038818 1.057788 -0.696735

C -1.048428 -0.970702 -0.059041

C -1.884918 -1.048944 -1.184528

C -3.040155 -1.818163 -1.144876

C -3.351940 -2.510632 0.026373

C -2.535184 -2.451462 1.154141

C -1.383406 -1.672877 1.109982

H -1.608498 -0.514369 -2.085325

H -3.694095 -1.903643 -2.004047

H -2.817411 -2.995266 2.047166

H -0.749113 -1.588634 1.986159

N 5.365476 -2.272434 -0.249529

O 5.532690 -3.439472 0.114738

O 6.270479 -1.528007 -0.635954

N -4.574425 -3.322539 0.072510

O -4.813466 -3.936510 1.112743

O -5.286851 -3.339365 -0.931513

C -1.093650 3.311725 -1.015089

C -0.748517 2.435775 0.262313

C -1.885814 1.827249 0.884218

C 0.313848 2.920018 1.330648

C -1.510156 4.743377 -0.596132

C -2.304120 2.722200 -1.783422

C 1.548237 3.649048 0.763574

C -0.377948 3.887130 2.336636

H -2.789251 1.602906 0.336317

C 0.859748 1.740982 2.176554

H -1.847676 1.469778 1.905415

C 0.054988 3.429772 -2.050525

H 0.077291 1.073707 2.548597

H 1.583200 1.142522 1.622877

H 1.377755 2.150783 3.051107

H -0.791249 4.777649 1.861944

H -1.183860 3.395815 2.890478

H 0.366814 4.220470 3.068797

H 2.086574 3.039493 0.034461

H 1.303498 4.609855 0.304413

H 2.240325 3.856364 1.587922

H 0.958147 3.886008 -1.647700

H 0.318262 2.458906 -2.472830

H -0.290632 4.069875 -2.870636

H -2.314321 4.732561 0.147325

H -0.678331 5.331111 -0.199851

H -1.885168 5.271830 -1.480358

H -2.165924 1.663763 -2.011135

H -3.247046 2.844697 -1.240896

H -2.414369 3.257429 -2.732454

Zero-point correction= 0.475476 Hartree/Particle

Thermal correction to Energy= 0.504611

Thermal correction to Enthalpy= 0.505555

Thermal correction to Gibbs Free Energy= 0.414753

Sum of electronic and zero-point Energies= -1394.760491

Sum of electronic and thermal Energies= -1394.731356

Sum of electronic and thermal Enthalpies= -1394.730412

Sum of electronic and thermal Free Energies= -1394.821214

The wave number of the imaginary frequency= -403.1866 cm^-1^

Entry **37** (TS1)

C -3.671070 0.038350 -0.008071

C -2.194209 -0.087683 -0.038071

C -4.488526 -1.072428 0.266646

C -4.299171 1.272313 -0.253787

C -5.687819 1.390463 -0.228799

C -5.877057 -0.954132 0.296869

C -6.484653 0.277964 0.047904

C -1.513251 -0.945305 0.841733

C -1.412022 0.650649 -0.944080

C -0.027795 0.535410 -0.979447

C -0.126671 -1.057232 0.826904

C 0.644935 -0.325875 -0.093927

N 2.056887 -0.417782 -0.108141

O 2.675080 0.647963 -0.682019

C 2.794572 -1.733302 -0.222664

C 3.221880 -2.205751 1.182120

C 1.916367 -2.801392 -0.904538

C 4.051842 -1.530319 -1.086366

C 3.274014 1.914627 0.396213

C 3.407242 2.975301 -0.682325

C 4.495670 1.425114 0.939458

C 2.153773 2.148695 1.391291

H -4.029672 -2.043406 0.431641

H -3.690064 2.152563 -0.439801

H -6.148046 2.357427 -0.415693

H -6.486403 -1.830019 0.504893

H -7.567206 0.370138 0.069242

H -2.075041 -1.502307 1.586798

H -1.901733 1.300758 -1.664344

H 0.552480 1.099105 -1.700118

H 0.360802 -1.691590 1.558852

H 3.812900 -3.126701 1.110788

H 3.836667 -1.444445 1.672324

H 2.359806 -2.413042 1.823757

H 2.502424 -3.718179 -1.031768

H 1.025433 -3.054347 -0.325391

H 1.592029 -2.463330 -1.894451

H 4.568566 -2.492326 -1.177674

H 3.794013 -1.177685 -2.088494

H 4.736920 -0.808936 -0.638056

H 2.449560 3.143044 -1.183337

H 3.737992 3.923326 -0.241550

H 4.142467 2.672352 -1.434414

H 4.514802 0.882913 1.880038

H 5.419194 1.474083 0.370429

H 1.980024 1.257279 2.001467

H 2.425937 2.970281 2.065419

H 1.218994 2.411021 0.890222

Zero-point correction= 0.411333 Hartree/Particle

Thermal correction to Energy= 0.433730

Thermal correction to Enthalpy= 0.434674

Thermal correction to Gibbs Free Energy= 0.359197

Sum of electronic and zero-point Energies= -907.229447

Sum of electronic and thermal Energies= -907.207050

Sum of electronic and thermal Enthalpies= -907.206106

Sum of electronic and thermal Free Energies= -907.281583

The wave number of the imaginary frequency= -552.6477 cm^-1^

Entry **37** (TS2)

C 4.498655 -0.292012 -0.124550

C 3.076984 0.125061 -0.036946

C 5.509687 0.445464 0.515496

C 4.873766 -1.436505 -0.849289

C 6.209083 -1.828051 -0.932189

C 6.844937 0.053193 0.435217

C 7.201454 -1.085476 -0.289673

C 2.547783 0.664767 1.146693

C 2.206811 -0.014771 -1.132370

C 0.873308 0.374376 -1.053652

C 1.210094 1.043286 1.234470

C 0.354154 0.913872 0.133665

N -1.028361 1.309374 0.257267

O -1.832693 0.441838 -0.485131

C -1.311777 2.753470 -0.117379

C -0.423783 3.675577 0.736923

C -1.046683 3.018122 -1.612847

C -2.776853 3.070830 0.206564

C -3.014659 -0.663911 0.306383

C -3.644726 -1.222773 -1.042575

C -3.917910 0.209078 0.985476

C -2.199954 -1.597400 1.292754

H 5.247455 1.348991 1.058968

H 4.107137 -2.038707 -1.328889

H 6.473390 -2.721354 -1.492276

H 7.609677 0.644234 0.932583

H 8.242306 -1.391155 -0.353175

H 3.180491 0.748857 2.025964

H 2.589288 -0.407140 -2.070822

H 0.218168 0.253195 -1.908911

H 0.815511 1.424889 2.170445

H -0.691118 4.717372 0.529271

H -0.580595 3.493098 1.805611

H 0.639680 3.555129 0.516119

H -1.300615 4.055199 -1.860886

H 0.006200 2.862898 -1.869327

H -1.657993 2.360816 -2.238002

H -2.970211 4.131284 0.008698

H -3.457925 2.480551 -0.407218

H -2.994998 2.875280 1.260996

C -4.011858 -0.070203 -2.016543

C -2.726778 -2.177152 -1.834877

C -4.971179 -1.974183 -0.749311

H -3.728019 0.568210 1.986439

H -4.760605 0.655976 0.474073

C -1.799073 -0.807964 2.570740

C -3.091702 -2.770171 1.777429

C -0.905803 -2.223524 0.724878

H -1.090438 -2.918383 -0.093312

H -0.196869 -1.474319 0.380366

H -0.422013 -2.796585 1.525054

H -1.365123 0.164181 2.329379

H -2.651607 -0.651748 3.240288

H -1.058547 -1.388262 3.131839

H -4.067322 -2.422746 2.133491

H -3.252389 -3.524384 1.001470

H -2.590391 -3.271989 2.614298

H -4.651103 0.690986 -1.559695

H -3.124070 0.425937 -2.407400

H -4.570946 -0.492858 -2.859917

H -4.832437 -2.862792 -0.131987

H -5.704815 -1.331428 -0.252153

H -5.410062 -2.302043 -1.699679

H -1.733280 -1.748182 -1.985012

H -2.620680 -3.152221 -1.351491

H -3.168952 -2.359108 -2.821928

Zero-point correction= 0.582875 Hartree/Particle

Thermal correction to Energy= 0.612562

Thermal correction to Enthalpy= 0.613506

Thermal correction to Gibbs Free Energy= 0.523224

Sum of electronic and zero-point Energies= -1142.901030

Sum of electronic and thermal Energies= -1142.871343

Sum of electronic and thermal Enthalpies= -1142.870398

Sum of electronic and thermal Free Energies= -1142.960681

The wave number of the imaginary frequency= -489.5512 cm^-1^

Entry **38** (TS1)

C 3.501594 0.025169 -0.086539

C 2.005396 0.005949 -0.010665

C 4.275880 0.079307 1.082359

C 4.159622 -0.012248 -1.325322

C 5.553801 0.004559 -1.393539

C 5.669918 0.095221 1.015397

C 6.312938 0.058081 -0.223213

C 1.314067 -1.222915 -0.040138

C 1.284137 1.217460 0.081766

C -0.159720 1.207869 0.201881

C -0.127569 -1.240630 0.054288

C -0.845074 -0.033676 0.234955

N -2.266079 -0.144097 0.423697

O -3.009956 0.704407 -0.357277

C -2.856848 -0.276965 1.808399

C -4.177945 -1.051923 1.685400

C -1.898449 -1.045633 2.732089

C -3.134345 1.108868 2.429625

C -3.604310 0.145148 -1.936483

C -4.223440 1.478093 -2.317750

C -4.507917 -0.918749 -1.688890

C -2.343119 -0.195769 -2.708073

H 3.777480 0.108442 2.047662

H 3.570688 -0.053742 -2.237776

H 6.046257 -0.023853 -2.362134

H 6.253097 0.136344 1.931746

H 7.398356 0.070887 -0.276075

C 2.002338 -2.474262 -0.168432

C 1.958934 2.481929 0.072411

C -0.833702 2.469125 0.289273

C -0.795911 -2.501572 -0.064467

H -4.646686 -1.150454 2.671159

H -4.874152 -0.531382 1.023992

H -4.009862 -2.057593 1.285405

H -2.367911 -1.137039 3.717575

H -1.686220 -2.053012 2.365558

H -0.948128 -0.519542 2.862659

H -3.625291 0.986586 3.402043

H -2.207458 1.668357 2.589530

H -3.792300 1.697379 1.785151

H -3.494604 2.285806 -2.200585

H -4.548097 1.456250 -3.364957

H -5.092907 1.702189 -1.692232

H -4.162837 -1.946508 -1.630837

H -5.539834 -0.722980 -1.413819

H -1.881381 -1.115318 -2.339416

H -2.591988 -0.346293 -3.766607

H -1.611344 0.614016 -2.642421

H 3.085811 -2.465800 -0.210385

C 1.322315 -3.659821 -0.248295

H 1.867608 -4.594156 -0.351676

H -1.879580 -2.500723 -0.049196

C -0.099611 -3.671637 -0.215331

H -0.633438 -4.612924 -0.316781

H 3.039160 2.489097 -0.020123

C 1.272432 3.662024 0.170639

H 1.808743 4.607190 0.157764

H -1.913451 2.471713 0.329184

C -0.144065 3.653178 0.278016

H -0.685604 4.593401 0.341774

Zero-point correction= 0.505110 Hartree/Particle

Thermal correction to Energy= 0.532829

Thermal correction to Enthalpy= 0.533773

Thermal correction to Gibbs Free Energy= 0.446413

Sum of electronic and zero-point Energies= -1214.403480

Sum of electronic and thermal Energies= -1214.375761

Sum of electronic and thermal Enthalpies= -1214.374817

Sum of electronic and thermal Free Energies= -1214.462177

The wave number of the imaginary frequency= -585.0257 cm^-1^

Entry **38** (TS2)

C -4.284615 -0.347080 -0.251277

C -2.831448 -0.095865 0.015164

C -5.248206 -0.097058 0.737374

C -4.711282 -0.834518 -1.495824

C -6.065021 -1.066163 -1.745292

C -6.602188 -0.328229 0.488907

C -7.014382 -0.813590 -0.753421

C -2.272873 1.168535 -0.260906

C -2.017900 -1.124983 0.537662

C -0.617278 -0.884286 0.821048

C -0.873392 1.414139 0.004776

C -0.062604 0.398934 0.571977

N 1.297466 0.762978 0.895355

O 2.237927 -0.199640 0.498593

C 1.560518 1.264923 2.307565

C 0.376112 2.117205 2.805103

C 1.761127 0.120293 3.324141

C 2.808263 2.161528 2.283518

C 3.345062 0.056529 -0.849412

C 4.513927 -0.920727 -0.374003

C 3.745592 1.433260 -0.773412

C 2.517717 -0.294072 -2.161646

H -4.929415 0.280764 1.705247

H -3.973836 -1.031926 -2.269323

H -6.377203 -1.443918 -2.715517

H -7.334587 -0.128844 1.266936

H -8.068439 -0.993739 -0.947358

C -3.062468 2.234409 -0.806322

C -2.560617 -2.422298 0.816593

C 0.150521 -1.974262 1.347836

C -0.346433 2.705633 -0.324757

H 0.615428 2.472371 3.813322

H 0.197269 2.992073 2.176306

H -0.551298 1.541755 2.867953

H 2.038493 0.544482 4.296366

H 0.843431 -0.458219 3.462148

H 2.556381 -0.561929 3.018406

H 2.967677 2.597782 3.276393

H 3.703286 1.600645 2.017817

H 2.685074 2.980581 1.567492

C 4.958743 -0.613572 1.078315

C 4.142008 -2.419009 -0.398593

C 5.791169 -0.726757 -1.237490

H 3.123723 2.229716 -1.156439

H 4.599894 1.740429 -0.184126

C 1.681980 0.918636 -2.638981

C 3.461255 -0.622928 -3.348651

C 1.527621 -1.471750 -2.015285

H 1.986279 -2.387376 -1.646011

H 0.701302 -1.222800 -1.352788

H 1.107736 -1.693407 -3.004438

H 1.052144 1.329012 -1.851197

H 2.308671 1.722443 -3.039991

H 1.020639 0.593659 -3.450020

H 4.228945 0.145088 -3.488398

H 3.951178 -1.595227 -3.250787

H 2.862177 -0.659328 -4.266358

H 5.336967 0.405889 1.199620

H 4.149620 -0.768152 1.788520

H 5.781945 -1.287144 1.343430

H 5.680274 -1.070210 -2.265255

H 6.108741 0.320779 -1.264178

H 6.607717 -1.306414 -0.790026

H 3.242177 -2.622119 0.187814

H 3.991200 -2.802053 -1.410881

H 4.964918 -2.996222 0.040187

H -4.114545 2.052400 -0.995053

C -2.516784 3.456766 -1.090835

H -3.136578 4.246937 -1.506150

H 0.709011 2.876590 -0.151385

C -1.134204 3.692146 -0.855349

H -0.701353 4.657494 -1.104373

H -3.609274 -2.597521 0.604713

C -1.789324 -3.426384 1.335442

H -2.225484 -4.401156 1.537100

H 1.205974 -1.819760 1.516310

C -0.412703 -3.197070 1.600825

H 0.200711 -4.001158 1.998997

Zero-point correction= 0.676711 Hartree/Particle

Thermal correction to Energy= 0.711711

Thermal correction to Enthalpy= 0.712656

Thermal correction to Gibbs Free Energy= 0.612157

Sum of electronic and zero-point Energies= -1450.067698

Sum of electronic and thermal Energies= -1450.032697

Sum of electronic and thermal Enthalpies= -1450.031753

Sum of electronic and thermal Free Energies= -1450.132252

The wave number of the imaginary frequency= -487.6530 cm^-1^

Entry **39** (TS1)

C -2.231395 -0.039329 1.571024

C -2.334410 0.032028 0.150297

C -2.909503 1.325262 -0.409938

C -2.929722 -1.179248 -0.553829

H -2.135369 -0.991509 2.081697

H -2.082498 0.857628 2.164351

H -4.005125 1.292763 -0.360104

H -2.623455 1.444565 -1.460189

H -2.575380 2.203225 0.145933

H -4.024530 -1.147920 -0.489009

H -2.598931 -2.124002 -0.116403

H -2.652309 -1.172184 -1.612881

O -0.731402 0.027370 -0.574267

N 0.434805 0.012666 0.181414

C 1.188811 1.311838 0.000708

C 1.084511 -1.344813 0.007190

C 2.161835 1.540422 1.180835

C 0.169241 2.464122 0.068997

C 1.925897 1.427176 -1.351789

C 2.517767 -1.415379 0.565477

C 0.255637 -2.346145 0.836449

C 1.107116 -1.810925 -1.467044

H 0.110197 -1.744866 -1.906678

H -0.764819 -2.423050 0.464426

H 1.790968 -1.211902 -2.074878

H 1.441570 -2.853757 -1.521390

H -0.418286 2.411507 0.990930

H 0.222271 -2.038317 1.886750

H 0.707067 -3.343564 0.782378

H -0.508738 2.454743 -0.782816

H 1.666018 1.307592 2.128979

H 3.231881 -0.808203 0.004586

H 1.233049 1.239631 -2.178338

H 2.855036 -2.454995 0.488968

H 2.561670 -1.133020 1.621017

H 3.079794 0.957266 1.117547

H 0.709393 3.416917 0.066748

H 2.763812 0.729545 -1.437241

H 2.457457 2.595361 1.202974

H 2.332125 2.438880 -1.473380

Zero-point correction= 0.363355 Hartree/Particle

Thermal correction to Energy= 0.381394

Thermal correction to Enthalpy= 0.382338

Thermal correction to Gibbs Free Energy= 0.319882

Sum of electronic and zero-point Energies= -602.402845

Sum of electronic and thermal Energies= -602.384806

Sum of electronic and thermal Enthalpies= -602.383862

Sum of electronic and thermal Free Energies= -602.446318

The wave number of the imaginary frequency= -634.0493 cm^-1^

Entry **39** (TS2)

C 2.029106 1.118612 -0.807499

C 2.497223 0.429870 -2.109635

C 3.187146 1.922178 -0.161671

N 1.399887 0.162380 0.198451

C 2.195308 -1.038701 0.717551

C 3.719724 -0.809392 0.817917

C 0.988491 2.182512 -1.197138

O 0.186286 -0.308652 -0.388635

C 1.974837 -2.315891 -0.121762

C 1.742526 -1.291184 2.168982

H 2.848488 1.183495 -2.824897

H 3.319664 -0.271275 -1.946975

H 1.669187 -0.114923 -2.572932

H 1.446982 2.876879 -1.909606

H 0.113799 1.744438 -1.667991

H 0.676869 2.762668 -0.324299

H 0.941806 -2.657068 -0.072135

H 2.230778 -2.161070 -1.173499

H 2.615076 -3.117548 0.265215

H 2.275597 -2.154212 2.585983

H 1.957776 -0.418046 2.793744

H 0.676331 -1.500629 2.229173

C -1.416747 -0.347848 0.307934

C -1.870473 0.991933 1.047696

C -2.139048 -0.801218 -1.044364

C -1.350073 -1.430732 1.262779

H -1.202404 -2.455669 0.954140

H -1.346967 -1.249185 2.328741

C -0.771063 1.457330 2.034202

C -2.236874 2.187059 0.140069

C -3.138727 0.741189 1.919862

H -4.016626 0.464871 1.334005

H -2.986600 -0.028407 2.680946

H -3.378984 1.672536 2.447448

H -0.597454 0.723720 2.828395

H 0.183882 1.625429 1.539993

H -1.097388 2.385445 2.518799

H -1.434459 2.479108 -0.532505

H -3.134231 1.995365 -0.454638

H -2.459151 3.050829 0.778199

C -1.866124 0.097500 -2.272998

C -1.695041 -2.225590 -1.471515

C -3.671650 -0.876150 -0.837617

H -3.934040 -1.471062 0.043509

H -4.137338 0.108270 -0.742404

H -4.121719 -1.361838 -1.711882

H -2.118679 1.144840 -2.114032

H -0.822308 0.034181 -2.582368

H -2.483199 -0.259455 -3.107089

H -0.609335 -2.304488 -1.535635

H -2.072130 -3.005988 -0.802981

H -2.109123 -2.437151 -2.463977

H 4.149770 -1.700971 1.286866

H 4.212927 -0.691249 -0.148566

H 3.971576 0.039914 1.456181

H 2.941668 2.184856 0.872586

H 4.148200 1.410903 -0.174163

H 3.321002 2.856181 -0.718191

Zero-point correction= 0.535468 Hartree/Particle

Thermal correction to Energy= 0.560569

Thermal correction to Enthalpy= 0.561513

Thermal correction to Gibbs Free Energy= 0.486154

Sum of electronic and zero-point Energies= -838.060356

Sum of electronic and thermal Energies= -838.035256

Sum of electronic and thermal Enthalpies= -838.034312

Sum of electronic and thermal Free Energies= -838.109671

The wave number of the imaginary frequency= -453.2252 cm^-1^

Entry **40** (TS1)

C 2.933955 0.351587 -0.545603

H 2.975635 0.617263 -1.600227

C 1.737548 -0.146317 -0.009036

C 4.075262 0.509790 0.243106

C 4.040758 0.167551 1.594728

C 1.721910 -0.496646 1.350550

C 2.859049 -0.336228 2.142767

H 4.988422 0.899459 -0.199908

H 4.925100 0.289414 2.214730

C 0.523103 -0.327213 -0.923066

H 0.806870 -0.875119 1.789195

H 2.821949 -0.608592 3.194801

H 0.800230 0.079986 -1.899210

N -0.702532 0.429964 -0.538430

C 0.193773 -1.822469 -1.188897

C -0.887053 -1.958278 -2.271263

H -0.180616 -2.249478 -0.253134

C 1.443735 -2.618735 -1.597763

H 2.204130 -2.629511 -0.812312

H 1.902444 -2.198891 -2.503026

H 1.171413 -3.657339 -1.819928

H -0.494274 -1.647540 -3.249040

H -1.754393 -1.332783 -2.048950

H -1.215440 -3.000386 -2.367842

C -0.695208 1.929941 -0.694968

O -1.130647 0.083611 0.731860

C -0.088293 2.310455 -2.058266

C 0.064688 2.651403 0.438109

C -2.159490 2.403651 -0.694465

H -2.641966 2.196681 0.262437

H -2.729776 1.910816 -1.488584

H -2.198826 3.486075 -0.860937

H 1.135281 2.434198 0.413243

H -0.325676 2.344223 1.411798

H -0.063628 3.736383 0.341684

H -0.547244 1.736671 -2.871519

H 0.995828 2.168006 -2.091793

H -0.278372 3.372275 -2.248560

C -2.279895 -2.106765 1.401740

C -2.678891 -0.677642 1.066312

C -3.583990 -0.542735 -0.027827

C -3.004404 0.139235 2.307621

H -2.086056 -2.696862 0.502548

H -1.387561 -2.125977 2.034781

H -3.624119 -1.287441 -0.815806

H -3.093065 -2.597527 1.952035

H -4.182286 0.352110 -0.157439

H -2.125638 0.206127 2.956750

H -3.320695 1.154721 2.053392

H -3.814108 -0.339108 2.872271

Zero-point correction= 0.444945 Hartree/Particle

Thermal correction to Energy= 0.467658

Thermal correction to Enthalpy= 0.468602

Thermal correction to Gibbs Free Energy= 0.394536

Sum of electronic and zero-point Energies= -833.380450

Sum of electronic and thermal Energies= -833.357737

Sum of electronic and thermal Enthalpies= -833.356792

Sum of electronic and thermal Free Energies= -833.430858

The wave number of the imaginary frequency= -614.6008 cm^-1^

The total energy of nuclei and electrons= -834.051910066 Hartree/Particle

Entry **40** (TS2)

C 1.301586 -0.106031 -1.895811

C 2.068875 -0.106701 -0.682666

C 3.010551 1.174217 -0.594463

C 2.637087 -1.538043 -0.305963

H 0.727444 -0.962628 -2.219695

H 1.188188 0.792218 -2.490675

C 3.938104 1.222488 0.638557

C 3.908409 1.269809 -1.858984

C 2.186775 2.485146 -0.552475

C 3.143112 -1.662778 1.155353

C 1.541818 -2.611655 -0.542421

C 3.815422 -1.936477 -1.231495

O 0.946152 0.323606 0.632119

N -0.364370 -0.178538 0.676831

C -0.711217 -0.557426 2.103316

C -3.771617 0.464745 -0.776745

C -0.083964 0.419005 3.125085

C -0.193878 -1.978725 2.403409

C -1.251194 0.679623 -0.175246

C -2.494349 -0.094826 -0.640425

C -1.377262 2.204339 0.189653

H -0.368130 2.451055 0.532134

H 0.884137 -2.062830 2.284160

C -2.331530 2.686162 1.301795

H 1.006393 0.363191 3.103033

C -1.615261 3.041944 -1.085884

H -0.364839 1.456330 2.929404

H -0.675261 -2.713225 1.750148

C -2.328878 -1.427558 -1.059314

H -0.435624 -2.242170 3.440190

H -0.419494 0.161817 4.136505

H 1.383059 -2.800917 -1.609032

H 0.590064 -2.325353 -0.096611

H 1.860779 -3.560887 -0.098599

H 2.546578 -1.068998 1.845387

H 4.184807 -1.353386 1.259499

H 3.095921 -2.710104 1.473738

H 4.724791 -1.364413 -1.028372

H 3.558580 -1.820666 -2.289856

H 4.057001 -2.992967 -1.058898

H 4.739069 0.480432 0.595716

H 3.383662 1.090476 1.570581

H 4.419451 2.207271 0.675863

H 1.706327 2.619306 0.416192

H 1.416041 2.531257 -1.326077

H 2.863287 3.332385 -0.717889

H 3.319198 1.381344 -2.774362

H 4.564306 0.408159 -1.988239

H 4.548084 2.156425 -1.770717

C -3.392084 -2.173167 -1.563521

H -1.348996 -1.883477 -0.974373

C -4.662014 -1.602447 -1.675873

H -3.226728 -3.202470 -1.872503

C -4.841872 -0.278259 -1.284656

H -5.494612 -2.180599 -2.067768

H -5.819920 0.188694 -1.370311

H -0.912036 2.772637 -1.882382

H -2.625845 2.917149 -1.488382

H -1.477473 4.108194 -0.871020

H -3.384519 2.461419 1.111072

H -2.082375 2.277158 2.282107

H -2.248871 3.777302 1.383454

C -2.237808 -0.620886 2.311820

H -3.955133 1.491799 -0.491953

H -0.632572 0.720730 -1.082323

H -2.748779 0.321017 2.119054

H -2.698015 -1.390621 1.689243

H -2.419427 -0.886125 3.359523

Zero-point correction= 0.616928 Hartree/Particle

Thermal correction to Energy= 0.646890

Thermal correction to Enthalpy= 0.647834

Thermal correction to Gibbs Free Energy= 0.559913

Sum of electronic and zero-point Energies= -1069.029553

Sum of electronic and thermal Energies= -1068.999592

Sum of electronic and thermal Enthalpies= -1068.998648

Sum of electronic and thermal Free Energies= -1069.086568

The wave number of the imaginary frequency= -561.5639 cm^-1^

The total energy of nuclei and electrons= -1069.93458011 Hartree/Particle

Entry **41** (TS1)

C -1.278845 1.244278 -0.248035

C -0.551835 0.061890 -0.521761

C -1.209548 -1.181429 -0.367036

C -2.544775 -1.233764 0.024655

C -3.248532 -0.056308 0.294433

C -2.607672 1.181158 0.156054

O 0.701962 0.114883 -0.969240

H -0.770523 2.196474 -0.367735

H -0.658687 -2.088830 -0.596168

H -3.040629 -2.196580 0.121307

H -4.288523 -0.100371 0.606066

H -3.153225 2.098985 0.361789

C 3.210991 -0.148471 -0.791109

C 2.159676 0.055021 0.274119

C 2.038066 1.321518 0.857911

C 1.822543 -1.158204 1.106913

H 4.182475 -0.355653 -0.323380

H 3.311275 0.741065 -1.418677

H 1.669751 -2.037571 0.475006

H 2.651347 -1.376252 1.793491

H 2.452339 2.200240 0.373577

H 0.921515 -0.998673 1.706591

H 1.418947 1.478636 1.736040

H 2.947824 -0.994677 -1.430861

Zero-point correction= 0.201038 Hartree/Particle

Thermal correction to Energy= 0.212254

Thermal correction to Enthalpy= 0.213198

Thermal correction to Gibbs Free Energy= 0.162901

Sum of electronic and zero-point Energies= -463.829865

Sum of electronic and thermal Energies= -463.818649

Sum of electronic and thermal Enthalpies= -463.817705

Sum of electronic and thermal Free Energies= -463.868002

The wave number of the imaginary frequency= -481.0881 cm^-1^

Entry **41** (TS2)

C -4.481621 -0.133283 -0.207426

C -3.689567 -1.219190 -0.597969

C -2.330700 -1.248835 -0.301141

C -1.721154 -0.176804 0.388915

C -2.529654 0.920774 0.762864

C -3.894401 0.930311 0.481534

O -0.438554 -0.204693 0.771756

H -4.139728 -2.052941 -1.131628

H -4.501118 1.776080 0.795917

C 1.938986 -1.221098 0.209268

C 1.066889 -0.059942 -0.403280

C 0.504717 -0.386777 -1.654508

C 1.407158 1.465838 -0.193050

C 3.143911 -1.513009 -0.726906

C 1.129512 -2.539019 0.316498

C 1.360496 1.967381 1.271269

C 2.823175 1.766243 -0.750150

H 0.473820 -1.409360 -2.009151

C 0.422576 2.359142 -0.992013

H -0.030805 0.333997 -2.255675

C 2.486113 -0.938623 1.623147

H 3.243809 -0.150101 1.632385

H 1.684547 -0.670861 2.315333

H 2.969444 -1.846691 2.002487

H 2.816998 -1.797637 -1.732655

H 3.823401 -0.663830 -0.822488

H 3.719970 -2.350805 -0.316080

H 0.298856 -2.430051 1.013848

H 0.731464 -2.877721 -0.645373

H 1.793497 -3.331991 0.679899

H 0.395759 1.756469 1.732008

H 2.138535 1.534074 1.898634

H 1.519618 3.052196 1.269859

H 3.615603 1.325492 -0.138720

H 2.940436 1.409280 -1.779352

H 2.982430 2.851296 -0.753199

H 0.568768 2.278967 -2.074177

H -0.622170 2.130774 -0.764701

H 0.600068 3.406936 -0.727112

H -5.543263 -0.118935 -0.437877

H -1.721179 -2.098162 -0.591700

H -2.068941 1.742409 1.303152

Zero-point correction= 0.372448 Hartree/Particle

Thermal correction to Energy= 0.390922

Thermal correction to Enthalpy= 0.391866

Thermal correction to Gibbs Free Energy= 0.327015

Sum of electronic and zero-point Energies= -699.507365

Sum of electronic and thermal Energies= -699.488891

Sum of electronic and thermal Enthalpies= -699.487947

Sum of electronic and thermal Free Energies= -699.552798

The wave number of the imaginary frequency= -426.5521 cm^-1^

Entry **42** (TS1)

C 2.834436 -0.023471 0.083743

C 2.106100 -1.195831 -0.161287

C 0.751962 -1.153983 -0.481983

C 0.073663 0.080824 -0.593130

C 0.809672 1.263229 -0.344752

C 2.156104 1.204874 -0.011968

H 2.610457 -2.158029 -0.098005

H 0.201678 -2.067906 -0.685341

O -1.202345 0.129626 -0.973340

H 0.292488 2.215040 -0.423475

H 2.700857 2.128264 0.176395

C 4.306755 -0.069377 0.416461

H 4.922190 0.225164 -0.444606

H 4.619633 -1.076020 0.712385

H 4.555869 0.615411 1.235763

C -3.689759 -0.161616 -0.677435

C -2.585339 0.038684 0.334122

C -2.455532 1.297431 0.935962

C -2.197970 -1.182394 1.133701

H -3.455108 -1.000514 -1.337596

H -3.826157 0.733033 -1.290857

H -2.988961 -1.415185 1.858866

H -2.070680 -2.052834 0.483924

H -2.903157 2.178311 0.486444

H -1.268676 -1.022913 1.688714

H -1.797168 1.448882 1.786166

H -4.635054 -0.377925 -0.162659

Zero-point correction= 0.228539 Hartree/Particle

Thermal correction to Energy= 0.241647

Thermal correction to Enthalpy= 0.242591

Thermal correction to Gibbs Free Energy= 0.187178

Sum of electronic and zero-point Energies= -503.120404

Sum of electronic and thermal Energies= -503.107296

Sum of electronic and thermal Enthalpies= -503.106352

Sum of electronic and thermal Free Energies= -503.161765

The wave number of the imaginary frequency= -495.7193 cm^-1^

Entry **42** (TS2)

C -4.141512 -0.092558 -0.060047

C -3.351949 -1.188446 -0.450600

C -1.987685 -1.230570 -0.194021

C -1.341829 -0.157516 0.461354

C -2.130907 0.950119 0.837834

C -3.503076 0.967630 0.594063

H -3.822736 -2.027744 -0.959690

H -1.400291 -2.092835 -0.492468

O -0.048330 -0.197603 0.806222

H -1.652563 1.778787 1.351463

H -4.089013 1.827163 0.913424

C -5.626869 -0.072583 -0.332806

H -6.150185 -0.857907 0.228589

H -5.844350 -0.241754 -1.394909

H -6.072011 0.886821 -0.050223

C 1.768057 1.961693 1.257980

C 1.773676 1.459138 -0.206946

C 1.410113 -0.063265 -0.407133

C 0.814167 -0.383046 -1.646067

C 2.293199 -1.232386 0.175391

C 1.476275 -2.543257 0.309724

C 3.463780 -1.535101 -0.799892

C 2.888608 -0.955561 1.570697

C 3.178721 1.745114 -0.797713

C 0.779774 2.362996 -0.982172

H 2.552075 1.515175 1.868575

H 1.945689 3.043671 1.252577

H -0.261762 2.144152 -0.731925

H 0.972913 3.408796 -0.720174

H 2.112036 -0.683087 2.289127

H 0.267182 0.342638 -2.230765

H 3.651574 -0.172130 1.556164

H 3.349673 2.828378 -0.803819

H 0.900928 2.282306 -2.067390

H 3.980828 1.295090 -0.205985

H 0.763665 -1.405160 -1.999974

H 0.673246 -2.429111 1.037926

H 3.267145 1.388064 -1.829712

H 3.377634 -1.867478 1.933254

H 4.147311 -0.692057 -0.918352

H 1.038842 -2.875203 -0.637298

H 2.146271 -3.343279 0.645876

H 3.100645 -1.816364 -1.794087

H 4.045957 -2.378260 -0.408964

H 0.810685 1.767739 1.741114

Zero-point correction= 0.400029 Hartree/Particle

Thermal correction to Energy= 0.420382

Thermal correction to Enthalpy= 0.421327

Thermal correction to Gibbs Free Energy= 0.351416

Sum of electronic and zero-point Energies= -738.797703

Sum of electronic and thermal Energies= -738.777350

Sum of electronic and thermal Enthalpies= -738.776405

Sum of electronic and thermal Free Energies= -738.846316

The wave number of the imaginary frequency= -447.3836 cm^-1^

Entry **43** (TS1)

C 0.133197 -2.547158 -1.009227

C 1.317841 -1.900114 -0.622887

C 1.322677 -0.652263 -0.004372

C 0.059864 0.026230 0.137546

C -1.149129 -0.733122 0.036986

C -1.070770 -1.983475 -0.598842

C 0.175724 -3.850537 -1.769975

C -2.488870 -0.400409 0.755148

C -2.728309 -1.549103 1.778482

C -3.687879 -0.378646 -0.222057

C -2.496128 0.898829 1.587952

C 2.611491 -0.127431 0.686075

C 3.749376 -1.170047 0.626402

C 2.279447 0.110436 2.181699

C 3.171868 1.183070 0.098933

O 0.039679 1.343769 0.400818

H 2.254591 -2.417184 -0.800245

H -1.982118 -2.555065 -0.755586

H 0.989362 -4.496484 -1.419285

H 0.341411 -3.683304 -2.843499

H -0.762950 -4.405844 -1.669098

H -3.654487 -1.364749 2.336814

H -1.903398 -1.606397 2.497274

H -2.817895 -2.525586 1.294037

H -4.620820 -0.228217 0.335295

H -3.785001 -1.319806 -0.773406

H -3.603828 0.428393 -0.955187

H -3.430964 0.939005 2.161259

H -2.439676 1.802117 0.982188

H -1.663390 0.927520 2.295712

H 4.606677 -0.797541 1.198473

H 4.095009 -1.347847 -0.398921

H 3.453758 -2.130919 1.061005

H 3.175087 0.450319 2.717352

H 1.931022 -0.813397 2.658019

H 1.503005 0.871354 2.292256

H 4.118631 1.432665 0.595230

H 2.479010 2.008756 0.254936

H 3.373018 1.087033 -0.974071

C -0.232419 3.763750 -0.140593

C -0.183419 2.519475 -1.000452

C 0.947461 2.369989 -1.844305

C -1.494195 2.111330 -1.637192

H -0.522473 4.627587 -0.752244

H 0.743090 3.972113 0.307962

H -2.338911 2.261535 -0.964551

H -1.665980 2.723125 -2.531807

H 1.853816 2.940855 -1.684056

H -1.471204 1.062719 -1.948526

H 0.964436 1.613614 -2.623354

H -0.963226 3.648664 0.663553

Zero-point correction= 0.455384 Hartree/Particle

Thermal correction to Energy= 0.478973

Thermal correction to Enthalpy= 0.479917

Thermal correction to Gibbs Free Energy= 0.404669

Sum of electronic and zero-point Energies= -817.370508

Sum of electronic and thermal Energies= -817.346920

Sum of electronic and thermal Enthalpies= -817.345976

Sum of electronic and thermal Free Energies= -817.421224

The wave number of the imaginary frequency= -555.4345 cm^-1^

Entry **43** (TS2)
C -3.180178 -0.295400 1.283917

C -2.691036 0.988838 1.025060

C -1.586135 1.233331 0.202613

C -0.815183 0.087573 -0.215901

C -1.503630 -1.168692 -0.283113

C -2.640345 -1.328950 0.523507

C -4.313281 -0.527270 2.253670

C -1.221738 -2.321323 -1.292179

C -2.513246 -2.488400 -2.143566

C -0.937299 -3.669231 -0.588735

C -0.094391 -2.040798 -2.301554

C -1.433866 2.664772 -0.405204

C -2.798282 3.403345 -0.373934

C -1.059459 2.533197 -1.901407

C -0.432763 3.597114 0.306512

O 0.485334 0.196265 -0.609021

H -3.235232 1.829494 1.442958

H -3.129226 -2.299355 0.555170

H -4.997598 0.328453 2.286980

H -3.942540 -0.681731 3.276753

H -4.897148 -1.415106 1.985950

H -2.359810 -3.264850 -2.903595

H -2.761833 -1.553624 -2.658467

H -3.379340 -2.777838 -1.541866

H -0.848563 -4.468975 -1.334605

H -1.741375 -3.949575 0.099888

H -0.004502 -3.644640 -0.018454

H -0.076954 -2.845290 -3.047561

H 0.885669 -1.991765 -1.835096

H -0.256744 -1.095851 -2.829530

H -2.720631 4.311711 -0.982925

H -3.084544 3.720103 0.635151

H -3.606594 2.789112 -0.783538

H -0.900082 3.526389 -2.339845

H -1.870132 2.047640 -2.457466

H -0.157086 1.938150 -2.041918

H -0.551300 4.622325 -0.067189

H 0.597477 3.302369 0.124276

H -0.604982 3.614933 1.389052

C 2.989777 0.656431 -0.566075

C 1.890513 0.290795 0.521127

C 1.569213 1.388963 1.391568

C 2.028032 -1.041898 1.388886

C 4.413541 0.645200 0.046102

C 2.781309 2.090548 -1.115964

C 2.537749 -2.286026 0.636358

C 3.027959 -0.780038 2.557769

H 1.964325 2.380894 1.241967

C 0.702343 -1.440126 2.081296

H 0.886284 1.266450 2.222269

C 3.000313 -0.261368 -1.810623

H 3.153493 -1.313723 -1.576231

H 2.073773 -0.163918 -2.374835

H 3.829036 0.047384 -2.459244

H 4.479172 1.261048 0.949317

H 4.770609 -0.360056 0.283239

H 5.109052 1.067336 -0.688788

H 1.763028 2.240921 -1.471435

H 3.020073 2.870586 -0.385340

H 3.459815 2.242428 -1.962724

H 1.917717 -2.531447 -0.225911

H 3.573576 -2.183399 0.301964

H 2.506361 -3.144275 1.318448

H 4.030373 -0.511228 2.226805

H 2.671449 0.006971 3.229679

H 3.115200 -1.700554 3.147601

H 0.200829 -0.604724 2.577518

H -0.010263 -1.879708 1.390834

H 0.926980 -2.186701 2.852137

Zero-point correction= 0.626589 Hartree/Particle

Thermal correction to Energy= 0.657605

Thermal correction to Enthalpy= 0.658549

Thermal correction to Gibbs Free Energy= 0.569261

Sum of electronic and zero-point Energies= -1053.022474

Sum of electronic and thermal Energies= -1052.991458

Sum of electronic and thermal Enthalpies= -1052.990514

Sum of electronic and thermal Free Energies= -1053.079802

The wave number of the imaginary frequency= -432.8608 cm^-1^

# 3. The information of olefins and nitroxide radicals for each transition state

The information for the structures of olefins and nitroxide radicals at each transition state. Coordinates was at each transition state calculated at (U)B3LYP/6-31G* level. The total energy of nuclei and electrons was calculated at (U)B3LYP/6-311+G** level.

The only entry **6** at TS1

O -1.088941 1.886713 1.376171

N -0.721144 0.637104 0.979334

C 0.754593 0.443637 0.677311

C 0.704114 -0.172313 -0.771887

C 1.488805 1.809012 0.661791

C 1.421250 -0.470079 1.745701

C -1.639575 0.037585 -0.051102

C -0.682151 -0.829613 -0.891848

C -2.390082 1.108424 -0.885393

C -2.684763 -0.847611 0.678104

C 1.796973 -1.151460 -1.154421

O 2.684035 -0.609788 -2.031604

O 1.897443 -2.303608 -0.787784

C -3.608248 -1.688230 -0.215545

C -1.568205 2.096916 -1.717817

C 0.700943 -1.756765 2.159295

C 2.997930 1.765349 0.381313

H 0.770648 0.662853 -1.471661

H 1.323690 2.295734 1.626990

H 1.007786 2.453816 -0.081076

H 1.570786 0.147114 2.639653

H 2.425830 -0.732757 1.395407

H -0.635036 -1.849646 -0.500717

H -1.003509 -0.895624 -1.936615

H -3.023147 1.675936 -0.196107

H -3.066750 0.569714 -1.559545

H -3.294886 -0.201318 1.319300

H -2.137344 -1.519597 1.347783

H 3.332771 -1.314085 -2.223357

H -4.275175 -1.071986 -0.827672

H -3.042861 -2.341753 -0.890691

H -4.242277 -2.333008 0.403995

H -0.910598 2.699301 -1.085565

H -0.961077 1.599603 -2.483264

H -2.240979 2.785225 -2.243179

H -0.301741 -1.545971 2.541866

H 0.623507 -2.462258 1.329119

H 1.266640 -2.251666 2.957841

H 3.548012 1.221062 1.156158

H 3.230427 1.306307 -0.584151

H 3.393495 2.787628 0.362866

The total energy of nuclei and electrons= -790.431672500 Hartree/Particle

The only olefin at TS1 of entry **6**

C -0.033217 1.461735 0.039531

C -0.001113 0.077111 -0.283154

C 1.292731 -0.649298 0.041484

C -1.261694 -0.699335 0.037796

H -0.973385 1.998823 0.112809

H 0.882392 2.042753 0.099196

H 1.319553 -1.629784 -0.443171

H 1.383598 -0.794726 1.124503

H 2.157760 -0.073126 -0.301032

H -1.210206 -1.722340 -0.345728

H -2.141727 -0.207582 -0.385547

H -1.398224 -0.755294 1.125022

The total energy of nuclei and electrons= -157.248503067 Hartree/Particle

The only entry **6** at TS2

O 1.036038 1.928356 -1.473173

N 0.742822 0.613943 -1.095863

C -0.740688 0.403050 -0.757808

C -0.677394 -0.125513 0.722267

C -1.521439 1.743732 -0.789130

C -1.386750 -0.594202 -1.761267

C 1.648921 0.078535 0.001477

C 0.697093 -0.794679 0.841591

C 2.346630 1.178026 0.842516

C 2.740963 -0.811006 -0.653091

C -1.775244 -1.051147 1.211033

O -2.533345 -0.464074 2.177457

O -1.974805 -2.198156 0.870093

C 3.575682 -1.684145 0.296638

C 1.506281 2.063854 1.768440

C -0.718135 -1.951914 -2.009518

C -3.016008 1.670895 -0.440243

H -0.705571 0.750032 1.371715

H -1.420553 2.189055 -1.779245

H -1.032878 2.441556 -0.101521

H -1.470150 -0.071779 -2.720381

H -2.416087 -0.782399 -1.435827

H 0.645630 -1.812298 0.446111

H 1.025874 -0.866035 1.883736

H 2.901460 1.815553 0.151508

H 3.096160 0.670786 1.461868

H 3.417311 -0.170572 -1.227734

H 2.242421 -1.463783 -1.377143

H -3.188194 -1.137002 2.445831

H 4.181846 -1.093011 0.991612

H 2.950999 -2.360690 0.891069

H 4.266520 -2.306768 -0.283518

H 0.737148 2.618478 1.223938

H 1.017708 1.490348 2.564671

H 2.154200 2.800075 2.258693

H 0.324442 -1.843671 -2.317365

H -0.766434 -2.592430 -1.127449

H -1.246501 -2.469240 -2.819575

H -3.580886 1.065874 -1.157078

H -3.195407 1.267491 0.560485

H -3.441395 2.680919 -0.465802

The total energy of nuclei and electrons= -790.421196386 Hartree/Particle

The only olefin at TS2 of entry **6**

C -0.006606 -1.959649 -0.233446

C 0.007100 -0.543854 -0.501809

C -1.383619 0.118792 -0.076785

C 1.387058 0.116054 -0.079357

H 0.899379 -2.536927 -0.125413

H -0.934080 -2.517658 -0.208362

C -1.465779 1.647037 -0.277030

C -1.689388 -0.176504 1.416063

C -2.572245 -0.481097 -0.875387

C 1.646140 1.538426 -0.618460

C 2.565916 -0.765354 -0.573844

C 1.518173 0.192679 1.463942

H 2.687946 -1.676134 0.021298

H 2.436613 -1.043320 -1.619526

H 3.501046 -0.202706 -0.476142

H 1.592675 1.575866 -1.703395

H 0.961987 2.284698 -0.217371

H 2.657759 1.843733 -0.326342

H 0.903195 0.985449 1.899092

H 1.257788 -0.754684 1.947062

H 2.561352 0.420598 1.715991

H -0.875077 2.198406 0.459548

H -1.150525 1.944426 -1.279663

H -2.506895 1.965976 -0.146925

H -2.542720 -0.193036 -1.924680

H -2.618744 -1.572494 -0.823083

H -3.505058 -0.102215 -0.440802

H -1.725316 -1.251112 1.622132

H -0.968202 0.271883 2.100022

H -2.673630 0.240068 1.663236

The total energy of nuclei and electrons= -393.156353029 Hartree/Particle

The only entry **40** at TS1

C 1.772222 -0.316760 1.230839

C 1.081712 0.044114 0.064577

C 3.005626 -0.968824 1.174293

C 3.576918 -1.272495 -0.061268

C 1.674996 -0.258607 -1.171475

C 2.905318 -0.913636 -1.232058

C -0.253866 0.782791 0.181358

N -1.429354 0.109647 -0.441978

C -0.156811 2.266337 -0.270954

C -1.451283 3.022926 0.060800

C 1.043726 2.981547 0.369983

C -1.962926 -1.143228 0.206166

O -1.230383 -0.099697 -1.796033

C -2.050398 -0.946542 1.731086

C -1.119188 -2.395067 -0.114492

C -3.395350 -1.360300 -0.313028

H 1.339802 -0.080640 2.201309

H 3.517386 -1.237651 2.095053

H 4.536077 -1.781116 -0.112560

H 1.156399 0.002578 -2.085689

H 3.342955 -1.143319 -2.200589

H -0.497519 0.826409 1.246345

H -0.006479 2.271753 -1.355171

H -1.563363 3.136470 1.147735

H -2.330143 2.490023 -0.308748

H -1.437833 4.030395 -0.372766

H 1.997382 2.532186 0.080390

H 0.980496 2.957115 1.466153

H 1.059417 4.034929 0.066087

H -2.562379 -0.010564 1.982214

H -1.068990 -0.954498 2.214314

H -2.627690 -1.770780 2.163714

H -0.122383 -2.339165 0.329601

H -1.007629 -2.510235 -1.195744

H -1.615832 -3.290572 0.278394

H -3.402688 -1.543611 -1.388958

H -4.023774 -0.489063 -0.101448

H -3.838845 -2.234133 0.177511

The total energy of nuclei and electrons= -676.825257532 Hartree/Particle

The only olefin at TS1 of entry **40**

C -1.221237 -0.746959 0.042700

C -0.001006 0.089360 -0.311853

C -0.089705 1.468924 0.039543

C 1.302828 -0.608610 0.044449

H -2.153314 -0.221790 -0.180380

H -1.214760 -1.698158 -0.498176

H -1.050966 1.966241 0.115437

H -1.215475 -0.971216 1.117219

H 0.798043 2.083077 0.140655

H 1.394799 -1.545112 -0.514636

H 2.172013 0.014134 -0.184880

H 1.324382 -0.843470 1.115727

The total energy of nuclei and electrons= -157.245984568 Hartree/Particle

The only entry **40** at TS2

O 2.227348 0.065669 -1.723101

N 1.221793 -0.397782 -0.859569

C 1.858162 -1.067915 0.342376

C -2.110511 0.639936 0.538614

C 3.160197 -0.354610 0.774553

C 2.193148 -2.534199 0.002406

C 0.155173 0.649223 -0.736963

C -1.187896 0.029042 -0.320866

C 0.565257 2.061072 -0.178108

C 0.696948 2.315842 1.337607

C -0.316905 3.162129 -0.806346

C -1.572952 -1.184842 -0.918564

C -2.806683 -1.773374 -0.650047

C -3.706094 -1.157013 0.223007

C -3.351386 0.054945 0.809703

C 0.879446 -1.132970 1.532276

H -1.879757 1.585212 1.010665

H 3.926797 -0.434402 0.000967

H 3.001417 0.707803 0.972777

H 3.548355 -0.816101 1.690007

H 2.881143 -2.620129 -0.835860

H 1.283776 -3.095573 -0.233966

H 2.665188 -3.009899 0.870534

H 0.005399 0.869385 -1.802922

H 1.567425 2.199476 -0.594056

H -0.232027 2.167105 1.894880

H 1.462690 1.696828 1.807538

H 0.996014 3.360440 1.490774

H -0.384046 3.053046 -1.894947

H -1.339552 3.155768 -0.415179

H 0.106831 4.151969 -0.599689

H -0.882762 -1.675219 -1.595627

H -3.067131 -2.715084 -1.127149

H -4.669291 -1.612938 0.436182

H -4.038854 0.556194 1.486531

H 0.539593 -0.157936 1.877590

H -0.000053 -1.735976 1.298288

H 1.402077 -1.614455 2.366570

The total energy of nuclei and electrons= -676.801045062 Hartree/Particle

The only olefin at TS2 of entry **40**

C 0.003829 -1.978793 -0.243606

C -0.012553 -0.563930 -0.485152

C 1.369312 0.108808 -0.069033

C -1.383648 0.121195 -0.079920

H -0.886545 -2.588415 -0.305990

H 0.932717 -2.512592 -0.082984

C 1.420972 1.644751 -0.214742

C 1.710434 -0.232459 1.407947

C 2.545621 -0.433862 -0.917978

C -1.582334 1.548012 -0.655924

C -2.567353 -0.768095 -0.544865

C -1.524957 0.228144 1.460347

H -2.670063 -1.663782 0.076324

H -2.458405 -1.078159 -1.583393

H -3.503661 -0.208220 -0.446771

H -1.146183 1.651615 -1.647696

H -1.146192 2.316554 -0.014971

H -2.652660 1.771528 -0.726134

H -0.857622 0.977159 1.895213

H -1.341060 -0.732359 1.953424

H -2.550699 0.536547 1.699221

H 0.802441 2.161537 0.523079

H 1.123437 1.969136 -1.214617

H 2.453435 1.977045 -0.052013

H 2.516904 -0.037438 -1.932456

H 2.562889 -1.525097 -0.977328

H 3.489031 -0.119337 -0.455487

H 1.830856 -1.308851 1.564167

H 0.962852 0.125189 2.116860

H 2.662586 0.245313 1.669104

The total energy of nuclei and electrons= -393.158607627 Hartree/Particle

# 4. The relationships between E_s_^c^ and DPSH in groups of each skeleton

Figure S1 shows the plots of E_s_^c^ *versus* DPSH in Group D constituted by Entry 5-7.

Figure S1 The plots of E_s_^c^ *versus* DPSH and the fitting line in Group D

Figure S2 shows the plots of E_s_^c^ *versus* DPSH in Group E constituted by Entry 8-15.

Figure S2 The plots of E_s_^c^ *versus* DPSH and the fitting line in Group E

# 5. References

1. Naganuma, J.; Yamazaki, Y.; Gotoh, H. Evaluation method of steric shielding effect around nitroxide radical reaction center based on molecular volume within a virtual ball. *Structural Chemistry*. **2019,** 1-8
